# Ion-combination specific effects driving the enzymatic activity of halophilic alcohol dehydrogenase 2 from *Haloferax volcanii* in aqueous ionic liquid solvent mixtures†

**DOI:** 10.1039/d3su00412k

**Published:** 2024-07-08

**Authors:** Alexandra Schindl, M. Lawrence Hagen, Isabel Cooley, Christof M. Jäger, Andrew C. Warden, Mischa Zelzer, Thorsten Allers, Anna K. Croft

**Affiliations:** a Sustainable Process Technologies Group, Department of Chemical and Environmental Engineering, University of Nottingham Nottingham NG7 2RD UK; b School of Pharmacy, University of Nottingham, University Park Campus Nottingham NG7 2RD UK; c School of Life Sciences, University of Nottingham, Queen's Medical Centre Nottingham NG7 2UH UK; d School of Molecular and Cellular Biology, University of Leeds Leeds LS2 9JT UK; e Astbury Centre for Structural Molecular Biology, Faculty of Biological Sciences, University of Leeds Leeds LS2 9JT UK; f Data Science and Modelling, Pharmaceutical Sciences, R&D, AstraZeneca Gothenburg Pepparedsleden 1 SE-431 83 Mölndal Sweden; g CSIRO Environment, Commonwealth Scientific and Industrial Research Organization (CSIRO), Research and Innovation Park Acton Canberra ACT 2600 Australia; h Department of Chemical Engineering, Loughborough University LE11 3TU UK a.k.croft@lboro.ac.uk; i Advanced Engineering Biology Future Science Platform, Commonwealth Scientific and Industrial Research Organisation (CSIRO), Research and Innovation Park Acton Canberra ACT 2600 Australia

## Abstract

Biocatalysis in ionic liquids enables novel routes for bioprocessing. Enzymes derived from extremophiles promise greater stability and activity under ionic liquid (IL) influence. Here, we probe the enzyme alcohol dehydrogenase 2 from the halophilic archaeon *Haloferax volcanii* in thirteen different ion combinations for relative activity and analyse the results against molecular dynamics (MD) simulations of the same IL systems. We probe the ionic liquid property space based on ion polarizability and molecular electrostatic potential. Using the radial distribution functions, survival probabilities and spatial distribution functions of ions, we show that cooperative ion–ion interactions determine ion–protein interactions, and specifically, strong ion–ion interactions equate to higher enzymatic activity if neither of the ions interact strongly with the protein surface. We further demonstrate a tendency for cations interacting with the protein surface to be least detrimental to enzymatic activity if they show a low polarizability when combined with small hydrophilic anions. We also find that the IL ion influence is not mitigated by the surplus of negatively charged residues of the halophilic enzyme. This is shown by free energy landscape analysis in root mean square deviation and distance variation plots of active site gating residues (Trp43 and His273) demonstrating no protection of specific structural elements relevant to preserving enzymatic activity. On the other hand, we observe a general effect across all IL systems that a tight binding of water at acidic residues is preferentially interrupted at these residues through the increased presence of potassium ions. Overall, this study demonstrates a co-ion interaction dependent influence on allosteric surface residues controlling the active/inactive conformation of halophilic alcohol dehydrogenase 2 and the necessity to engineer ionic liquid systems for enzymes that rely on the integrity of functional surface residues regardless of their halophilicity or thermophilicity for use in bioprocessing.

## Abstract 2

Cooperative ion–ion interactions drive the selectivity of ion–protein interactions for the halophilic protein ADH in aqueous solutions, with water molecules tightly bound to acidic residues playing an influential role.

## Graphical abstract

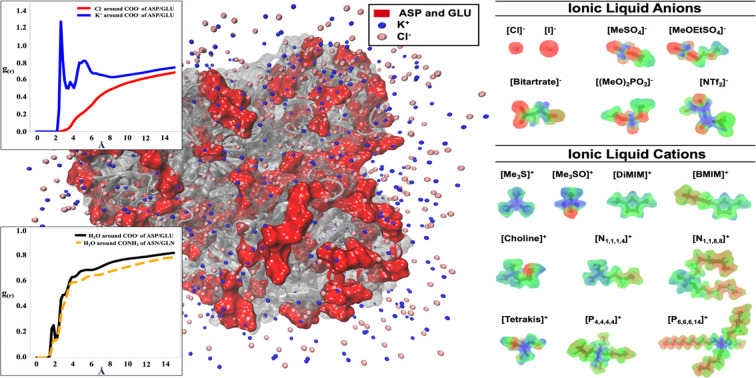

**Sustainability spotlight**

Achieving technoeconomically relevant space–time yields from biocatalysis can often be frustrated by issues in solubility of reagents, stability of the biocatalyst, and general compatibility with process conditions. As such, ionic liquids are being increasingly used to enhance biocatalytic function, although rules for selection of appropriate ionic liquids are not always available, especially for new enzymes. Examining the interactions of ionic liquid ions with halophilic enzyme ADH 2 from *Haloferax volcanii*, as an exemplar for a high-salt tolerant biocatalyst, we identify general trends and mechanisms governed by ion–ion association, with implications for future selection of ionic liquids suitable for biocatalysis. This outcome aligns with the sustainable development goals 9, 12 and 13 by facilitating improved biocatalytic outcomes and consequent utilization of bioprocesses.

## Introduction

Environmentally benign routes to replace existing chemical processes are a high priority to achieve a sustainable and circular economy. Enzymes can be deployed as biocatalysts for the synthesis of pharmaceuticals, biofuels, fine chemicals, and other industrially relevant molecules.^1,2^ Biocatalytic processes are often less energy demanding and polluting than traditional chemical synthetic processes since they function under temperatures and pressures below the boiling point of water, they are selective and have good specificity towards substrates and products, and the catalysts themselves are renewable.^3^ To ensure good utilization of biocatalytic solutions in industry, however, drawbacks such as catalyst instability and difficulties with dissolution of/access to substrates and product recovery need to be addressed.

The use of ionic liquids (ILs) as co-solvents in aqueous media has shown great promise in enhancing biocatalytic outcomes.^4–6^ There are now many studies highlighting how enzyme stability can be improved through the application of ILs,^7–9^ the advantages conferred by improvements in solubility mediated by the tunability of solvation properties of the ILs,^10,11^ and how ILs allow for novel mechanisms for product recovery.^12,13^ Combined with the process advantages of ILs,^14^ these solvents are attractive to further expand the breadth of chemistries available to bioprocessing.

Usually, binary solutions of IL and water are applied for biocatalytic processes, since there are only a few pure ILs known to dissolve enzymes whilst not inactivating them. In addition, many enzymatic reactions in ILs are heterogeneous due to the low solubility.^15^ IL–water mixtures add a layer of complexity compared to the application of pure ILs, since solvent physicochemical properties are significantly altered,^16,17^ but this allows access to an even greater property space. In that sense, deep eutectic solvents, which contain charged and uncharged components, show similar properties to ILs and are similarly applied for biocatalysis.^18^ Being referred to as the 4th generation of ILs, deep eutectic solvents may be well suited for redox biocatalysis particularly to improve the solubility of substrates.^19^ Complexity in IL mixtures stems from specific ion effects, which were first described by Hofmeister for ionic salts,^20^ but ILs have so far defied a quantitative predictive theory. The most widely discussed theories are Collins' law of matching water affinity, where ion pair formation depends on the ions' matching enthalpies,^21^ and Pearson's very similar concept of hard (polarisable) and soft (non-polarisable) acids and bases.^22^ Moreover, specific ion effects have not only been observed in dilute aqueous solutions, but also in pure ILs,^23^ hence ion hydration alone as proposed by Hofmeister cannot explain this effect. Therefore, the exact mechanisms for the interaction between ionic liquid ions and proteins remain poorly understood and finding an adequate system wherein a given biocatalyst remains stable and soluble remains challenging. For this, biocatalysts derived from extremophilic organisms are of substantial interest as they remain functional under harsh conditions, such as high temperatures,^24^ extreme pH^25^ or salinity,^26^ without being specifically engineered. Hence, extremozymes (enzymes derived from extremophiles) act as promising initial candidates for enzyme engineering where challenging reaction conditions are necessary.

To maximise the scope of biosynthetic outcomes, a combination of approaches can be beneficial. Bioprocesses harnessing the inherent properties of halophilic proteins are so far scarce, with the most successful example combining ionic liquids and halophilic cellulases in the saccharification of pretreated lignocelluloses (for examples see ref. 27–33). Studies of a halophilic protease from *Salinivibrio* sp.,^34^ a halophilic phenylalanine dehydrogenase from *Natranaerobius thermophilus*^35^ and an engineered halophilic malate dehydrogenase^36^ found ionic liquid systems wherein the enzyme showed increased activity compared to the free enzyme. Halophilic organisms thrive in high salt environments and have adapted their proteins to intracellular molar concentrations of salt. The main structural adaptations are an excess of negatively charged residues located at the protein surface and a reduction of aromatic hydrophobic residues in the core.^37,38^ A comprehensive study looking at improved IL tolerance of a positively and negatively charge engineered *B. subtilis* lipase A found electrostatic repulsions of both IL ions.^39^ Halophilic proteins might thus be expected to be an existing match to avoid problems with ionic liquid compatibility that are seen in mesophilic enzymes.

This study focuses on the industrially significant enzyme alcohol dehydrogenase, here from the archaeal species *Haloferax volcanii* (*Hv*ADH2). *Hv*ADH2 forms a homo-tetramer under native conditions and is in its tetrameric form around two thirds more active than as a homo-dimer.^40^ The archaeal enzyme has been previously found to exert a preference for haloalkaliphilic conditions (4 M KCl, pH 10) when catalysing the oxidative conversion of alcohol substrates to ketones or aldehydes, and slightly acidic (pH 6) for the catalysis of the reductive reaction.^40^ It exhibits a remarkable thermoactivity with a maximum at 90 °C and its binding pocket can accommodate bulky substrates.^41^ Under bioprocess conditions, pH can be controlled to allow for the tuning of the reaction equilibrium in favour of the desired product. Glycine–KOH buffer has been routinely used to characterise *Hv*ADH2.^40^ The same buffer conditions have been used to characterise enzymatic activity in a range of organic solvents and the enzyme demonstrated remarkable resilience, specifically in dimethyl sulfoxide and methanol.^42^ Commercially, the use of co-solvents is a necessity for certain reactions to afford maximum yields and in this case, if sparingly water-soluble ketones are to be used as substrates, they are indispensable. Substitution of organic co-solvents with ionic liquids that can be more easily recycled and endure higher temperatures therefore has good potential for industrial adaptation.

**Fig. 1:**
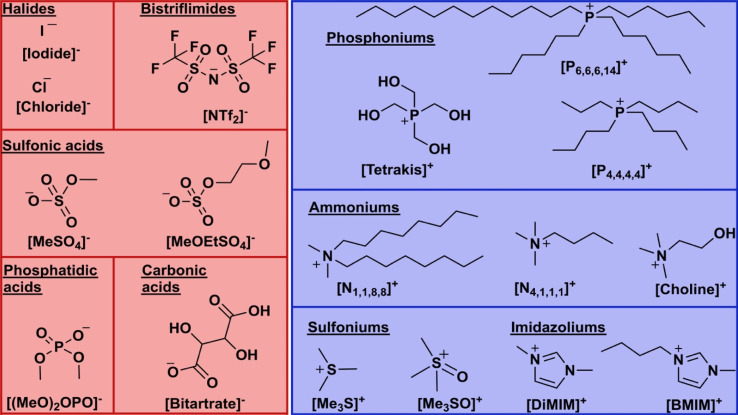
Structures of ionic liquid ions used in this work. Cation classes comprise phosphonium, ammonium, sulfonium and imidazolium ions (blue). Anions comprise halides, bistriflimides, sulfonic acids, phosphatidic acids and carbonic acids (red).

A broad range of different IL ions acting as co-solvent additives were investigated and are depicted in Fig. 1. With regards to MD simulations, these ions represent a wider physicochemical space to study interactions between surface residues and biocatalyst structure and ionic liquid ions than has been reported to date in the literature.^43–62^

We report here both experimental results and extensive molecular dynamics simulations that together shed light on some of the key interactions and considerations needed when using ionic liquid co-solvents, including specific complexities of halophilic systems.

## Results

### Selection of ionic liquids for aqueous mixtures

**Fig. 2:**
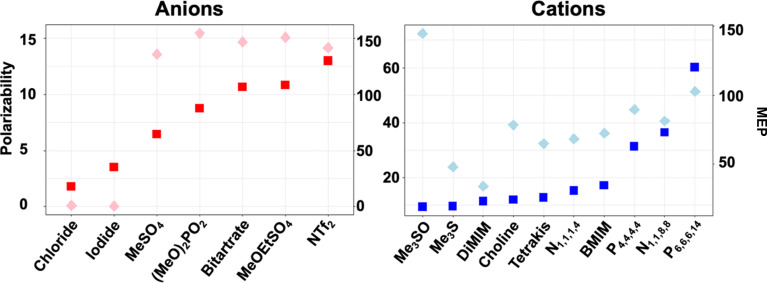
Ion descriptor distribution for increasing polarizability (squares) and MEP_range_ (diamonds) calculated with EMPIRE.^63^

To effectively probe ion–protein interactions, we selected a range of ions and ion combinations according to the two ion descriptors, polarizability and the range of the molecular electrostatic potential (MEP_range_) (Fig. 2). Both have been shown to be of great importance to IL ion behaviour in solution, at surfaces, and in directly influencing reaction kinetics.^35,64,65^ Anions were selected based on their increasing polarizability, starting with monatomic anions [Cl]^−^ and [I]^−^, followed by [MeSO_4_]^−^, [(MeO)_2_OPO]^−^, [bitartrate]^−^, [MeOEtSO_4_]^−^ and [NTf_2_]^−^ in this order. Anions were then combined with different cations diverging in their polarizability and/or MEP_range_. Cations included two imidazolium cations ([DiMIM]^+^ and [BMIM]^+^), hydroxyl-functionalised cations ([tetrakis]^+^ and [choline]^+^), sulfonium based small hydrocarbon cations ([Me_3_S]^+^ and [Me_3_SO]^+^), and phosphonium or nitrogen based cations with medium-to-long alkyl chains ([N_1,1,1,4_]^+^, [N_1,1,8,8_]^+^, [P_4,4,4,4_]^+^ and [P_6,6,6,14_]^+^). Through these combinations we have covered a majority of the descriptor space. A detailed description of these combinations is given in the ESI† section ‘Ionic liquid descriptor space’.

### Activity of *Hv*ADH2 in aqueous ionic liquid solvent systems

**Fig. 3:**
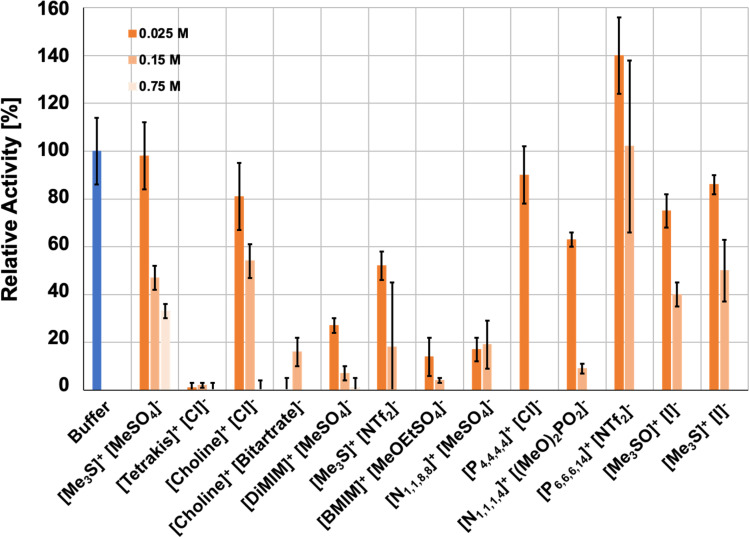
Ionic liquid effect on the activity of *Hv*ADH2 in buffer (blue) and in aqueous ionic liquid mixtures (orange-hues) for all ionic liquid systems (duplicates, error bars indicate estimated standard deviations). Assays containing IL aqueous mixtures were compared to native conditions measured in glycine–KOH buffer at pH 10 containing 4 M KCl at 50 °C. Glycine–KOH buffer was used to make up IL aqueous mixtures for experimental assays, since it has been routinely used in previous studies.^40,42^

Initially, the influence of aqueous ionic liquid mixtures on *Hv*ADH2 was investigated by measuring the effect of added ILs on enzyme activity using UV spectroscopy. The relative activity of *Hv*ADH2 was assayed by monitoring the formation of NADPH (Fig. 3). Ionic liquid concentrations of 25 mM, 150 mM and 750 mM were assayed for [Me_3_S]^+^[MeSO_4_]^−^, [tetrakis]^+^[Cl]^−^, [choline]^+^[Cl]^−^, [DiMIM]^+^[MeSO_4_]^−^, [Me_3_S]^+^[NTf_2_]^−^, [N_4,1,1,1_]^+^[(MeO)_2_OPO]^−^, [P_6,6,6,14_]^+^[NTf_2_]^−^ and [Me_3_S]^+^[I]^−^. For a detailed composition of mixtures see the ESI† section ‘Preparation of ionic liquid mixtures’. The relative activities of *Hv*ADH2 in systems containing imidazolium ions are consistent with studies reporting inhibitory effects on ADH enzymes.^66,67^ In our experiments, sulfonate ions [MeSO_4_]^−^ and [MeOEtSO_4_]^−^, when paired with imidazolium ions, are detrimental for *Hv*ADH2 activity, despite reports of improvements on conversion rates for ADHs in non-imidazolium and imidazolium-paired sulfonate ion ILs.^68,69^ In hydroxy-functionalised ILs [tetrakis]^+^[Cl]^−^ and [choline]^+^[bitartrate]^−^, *Hv*ADH2 showed the lowest activities. Sulfonium based ions appear compatible with *Hv*ADH2, since the enzyme showed the highest tolerance in aqueous ionic liquid mixtures of [Me_3_S]^+^[MeSO_4_]^−^, [Me_3_S]^+^[I]^−^ and [Me_3_SO]^+^[I]^−^ with comparable activity to the buffer system. These activities were eclipsed only by the exceptional activity increase of ∼150% for aqueous mixtures of [P_6,6,6,14_]^+^[NTf_2_]^−^.

Additional and repeat concentrations were measured for systems [Me_3_S]^+^[MeSO_4_]^−^, [P_6,6,6,14_]^+^[NTf_2_]^−^ and [Me_3_S]^+^[I]^−^, since the activity of these initial assays was highest for these aqueous IL mixtures (ESI Fig. S1†). These experiments showed that the enzymatic activity in aqueous mixtures of either [Me_3_S]^+^[MeSO_4_]^−^ or [P_6,6,6,14_]^+^[NTf_2_]^−^ does not follow a continuous decrease with increasing ionic liquid concentration. Activity in [Me_3_S]^+^[MeSO_4_]^−^ plateaued between 150 mM and 300 mM, although the activity was decreased for both compared to the lower concentrations of 25 mM and 75 mM, and activity diminished to below 10% at 600 mM. Despite the formation of an emulsion for all concentrations, the enzymatic activity in [P_6,6,6,14_]^+^[NTf_2_]^−^ also followed a non-continuous concentration dependence. Enzymatic activity decreased between 25 mM and 75 mM but increased at 150 mM compared to 75 mM and increased further at 300 mM, where enzymatic activity was highest. In contrast to [Me_3_S]^+^[MeSO_4_]^−^ and [P_6,6,6,14_]^+^[NTf_2_]^−^, enzymatic activity decreased continuously with increasing concentrations of [Me_3_S]^+^[I]^−^. The non-linear changes in activity for the aqueous mixture of [P_6,6,6,14_]^+^[NTf_2_]^−^ might be rationalised by the relative solubility of the cofactor (Table 1), which preferably remains in the aqueous phase in both its oxidised and reduced forms when compared to the other applied ILs, according to COSMO-RS calculations. The ADH-catalysed reaction shows improvement over buffer and miscible ILs, similar to previous studies with non-polar biphasic systems.^71,72^ An optimum volume-ratio of a water-immiscible IL is thus likely to enhance this effect, and accounts for the non-linearity.

**Table 1:** Calculated relative solubility of NADP^+^ and NADPH (log *P*) values using OpenCOSMO-RS^70^ for infinite dilution in pure ILs compared to pure water

IL	−log *P*_NADPH_	−log *P*_NADP^+^_	
[P_4,4,4,4_]^+^[Cl]^−^	−44.956	−62.975	NADP^+^ more soluble than NADPH in IL
[N_1,1,8,8_]^+^[MeSO_4_]^−^	−19.314	−27.867	
[N_4,1,1,1_]^+^[(MeO)_2_OPO]^−^	−37.684	−45.741	
[Me_3_S]^+^[NTf_2_]^−^	−3.136	−9.493	
[P_6,6,6,14_]^+^[NTf_2_]^−^	+7.038	+1.307	NADPH/NADP^+^ both more soluble in water than IL
[BMIM]^+^[MeOEtSO_4_]^−^	−19.368	−24.832	
**[Choline]** ^**+**^ **[bitartrate]** ^**−**^	**−18.387**	**−18.706**	**NADP** ^**+**^ **and NADPH display similar solubility**
[DiMIM]^+^[MeSO_4_]^−^	−22.901	−21.577	
[Me_3_S]^+^[MeSO_4_]^−^	−21.201	−16.184	
[Tetrakis]^+^[Cl]^−^	−13.708	−7.397	
[Choline]^+^[Cl]^−^	−49.939	−42.073	
[Me_3_S]^+^[I]^−^	−34.748	−19.966	
[Me_3_SO]^+^[I]^−^	−23.702	−6.173	NADPH more soluble than NADP^+^ in IL

Multiple studies have found ADH activity enhanced at very low IL concentrations of different ions but dropping particularly rapidly at higher concentrations.^69,73^ A study on a zinc finger protein proposed a transition point in water structuring, affecting the electrostatic interactions and residence times of ions at the protein surface and consequentially, the secondary structure of the protein.^53,74,75^ The same mechanism could help explain the non-linear decrease in activity in [Me_3_S]^+^[MeSO_4_]^−^ at 300 mM. As such, a molecular-level insight is likely to prove valuable in teasing out these possibilities but will require a comparison of different concentrations of IL [P_6,6,6,14_]^+^[NTf_2_]^−^ and [Me_3_S]^+^[MeSO_4_]^−^.

## MD simulations

Results from activity assays were compared with MD simulations of the enzyme in aqueous solutions containing 4 M KCl and 0.15 M ionic liquid in order to obtain structural insights of specific effects of different IL ions. Comparisons demonstrate good agreement between systems showing a high/low enzymatic activity in assays and IL ion interaction trends with regards to ion specific coordination to residues directly implicated in the substrate conversion mechanism.

### Fluctuations in distance between residues of the catalytic triad of *Hv*ADH2 are increased and become less static demonstrating the impact of IL ions on the active centre

The impact of the presence of ionic liquid ions on the active centre of *Hv*ADH2 was assessed from the distance of residues making up the catalytic triad (Ser_40_, His_59_ and Asp_155_) and the catalytic zinc coordinating residue (Cys_38_) of monomer B (NAD^+^ absent) and monomer D (NAD^+^ bound) (ESI Fig. S5†). While distances of these residues stay approximately within 2 Å for the native system containing 4 M KCl, distances in ionic liquid mixtures are increased for all residues with the exception of Asp_155_–His_59_ in monomer B of [Me_3_S]^+^[MeSO_4_]^−^ and monomer D of [Me_3_SO]^+^[I]^−^ as well as Cys_38_–Ser_40_ of monomer D of [Me_3_SO]^+^[I]^−^. Although Asp_155_–His_59_ of monomer B of [P_4,4,4,4_]^+^[Cl]^−^ and [P_6,6,6,14_]^+^[NTf_2_]^−^ stays within this range too, the distance range over time is less static for these systems in comparison to the native system. For all systems the range between Cys_38_ and His_59_ remains similarly static to the native system with the exception of [P_4,4,4,4_]^+^[Cl]^−^ for monomer B. Overall, an increase in frequency of rearrangements of residues is observed leading to two regimes that by themselves assume similar distance ranges of ∼2 Å as comparable residues in the native system, although frequently switching between these regimes. This is especially visible for the plotted distance for Cys_38_–Ser_40_ in monomer B of [choline]^+^[bitartrate]^−^, [BMIM]^+^[MeOEtSO_4_]^−^ and [P_6,6,6,14_]^+^[NTf_2_]^−^ and is absent for Asp_155_–His_59_ in monomer B of [Me_3_S]^+^[MeSO_4_]^−^, [Me_3_SO]^+^[I]^−^ and [P_6,6,6,14_]^+^[NTf_2_]^−^ and Cl-containing IL systems, as well as Cys_38_–Ser_40_ of monomer D for [P_6,6,6,14_]^+^[NTf_2_]^−^. IL [Me_3_SO]^+^[I]^−^ starts out switching between regimes, but over the course of the simulation becomes similarly static as comparable residues in the native system. Taken together, the active centre is impacted by the presence of IL ions in all systems, but the lowest impact is observed for systems containing [Me_3_S]^+^[MeSO_4_]^−^, [P_6,6,6,14_]^+^[NTf_2_]^−^, [Me_3_SO]^+^[I]^−^ and [Me_3_S]^+^[I]^−^.

### Active-site gating residues are targeted for π-stacking interactions by IL ions

**Fig. 4:**
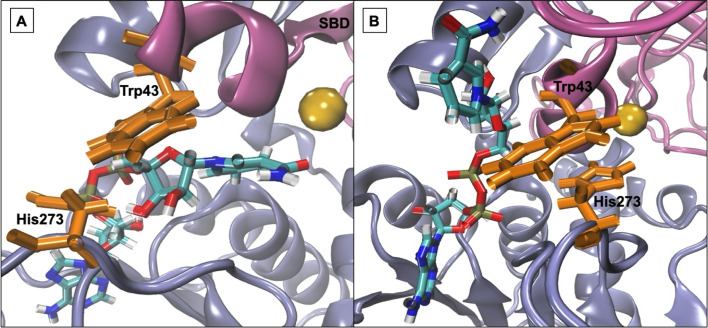
Residues Trp_43_ and His_273_ in monomer A of the native HvADH2 system jointly coordinating NAD^+^ out of the binding pocket. Coordination starts at around 10 ns, and expelling the co-factor is complete after around 20 ns. However, π-stacking between vanguard residues is only established at around 100 ns. (A) NAD^+^ is coordinated in the vicinity of the nicotine binding domain (NBD) (ice-blue) in close proximity to the catalytic zinc, which is located in the substrate binding domain (SBD) (mauve). (B) NAD^+^ has been expelled from the catalytic cleft and residues Trp_43_ and His_273_ have assumed the π-stacking interaction.

Work from Klinman *et al.*^76^ on a thermophilic ADH (*ht*ADH) from *B. stearothermophilus* identified residues Trp_49_ and Phe_272_ as involved in a π-stacking interaction that has a direct effect on the active site microenvironment. These residues are not directly located at the proteins' active site but on the surface, connecting homo-monomers. Closer inspection of the *Hv*ADH2 structure shows a similar π-stacking interaction between residues Trp_43_ and His_273_, albeit within the same monomer (Fig. 4 and S5†). This interaction correlates with the open, or inactive, state of *Hv*ADH2 in the native system and appears to stabilise the apo-enzyme. MD simulations were started with three of the four subunits having NAD^+^ bound (monomers A, C and D), and, over the course of the trajectory, NAD^+^ dissociated from two of the three subunits (monomers A and C), whereby only one subunit expelled the cofactor completely (monomer C). Both vanguard residues Trp_43_ and His_273_ are involved in guiding the cofactor through π-stacking out of the binding pocket. In a first step His_273_ pulls the ribose-ring of NAD^+^ out of the immediate vicinity of the catalytic zinc, followed by a takeover of NAD^+^ by Trp_43_ through the interaction with the pyridinium ring of NAD^+^. NAD^+^ is then passed back to His_273_*via* the ribose-ring and released into an outer cavity on the surface of the nicotinamide binding domain (NBD), where it stays for the rest of the trajectory (monomer A). After NAD^+^ is expelled, residues His_273_ and Trp_43_ assume the π-stacking interaction, which stays undisturbed until the end of the trajectory, and is also found in monomers B and C. In comparison, monomer D remains as a holo-enzyme during the whole simulation and residues Trp_43_ and His_273_ do not assume the π-stacking interaction (ESI Fig. S5B†). By comparison, in aqueous mixtures of IL systems [Me_3_S]^+^[MeSO_4_]^−^, [tetrakis]^+^[Cl]^−^, [P_4,4,4,4_]^+^[Cl]^−^, [P_6,6,6,14_]^+^[NTf_2_]^−^, [Me_3_SO]^+^[I]^−^ and [Me_3_S]^+^[I]^−^ the cofactor does not dissociate from any of the monomers, while in none of the systems do all cofactors dissociate from monomers. In the [choline]^+^[bitartrate]^−^ system the cofactor gets pulled farther into the binding cleft separating the substrate and the nucleotide binding domain in monomer D, while it gets removed to the outer cavity in both other monomers.

**Fig. 5:**
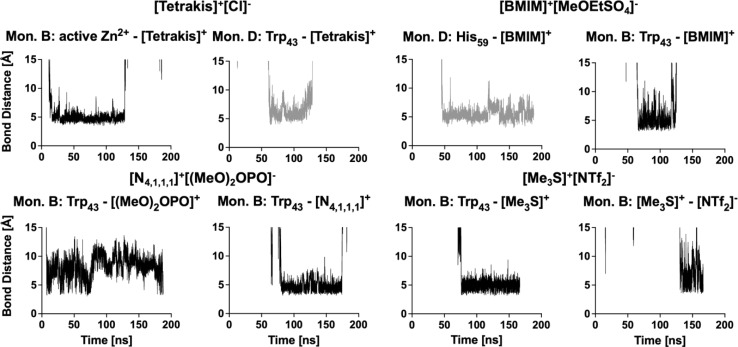
Examples of ions coordinating to gating residues *via* π-stacking over a prolonged time in monomers B (black) and D (grey).

The multimer set-up of the enzyme enables IL ion interactions with the gating residues to be observed for both the closed and open conformations of the enzyme. A close coordination of ions to residues Trp_43_ and His_273_ is seen, disrupting the π-stacking interaction in monomers A, B and C, while coordination to Trp_43_ is also frequent in monomer D (Fig. 5).

**Fig. 6:**
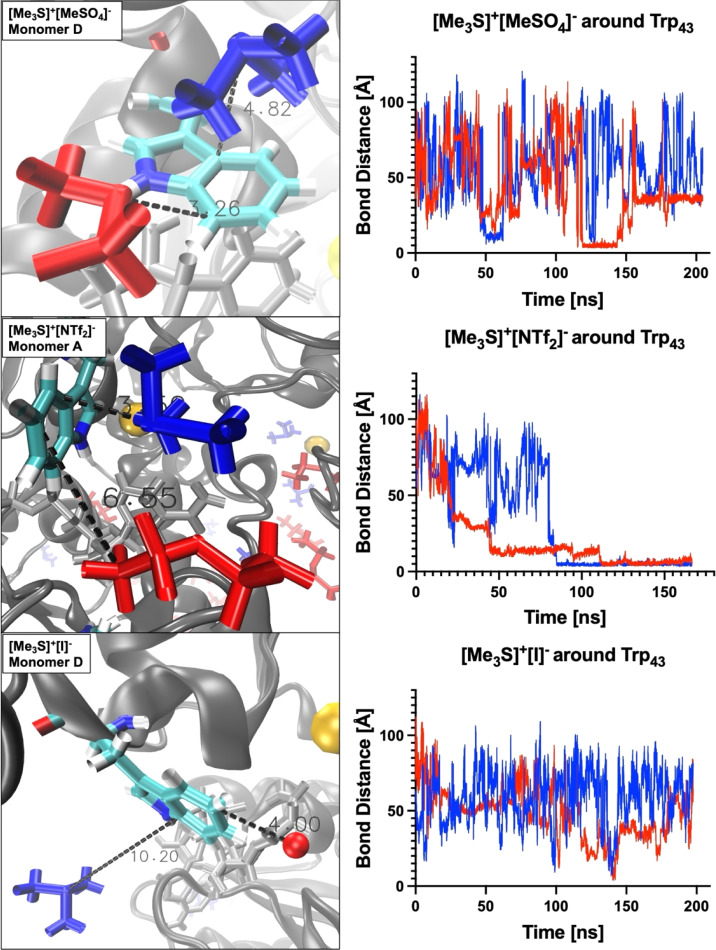
Distances between IL cations (blue) and anions (red) and gating residue Trp43 are depicted, and plotted over the course of the trajectory. Cartoon representation shows tetrameric *Hv*ADH2 in ILs [Me_3_S]^+^[MeSO_4_]^−^ (top), [Me_3_S]^+^[I]^−^ (middle) and [Me_3_S]^+^[I]^−^ (bottom). Plots demonstrate the difference in interaction of cation [Me_3_S]^+^ within the different systems and its dependence on its counter-ion.

Coordination takes place in all IL systems and for all ions; however, in some systems ([Me_3_SO]^+^[I]^−^ and [Me_3_S]^+^[I]^−^) ions coordinate for short times (<5 ns) and infrequently (less than 10 times over the course of the trajectory) to vanguard residues Trp_43_ and His_273_. Other systems ([tetrakis]^+^[Cl]^−^, [N_4,1,1,1_]^+^[(MeO)_2_OPO]^−^, [DiMIM]^+^[MeSO_4_]^−^, [BMIM]^+^[MeOEtSO_4_]^−^, [Me_3_S]^+^[NTf_2_]^−^ and [P_4,4,4,4_]^+^[Cl]^−^) instead exhibit ion coordination over a prolonged time (>50 ns). These interactions take place within the π-stacking distance of 3.7–6 Å.^77^ In turn, oppositely charged ions directly coordinate to the ions which interact with residues Trp_43_ or His_273_, thereby crowding the binding pocket of NAD^+^. These multi-ion arrangements can persist over a prolonged time (∼50 ns). Especially, systems containing [DiMIM]^+^[MeSO_4_]^−^, [BMIM]^+^[MeOEtSO_4_]^−^ and [Me_3_S]^+^[NTf_2_]^−^ show these multi-ion arrangements at gating residues. Furthermore, in the aqueous mixture of [BMIM]^+^[MeOEtSO_4_]^−^ the cation is observed to coordinate preferentially to the catalytic residue His_59_, and in the aqueous mixture of [tetrakis]^+^[Cl] the cation is observed to coordinate to active Zn^2+^ ions. Systems wherein ions associate over a prolonged time to vanguard residues or catalytic residues correlate with lower relative activity from activity assays. In the aqueous mixture of [tetrakis]^+^[Cl]^−^, wherein *Hv*ADH2 showed no activity, [tetrakis]^+^ cations, for example, coordinate throughout the trajectory and stay associated for up to a third of the trajectory (∼60 ns) (Fig. 5). A synergistic effect of ion combinations is demonstrated by sulfonium ion containing IL mixtures (Fig. 6). Sulfonium ions showed high mobility at vanguard residues in systems paired with [I]^−^, while π-stacking was observed for the aqueous mixture of [Me_3_S]^+^[MeSO_4_]^−^. These ions were found in frequent proximity (>10 Å) to vanguard residues and the catalytic zinc, although direct coordination was less frequent and lasted below 5 ns. Contrary to this, in the system paired with the anion [NTf_2_]^−^, [Me_3_S]^+^ shows a >50 ns long interaction with Trp_43_.

To further assess the ion impact on vanguard residues and hence potential ion influence on activity, the change in distance between the two gating residues was analysed and plotted over the course of the trajectory, comparing IL containing systems to the native system (ESI Fig. S6†). The change in distance of the residues in the native system reflects the dissociation of NAD^+^ for monomer A, where rearrangements are greatest in the beginning of the trajectory until expulsion of NAD^+^ is established when the π-stacking interaction fixates the distance between Trp_43_ and His_273_ for the rest of the trajectory. For monomer D, change in distance between Trp_43_ and His_273_ remains roughly constant throughout the trajectory. All IL mixtures distort distances. A distance trajectory similar to that seen for native conditions is observed for monomer A for ILs [P_6,6,6,14_]^+^[NTf_2_]^−^ and [Me_3_S]^+^[MeSO_4_]^−^. The most constant narrow distance is observed for system [Me_3_S]^+^[I]^−^, while the most static systems are [Me_3_S]^+^[NTf_2_]^−^ and [choline]^+^[bitartrate]^−^.

### Secondary structure and free energy landscapes of *Hv*ADH2 are altered in all IL systems

**Fig. 7:**
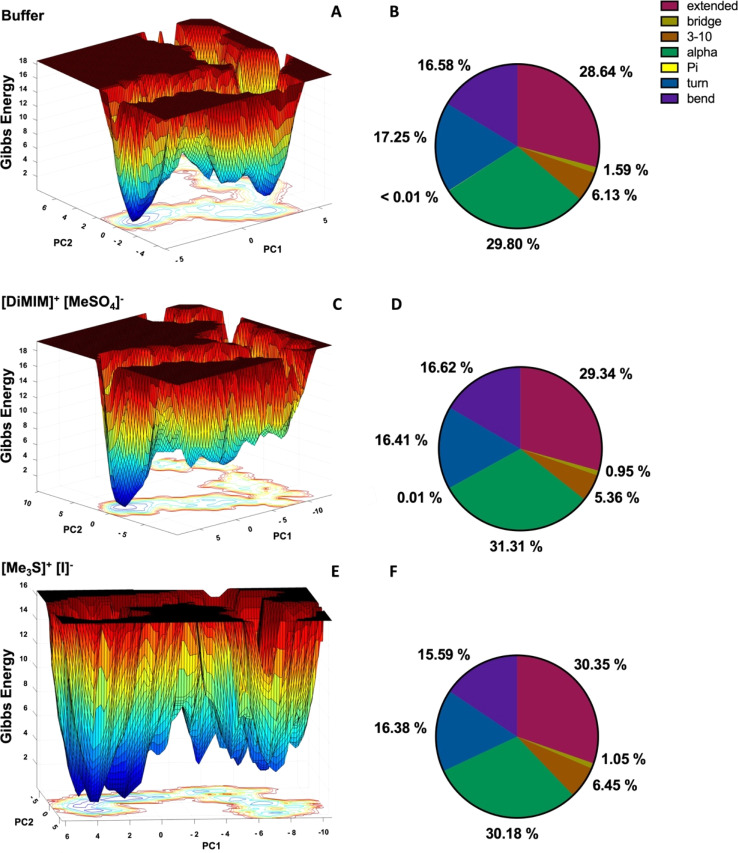
FELs (left, A, C and E) and corresponding secondary structure plots (right, B, D and F) of *Hv*ADH2 in buffer (A and B) and aqueous mixtures of ILs [DiMIM]^+^[MeSO_4_]^−^ (C and D) and [Me_3_S]^+^[I]^−^ (E and F).

To assess the overall structural impact of IL ions on *Hv*ADH2 we derive free energy landscapes (FELs) and assign the secondary structure of *Hv*ADH2 using the DSSP (Dictionary of Secondary Structure in Proteins) algorithm (Fig. 7 and S7†). FELs represent the conformational space the enzyme adopts during simulation. A higher number of separated minima indicates greater flexibility of the protein, but if the barrier between minima is low, it is an indication of a transition into non-native and possibly non-active states. The native system of ADH2 descends into multiple local higher energy minima before occupying the final lowest minimum. The second lowest minimum is separated *via* a higher energy bridge and local minima from the lowest minimum, indicating a stabilisation of the structure followed by structural rearrangements into higher energy states before its final descent (Fig. 7 top). All IL systems force *Hv*ADH2 into non-native states. The general trend shown by the native system is preserved for all IL systems except for [Me_3_SO]^+^[I]^−^, and is best preserved by [Me_3_S]^+^[MeSO_4_]^−^. ILs can be grouped into those preventing ADH2 from reaching stabilising minima before its final descent (Fig. 7 middle) ([choline]^+^[bitartrate]^−^, [DiMIM]^+^[MeSO_4_]^−^, [BMIM]^+^[MeOEtSO_4_]^−^, [P_4,4,4,4_]^+^[Cl]^−^, [P_6,6,6,14_]^+^[NTf_2_]^−^, and [Me_3_SO]^+^[I]^−^) and those that show a broadening and an increased number of troughs of local minima (Fig. 7 bottom) ([Me_3_S]^+^[MeSO_4_]^−^, [tetrakis]^+^[Cl]^−^, [choline]^+^[Cl]^−^, [Me_3_S]^+^[NTf_2_]^−^, [N_1,1,8,8_]^+^[MeSO_4_]^−^, [N_4,1,1,1_]^+^[(MeO)_2_OPO]^−^, and [Me_3_S]^+^[I]^−^). The former appear to trap *Hv*ADH2 in non-native states and the latter appear to destabilise the native state. Secondary structural plots confirm the maintenance of the overall integrity of *Hv*ADH2 throughout the trajectory in all ILs. Matching observations from FELs we find a stabilisation of the helix and bent content in systems such as [DiMIM]^+^[MeSO_4_]^−^ (Fig. 7 middle) and a destabilisation of the helix and bent content in systems such as [Me_3_S]^+^[I]^−^ (Fig. 7 bottom) when compared to the native system (Fig. 7 top).

### Cooperative ion–ion interactions modulate interaction with protein residues

**Fig. 8:**
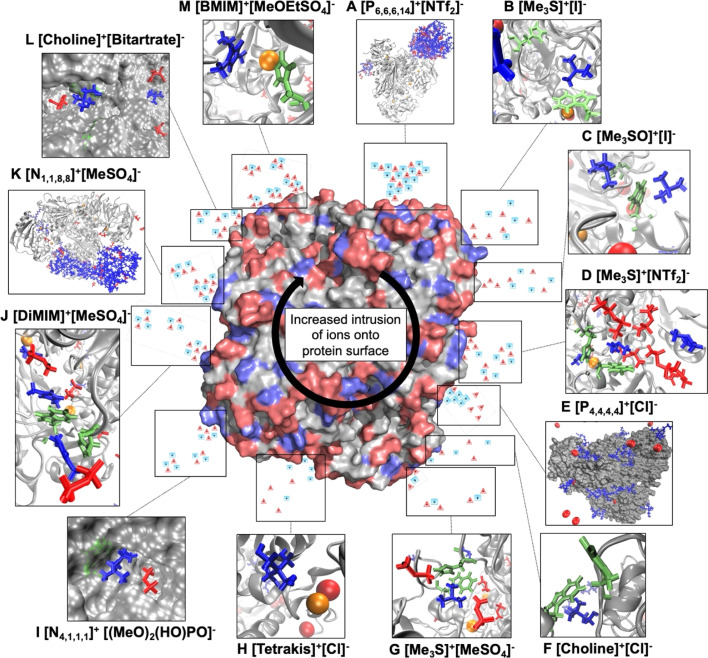
Summary of the general trend of ion–protein and ion–ion interaction inferred from the binding energies of RDFs in combination with SPs and observations from trajectories. Negatively charged residues and anions (triangles) are shown in red, while positively charged residues and cations (squares) are in blue. Functional residues His_59_, Trp_43_ and His_273_ are shown in mint-green. Central surface representation of *Hv*ADH2 shows the proportion of negatively charged (red) and positively charged (blue) residues. Catalytic zinc is shown in orange (sphere).

We derive binding energies (Table 2) from radial distribution functions (RDFs) of IL ions around acidic residues (ESI Fig. S8†) and between IL ion pairs (ESI Fig. S10†) and combine them with survival probability values (SPs) between ions (ESI Fig. S10†) alongside a visual inspection of trajectories to investigate ion–ion interaction effects on ion–protein surface interaction in more detail. We find that binding energies of ions to the protein surface are influenced by the binding energies and survival probabilities to their respective counter-ions (Tables 2 and S4† and Fig. 8). Binding energies are in good agreement with the literature.^78^

**Table 2:** Binding energies are calculated from RDFs and tabulated together with the SP of ion pairs to allow for a comparison between systems with regards to probability and longevity of ion pair or ion cluster formation and the influence of these two factors on the interaction of ions with acidic residues. SP is ranked according to 1 ≙ longest SP. Ion to acidic residue interactions were calculated for monomers

Ionic liquid	Binding energies [kJ mol^−1^]			Inter ion survival probability (ranked)
	Inter ion (one shell)	Ion–acidic residues (combined shells within 15 Å)		
		Cations	Anions	
[P_6,6,6,14_]^+^[NTf_2_]^−^	181.8	2.5 ± 3.9	2.9 ± 4.8	1
[Me_3_S]^+^[I]^−^	10.5	8.3 ± 0.8	21.4 ± 3.4	11
[Me_3_SO]^+^[I]^−^	11.3	8.7 ± 0.5	20.5 ± 2.2	12
[Me_3_S]^+^[NTf_2_]^−^	29.5	9.6 ± 1.1	23.7 ± 5.2	6
[P_4,4,4,4_]^+^[Cl]^−^	5.6	19.2 ± 5.1	4.4 ± 0.3	3
[Choline]^+^[Cl]^−^	11.5	12.4 ± 0.9	4.8 ± 0.2	13
[Me_3_S]^+^[MeSO_4_]^−^	16.2	7.2 ± 0.3	18.2 ± 4.1	10
[Tetrakis]^+^[Cl]^−^	7.9	21.6 ± 2.0	4.8 ± 0.1	9
[N_4,1,1,1_]^+^[(MeO)_2_OPO]^−^	23.1	14.9 ± 1.8	15.3 ± 0.7	5
[DiMIM]^+^[MeSO_4_]^−^	21.7	12.8 ± 1.9	20.7 ± 5.8	7
[N_1,1,8,8_]^+^[MeSO_4_]^−^	118.9	10.1 ± 7.6	16.1 ± 5.4	2
[Choline]^+^[bitartrate]^−^	14.2	11.8 ± 1.9	22.5 ± 7.2	8
[BMIM]^+^[MeOEtSO_4_]^−^	16.3	19.0 ± 3.4	25.1 ± 1.2	4

The largest divergence of binding energy between systems for the same ion is observed for anion [NTf_2_]^−^. Confinement of [NTf_2_]^−^ within the hydrophobic chains of [P_6,6,6,14_]^+^ prevents frequent access of the anion to the protein surface (Fig. 8A). A similar confinement takes place within system [N_1,1,8,8_]^+^[MeSO_4_]^−^ where the cation [N_1,1,8,8_]^+^ has a preference for interacting with the protein surface (Fig. 8K), potentially due to the shortened methyl chains when compared to [P_6,6,6,14_]^+^. Binding energies for anion [MeSO_4_]^−^ diverge between ion combinations too and suggest that in the system [DiMIM]^+^[MeSO_4_]^−^ it is the cation that draws the anion to the surface. A strong ion–ion interaction in systems [Me_3_S]^+^[NTf_2_]^−^ and [Me_3_S]^+^[MeSO_4_]^−^ leads to multi-ion associations with protein residues (Fig. 8D and G). Higher binding energies of anions [NTf_2_]^−^ and [I]^−^ around acidic residues draw the cation [Me_3_S]^+^ closer to acidic residues (Table 2). We further find that hydroxy groups on [tetrakis]^+^ lead to direct coordination of the anion to the Zn^2+^ ion in the active centre (Fig. 8H), while this is not observed for [P_4,4,4,4_]^+^. The stronger binding energy between ions [choline]^+^ and [Cl]^−^ draws the anion to the surface (Table 2 and Fig. 8F). System [N_4,1,1,1_]^+^[(MeO)_2_OPO]^−^ has the most similar binding energies between counter ions, apart from [P_6,6,6,14_]^+^[NTf_2_]^−^, based on a strong ion–ion interaction, suggesting a cooperative interaction with the protein surface (Fig. 8I). A similar cooperative strong interaction with the protein surface is observed for [BMIM]^+^[MeOEtSO_4_]^−^, as well as frequent π-stacking interactions between [BMIM]^+^ and the catalytic His_59_ (Fig. 8M) and gating residues.

Survival probability values reflect the length of interaction between ions and between ions and the protein surface (ESI Fig. S11†). In [P_6,6,6,14_]^+^[NTf_2_]^−^, ions show the longest (highest) SP, and in [choline]^+^[Cl]^−^, ions show the shortest (lowest) SP. In conjunction with binding energies, this reflects on how frequently the exchange of ions on the protein surface takes place and if ions are present as single molecules or in multi-ion associations. For instance, although the interaction between [P_4,4,4,4_]^+^ and [Cl]^−^ is long-lived (third highest SP of all ions), it is a rare encounter (lowest RDF of all ions) (Table 2). Anions and cations of ionic liquid [P_6,6,6,14_]^+^[NTf_2_]^−^ form one big hydrophobic patch that occasionally interacts with a part of the protein surface (Fig. 8A). [Me_3_S]^+^ and [Me_3_SO]^+^ cations and [I]^−^ anions interact as loose ion pairs (Fig. 8B and C) and [NTf_2_]^−^ anions and [Me_3_S]^+^ cations form ion patches (Fig. 8D). We further observe for hydroxyl-functionalised ions [choline]^+^, [tetrakis]^+^ and [bitartrate]^−^ an intrusion below the solvation shell and an interaction with counter-charged protein residues. The association of ions with residues increases with an increased number of OH groups (ESI Fig. S8†). Ions of [choline]^+^[bitartrate]^−^ interact as ion pairs, while [choline]^+^ and [tetrakis]^+^ interact as loose ion pairs with [Cl]^−^ ions. [Me_3_S]^+^ is mostly paired with two [MeSO_4_]^−^ (Fig. 8G). Finally, ions of the systems [N_4,1,1,1_]^+^[(MeO)_2_OPO]^−^, [DiMIM]^+^[MeSO_4_]^−^ and [N_1,1,8,8_]^+^[MeSO_4_]^−^ form ion patches (Fig. 8K and J) and cations of IL [P_4,4,4,4_]^+^[Cl]^−^ form patches on the protein surface (Fig. 8E). Systems wherein cations show the lowest binding energy to acidic residues correlate with the highest activities of *Hv*ADH2 from assays in IL mixtures, while systems containing cations with the highest binding energies to acidic residues impair activity significantly.

### All ILs distort the native solvation shell of *Hv*ADH2

To specifically investigate the impact of IL ions on the solvation shell of the halophilic enzyme we firstly characterised the native system using RDFs and SPs and compared our results to literature reported solvent structure surrounding proteins and single acidic amino acids in high salt. Our results confirm that the presence of K^+^ ions around negatively charged residues ‘breaks’, or rather, mediates the solvation shell, and, through the increased number of acidic residues, allows a cumulative effect to offset salting-out conditions through localised ion association. In addition to literature reports we observe that the protein surface introduces a higher ordering of molecules for the association shell between O_W_ and O_W_, when compared to free floating aspartic amino acid molecules.^79^ We further observe a difference for the RDF between K and O_W_ resulting in peak splitting of the first peak into two solvation shells due to the presence of the protein surface,^79^ and furthermore, a higher ordering into better defined shells of K^+^ arrangements at the protein surface (15 Å) compared to the profile of K^+^–K^+^ within 100 Å. The RDF between potassium and carboxylate groups of Asp/Glu residues determined in this study (ESI Fig. S12†) matches the same positions for all three coordination shell peaks as found in a study by Warden *et al.*, constituting sodium ions in various salts at the surface of an engineered halo-tolerant carbonic anhydrase.^80^ RDF distances between O_W_ and C_4_/C_5_ of Asp/Glu, respectively, match solvation shells found at protein surfaces reported in the literature.^81^ However, in addition, we observe an increase of O_W_ at acidic residues of the first coordination shell at ∼2 Å compared to non-halophilic CALB^81^ as well as at non-acidic surface residues of *Hv*ADH2 (ESI Fig. S13†).

**Fig. 9:**
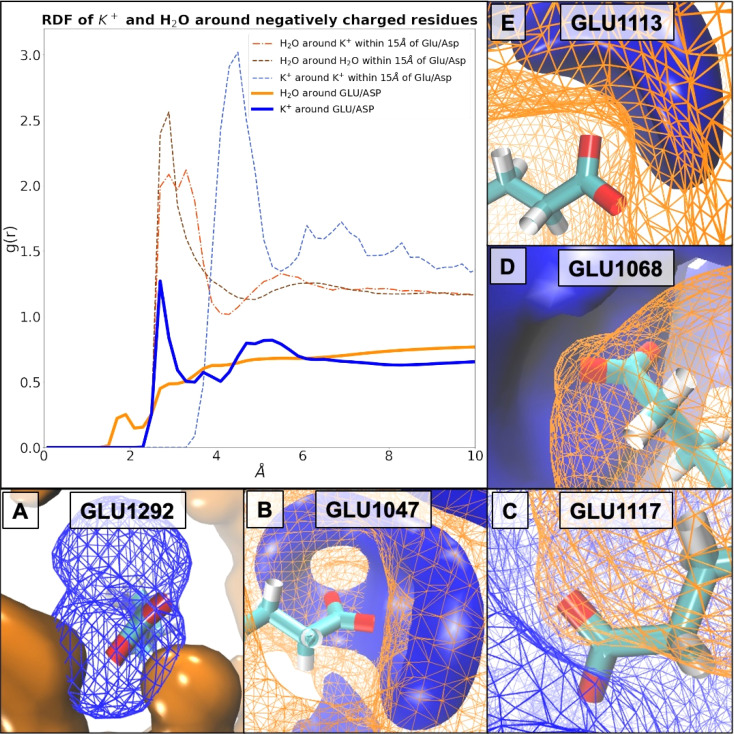
Plotted RDFs and visualised SDFs of K^+^ (blue mesh or solid) and H_2_O (orange mesh or solid) molecules surrounding carboxylic acid residues in the native *Hv*ADH2 system. (A) Direct association of K^+^ with COO^−^ occurs. Directly associated K^+^ at 2.7 Å may account for the distance of the second hydration shell of H_2_O around COO^−^ at 2.9–3.1 Å consistent with the distance between K^+^ and H_2_O in their first hydration shell at 2.9 Å. (B) Neither K^+^ nor H_2_O associate directly. Hydration of COO^−^ is established by the fourth association distance of H_2_O at >4.1 Å, since H_2_O molecules get pulled towards the associated K^+^ ions. (C) Direct association of water with COO^−^ at 1.5–2.1 Å may account for the second coordination shell of K^+^ around COO^−^ at 3.7–4.3 Å, in concordance with the first hydration shell of H_2_O around K^+^ of 2.9 Å. (D) No direct association takes place. K^+^ ions either strip H_2_O molecules partially from COO^−^ residues or are themselves removed behind a water barrier. (E) Direct association of water in its second hydration shell at 2.9 Å with COO^−^ may associate K^+^ ions according to the fourth association distance between H_2_O and K^+^ at 4.5 Å.

Based on our simulations, we calculated probability densities around charged (Fig. 9) and uncharged residues (ESI Fig. S14†). These indicate coordination of water structure by K^+^ at the protein surface regardless of surface charge. For instance, coordination of water around Thr_1073_ by K^+^ demonstrates these site-specific influences particularly well (ESI Fig. S14†). We can demonstrate a direct interaction (∼2.7 Å) between K^+^ ions and specific buried Glu residues (Fig. 9A), which is established over a prolonged time. The very same K^+^ ions stay associated with Glu_1292_ in monomer D, as well as its equivalent in monomer A, Glu_245_, over the whole trajectory (ESI Fig. S15†). This prolonged coordination of K^+^ ions to Glu_245_ and Glu_1292_ is unchanged or only occasionally exchanged in most systems ([Me_3_S]^+^[MeSO_4_]^−^, [choline]^+^[Cl]^−^, [choline]^+^[bitartrate]^−^, [N_1,1,8,8_]^+^[MeSO_4_]^−^, [P_4,4,4,4_]^+^[Cl]^−^, [N_4,1,1,1_]^+^[(MeO)_2_OPO]^−^, and [P_6,6,6,14_]^+^[NTf_2_]^−^), perturbed in some ([tetrakis]^+^[Cl]^−^, [DiMIM]^+^[MeSO_4_]^−^, and [Me_3_SO]^+^[I]^−^) or entirely prevented in others ([Me_3_S]^+^[NTf_2_]^−^, [BMIM]^+^[MeOEtSO_4_]^−^, and [Me_3_S]^+^[I]^−^). These Glu_1292_ associated K^+^ ions in turn coordinate water molecules, which stay associated for about half the trajectories before being exchanged. In systems [Me_3_S]^+^[I]^−^ and [Me_3_SO]^+^[I]^−^, however, in the absence of K^+^, interstitial water molecules still coordinate over a prolonged period of time (∼25 ns). By comparison, acidic surface residues exchange their K^+^ frequently (every few fs). We find that in the native *Hv*ADH2 system the decay of K^+^ around Glu and Asp is slower by approximately a factor of 4 compared to its decay around positively charged residues, thus indicating a prolonged interaction between K^+^ ions and acidic residues (ESI Fig. S16†). Compared to the native system, SPs of K^+^ ions around acidic residues are decreased in all IL systems and increased for all ILs around basic residues, except for hydroxy-functionalised ILs, where they are slightly decreased (ESI Fig. S17†). This indicates faster dynamics of K^+^ ions around acidic residues and slower dynamics of K^+^ ions around basic residues in IL systems. RDFs of K^+^ ions in IL systems show the same profile as the native system, but are increased for all ILs around acidic (Fig. 9) as well as basic residues within 4 Å of the residues (ESI Fig. S18†), while Cl^−^ ions are slightly removed from basic as well as acidic residues (ESI Fig. S19†). This indicates a tighter positively charged ion lattice at the protein surface regardless of charge for all IL systems. Taken together this suggests that all IL systems influence the dynamics of K^+^ around charged residues leading to faster dynamics and a greater total number of K^+^ surrounding negatively charged residues, while mitigating dynamics between K^+^ and positively charged residues.

**Fig. 10:**
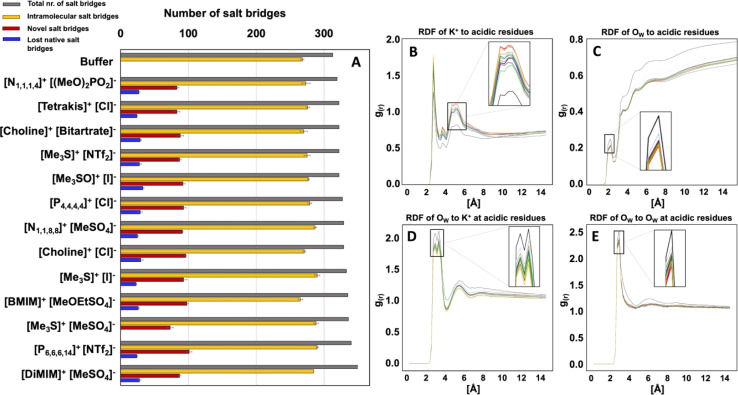
(A) Total number of salt bridges (grey) between protein residues of *Hv*ADH2 monomers calculated by VMD from MD simulations for the native system in high salt (4 M KCl) in comparison to IL systems. Within all IL systems novel salt bridges are formed (red), while some native salt bridges are lost (blue) for all systems, with the exception of IL [Me_3_S]^+^[MeSO_4_]^−^, where none are lost. (B–E) Plots show RDFs of K^+^ and water around acidic residues in IL mixtures (coloured lines) in comparison to the native system (black line). While K^+^ ions are increased around acidic residues in all IL systems, water coordination is overall decreased.

Water has a similar short permanence time (survival probability, SP) around negatively or positively charged residues, indicating that the dynamics (mobility) of the water network is not altered by the prolonged presence of K^+^ ions at carboxylate groups (ESI Fig. S16†). SP of H_2_O around acidic as well as basic residues is decreased in all IL systems (ESI Fig. S20†). RDFs of H_2_O are decreased for all systems around acidic residues, around K^+^ ions and between H_2_O molecules (Fig. 10). In this, the dynamics of H_2_O are altered in all IL systems similarly, becoming generally faster, and molecules become more dispersed.

### ILs compromise the structural integrity of the native protein salt bridge networks

To investigate the impact of altered spatial correlations and residence times of K^+^ and Cl^−^ ions and water molecules on the solvation shell of *Hv*ADH2, the total number of salt bridges of the tetramer (intermolecular and intramolecular) and of the monomers (intramolecular) with an O–N distance cut-off of 3.2 Å was calculated for the native system and compared to IL systems. All ILs lead to an increase in the total number of salt-bridges, but not all native salt-bridges are conserved, with the exception for system [Me_3_S]^+^[MeSO_4_]^−^. New salt-bridges are formed in all ILs (Fig. 10). This implies an interruption of the native salt-bridge network for all IL mixtures and supports the distortion observed in the secondary and tertiary structure of *Hv*ADH2 (Fig. 7).

RDF profiles of H_2_O around acidic residues indicate that all aqueous IL mixtures lead to a decrease in water content around acidic acid residues compared to the native system (Fig. 10). A possible explanation is that water is either displaced on the surface by K^+^ or IL ions, since RDF profiles of K^+^ around acidic residues are increased. This stripping of water molecules and increase of K^+^ ion presence at the protein surface could be an explanation for the overall increase in salt bridges observed for IL systems.

While there are new salt bridges formed in the system [Me_3_S]^+^[MeSO_4_]^−^, none of the native salt bridges are lost. This suggests that the formation of new salt bridges and the loss of native salt bridges are founded in two different ion–water–surface interaction mechanisms. [Me_3_S]^+^[I]^−^ interferes most with the hydration of K^+^ around acidic residues and [choline]^+^[bitartrate]^−^ acts most severely on the water structure itself leading to the greatest difference in RDF of O_W_ around acidic residues compared to the native system. Surprisingly, [Me_3_S]^+^[MeSO_4_]^−^ is one of the systems that interferes most with both water and ion structure. This suggests that the specificity of interactions between IL ions and residues plays a role in compromising native salt bridges and thus potentially the enzyme activity.

## Discussion

**Fig. 11:**
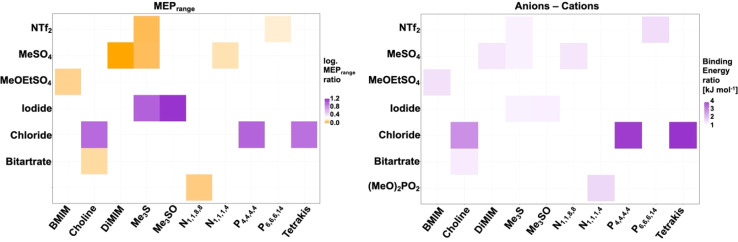
MEP_range_ ratio of anions and cations (left) and ratio of binding energies between anions and cations (right). A greater difference in MEP_range_ between the anion and the cation correlates loosely with a smaller binding energy between paired counter-ions.

Individual ionic liquid ion combinations offer a vast combinatorial property space. However, an outstanding challenge remains to choose an adequate IL system for a given protein and application. Our work provides insight into multifaceted interactions taking place between ions, the solvation shell and protein residues. We are able to show that all IL systems have an influence on the solvation of cofactors, the solvation shell and the secondary structure of the protein. Interaction with residues directly implicated in maintaining activity of the enzyme is affected in all ILs; however, while some ion combinations synergistically prevent co-ions from specifically interacting with these residues, others synergistically coordinate to them. Ions of systems containing ionic liquids [Me_3_S]^+^[I]^−^ and [Me_3_SO]^+^[I]^−^ for instance interact infrequently with gating residues, and [Me_3_S]^+^[MeSO_4_]^−^ does not disrupt native salt bridges; on the other hand, IL systems such as [Me_3_S]^+^[NTF_2_]^−^ form multi-ion arrangements through π-stacking with activity conferring aromatic residues, despite consisting of the same cation. This can be explained by the inter-ion interaction strength through derived binding energies. In turn, binding energies between ions correlate to some degree with MEP_range_ ratios between anions and cations (Fig. 11).

Although we are unable to show a clear correlation between polarizability and MEP_range_ associated with either an increased or decreased enzyme activity, our results demonstrate that cooperative ion–ion interactions strongly influence ion–protein interactions and can help explain enzymatic activity found in assays. We observe for ions that show a high inter-ion binding energy and a high inter-ion SP (close to 1) that these have a close interaction with the protein surface. This surface interaction modulates the overall interaction with protein residues, while the influence of individual ions on the protein surface is greater for ions that share a low SP (close to 0) and low binding energies with one another.

For example, while the strong hydrophobic interaction between [P_6,6,6,14_]^+^ and [NTf_2_]^−^ shields the anion from the protein surface, the comparatively decreased interaction with the cation [Me_3_S]^+^ allows [NTf_2_]^−^ to strongly interact with positive residues at the protein surface. This strong inter-ion interaction, as is the case for [P_6,6,6,14_]^+^[NTf_2_]^−^, can be harnessed to favour biocatalytic processes. Here, phase separation means the protein is mainly excluded from the hydrophobic patch of the IL, whilst providing an advantage for the partition coefficient of the cofactor in the aqueous phase, and hence the agglomeration of [P_6,6,6,14_]^+^ and [NTf_2_]^−^ correlates with a much higher relative activity. This system consists of ions with the highest polarizability and a high MEP_range_. However, as a general trend, a low polarizability and a low MEP_range_ of ions seems to be less detrimental to enzymatic activity of *Hv*ADH2. Small anions of hydrophilic ILs were shown to interact as mediators between water molecules and cations, which are to some extent expelled and form clusters similar to micelles.^82^ The [Me_3_S]^+^[MeSO_4_]^−^ ions, which form ion patches, and [Me_3_S]^+^[iodide]^−^, [choline]^+^[Cl]^−^ and [Me_3_SO]^+^[iodide]^−^, which form loose ion pairs, show low polarizability and rank best in terms of enzymatic activity behind the superior system [P_6,6,6,14_]^+^[NTf_2_]^−^. This suggests that small, highly dynamic cations with low polarizability paired with small charge-dense anions help preserve catalytic activity in *Hv*ADH2 best. By comparison, when paired with a highly polarisable cation ([N_1,1,8,8_]^+^) enzymatic activity is impaired for the anion [MeSO_4_]^−^, unlike in [Me_3_S]^+^[MeSO_4_]^−^. The atomic structure of the ions plays an equally important role. The cation in system [DiMIM]^+^[MeSO_4_]^−^, which forms ion patches similar to [Me_3_S]^+^[MeSO_4_]^−^, has a comparable polarizability to cation [Me_3_S]^+^. However, the presence of the planar structure of [DiMIM]^+^ led to a 5-fold decrease in enzymatic activity when compared to the tetrahedral structure possessing cation [Me_3_S]^+^. The formation of multi-ion structures is driven by entropic forces, since apolar domains minimise the disturbance of the H-bond network of the water molecules. This surfactant effect becomes stronger as the cationic alkyl chain becomes longer and this can affect the stability and activity of proteins positively through suppression of protein–protein interactions, preventing aggregation.^83^ However, if coulombic interactions between ions are strong and moreover if such ions are substituted with hydrophobic tails, strong dispersion forces are observed.^83^ While this does not have immediate implications for proteins if these ion clusters are located in the bulk solvent, it is likely that such ions are expelled from the hydrogen bond network at interfaces and may act as surfactants for solvated proteins. We find this to be the case for systems [N_1,1,8,8_]^+^[MeSO_4_]^−^, [P_4,4,4,4_]^+^[Cl]^−^, [DiMIM]^+^[MeSO_4_]^−^ and [N_4,1,1,1_]^+^[(MeO)_2_OPO]^−^, which form small patches all over the protein surface, and these systems show a decreased enzymatic activity. By comparison, [MeOEtSO_4_]^−^ and [BMIM]^+^ form ion pairs, but because both ions interact strongly with the protein surface, their synergistic effect on the protein is detrimental, since a greatly diminished relative activity (<5 U mg^−1^ at 150 mM) is observed for this system.

Ion–water interactions negatively affected protein–water interactions in all IL systems, and hydroxy-functionalised ILs did not stabilise the surrounding water network. Indeed, hydroxy-functionalised systems performed the worst of all ILs when multiple functionalised groups were present, with only [choline]^+^[Cl]^−^ performing well. This may be due to increased protonation/deprotonation events at the protein surface^84^ or may be a more general outcome for all ILs due to ion type dependent alignment of water at interfaces.^85^ The tight binding of hydration water around carboxylic acid residues in the native system, consistent with previous reports,^79,86–88^ is disturbed in all ILs, as is the selective breaking of water structure induced by the coordination of potassium around carboxylic acid residues.^88^ An increased presence of potassium ions at the protein surface is observed and displaces water molecules, thereby altering the native salt bridge network. Overall, this increases salt bridges in all IL systems. This finding is consistent with an MD study on lactalbumin in different concentrations of [BMIM]^−^[BF_4_]^+^, which found an increase in the number of salt bridges with increasing concentration of IL, and an increased strength of the salt bridge bond.^58^ Inter-subunit salt bridges have been shown to be increased for halophilic proteins when compared to their mesophilic counterparts^89–91^ and it was suggested that oligomerisation acts as a stabilisation mechanism in extremophiles.^92^ This perhaps helps explain the well preserved activity of *Hv*ADH2 in aqueous mixtures of [Me_3_S]^+^[MeSO_4_]^−^, which is the only system wherein none of the native salt bridges are broken.

The negatively charged residues of halophilic protein surfaces cannot protect enzymatic activity from the influence of IL ions. Unlike an engineered *B. subtilis* lipase, where 20 amino acids were specifically selected and substituted, the elevated charge is present throughout the protein surface in *Hv*ADH2 and makes up 27% of the total amino acid content, whereby all charged residues are located on the surface, except one amino acid per monomer.^39,93^ Perhaps this explains the difference in electrostatic repulsion and attraction between *B. subtilis* lipase and *Hv*ADH2, since the same group derived a ranking of amino acid substitutions, which do not nearly cover the surface as extensively as negative charges cover the surface of *Hv*ADH2.^94^ Unlike potassium interacting specifically with carboxylate residues, IL ions interact with the protein surface depending on both their physico-chemical properties and their inter-ion interactions. In contrast to a study where the local structural stability of *B. subtilis* lipase is modified by IL ions inducing long-range perturbations of noncovalent interactions, which eventually reach the active site, we observe a direct interaction with active site residues and apo enzyme structure-determining residues.^95^ However, this does not mean that there are no such long-range perturbations in our system. For enzymes such as dehydrogenases that have a less robust catalytic mechanism, which depends very much on the integrity of their quaternary structure,^40^ and for which the active and inactive conformations rely on the functionality of specific (aromatic) residues located on the surface of the protein, a fine-tuning of IL ions is required in the same way for halophilic as well as non-halophilic proteins.

## Conclusion

Taken together, our data highlights the tuneability of the influence of ions on protein activity depending on co-ion interaction. The same anion or cation exhibits a different influence on the protein when paired with a different co-ion, mainly depending on the strength of ion–ion interaction. The quality of ion–ion interactions depends on polarizability and the resulting molecular electrostatic potential, and binding energies between ions correlate loosely with MEP_range_ ratios between anions and cations. We find that, for halophilic proteins, cations with low polarizability are better suited to maintaining activity; however, ion structure needs to be taken into account, since specific ions and ion-combinations target specific residues, such as aromatics, and thereby elicit a decrease in activity. Future experiments on structure/stability relationships should be undertaken, which will provide a fully conclusive and complementary support for the extensive computational and activity data presented, and provide further illumination of the important roles of ion combinations in protein–solvent interactions.

## Materials and methods

### Reagents and culture conditions

All chemical reagents, unless stated otherwise, were purchased as analytical grade from Sigma-Aldrich. All restriction enzymes were purchased from New England Biolabs. Standard molecular cloning techniques were used. PCR amplification used Q5® Hot Start High-Fidelity DNA polymerase. *H. volcanii* strains were grown at 45 °C on complete (*Hv*-YPC) or Cas-amino acid (*Hv*-Ca) agar or broth as described previously.^96^ Isolation of genomic and plasmid DNA as well as transformation of *H. volcanii* strains were carried out as described previously.^97,98^

### Plasmid construction

All primers were designed using MacVector Version 14.5.2 (MacVector, Inc.) and synthesized by Eurofins MWG, Germany. All plasmids were confirmed by sequencing. Construction of expression-plasmid pTA1205 for 6xHis-ADH2 expression and deletion-plasmids pTA1229 and pTA1230 for the deletion of adh1 and adh2 genes, respectively, were described previously.^40^

### Strain construction

*H. volcanii* Δadh1, Δadh2, ΔtnaA and ΔgabT1 mutant strains were generated using previously described gene knock-out systems.^97,99^*H. volcanii* strain H1895 (ΔpyrE2, Nph-pitA, Δmrr, ΔhdrB, Cdc48d-Ct, ΔpilB3C3)^100^ was the source strain for generating the expression strain H2974 (ΔpyrE2, Nph-pitA, Δmrr, ΔhdrB, Cdc48d-Ct, ΔpilB3C3, Δadh1, Δadh2, ΔtnaA, ΔgabT1). Deletions were confirmed using colony hybridisation and Southern blotting. The *H. volcanii* strain H2974 was transformed with pTA1205 to obtain strain H3094 (ΔpyrE2, Nph-pitA, Δmrr, ΔhdrB, Cdc48d-Ct, ΔpilB3C3, Δadh1, Δadh2, ΔtnaA, ΔgabT1) for overexpression of 6xHis-ADH2.

### Protein expression and purification

An overnight starter culture (5 ml) was diluted (1 : 100) at OD_600_ ∼0.1 into 5 ml and again grown until OD_600_ ∼0.1. Cultures were diluted 1 : 100 in 50 ml and incubated for 24 h at 150 rpm until an OD_600_ of ∼0.5 was reached. Cultures were then diluted (1 : 50) into 333 ml YPC broth and induced with 0.1 g tryptophan and incubated at 150 rpm until an OD_600_ of ∼1.2–1.5 was reached (24–36 h). Cells were harvested by centrifugation and the resultant pellet was either frozen for later use or resuspended in 5 ml wash buffer (20 mM HEPES, pH 7.5, 2 M NaCl). Cells were disrupted by sonication on ice (∼3 × 30 s at 6 W) until the lysate appeared clear. After centrifugation (48 000*g* for 30 min, 4 °C) the supernatant was filtered and loaded onto a HisTrap HP (GE Healthcare) immobilised metal-chelate affinity chromatography (IMAC) column pre-charged with NiSO_4_ (0.2 M) at a flow rate of 0.5 ml min^−1^ using loading buffer (20 mM HEPES, pH 7.5, 2 M NaCl, 20 mM imidazole). Elution buffer (20 mM HEPES, pH 7.5, 2 M NaCl, 50 mM EDTA) was applied to the IMAC column to obtain 2 ml fractions. Fractions were assayed spectrophotometrically for alcohol dehydrogenase activity. Selected fractions were pooled and then dialysed and concentrated using Viva Spin columns (Sartorius) using 3 M glycine–KOH buffer (pH 8). Purified protein samples were analysed by SDS-PAGE and protein concentration was determined using the Bradford protein assay dye reagent (Bio-Rad Laboratories GmbH, Germany).

### Activity assays

Relative activity was assayed spectrophotometrically by monitoring the increase in absorbance of the cofactor NADPH at 340 nm using an Epoch2 Microplate spectrophotometer (Biotek) with UV-transparent 96-well plates. The reaction mixture routinely contained ethanol (100 mM), NADP^+^ (1 mM) and enzyme sample (∼450 nM) and 50 mM glycine–KOH (pH 10.0) buffer containing 4 M KCl. Experiments were carried out at 50 °C for 20 minutes.

### Ionic liquids

Aqueous ionic liquid mixtures used for experimental activity assays as well as MD simulations are summarised in ESI Table S1.† For activity assays [Me_3_S]^+^[MeSO_4_]^−^, [tetrakis]^+^[Cl]^−^, [choline]^+^[Cl]^−^ and [choline]^+^[bitartrate]^−^ were purchased from Acros Organics. [DiMIM]^+^[MeSO_4_]^−^ and [P_6,6,6,14_]^+^[NTf_2_]^−^ were purchased from Fluka. [Me_3_S]^+^[NTf_2_]^−^ was purchased from Solvent Innovation. [BMIM]^+^[MeOEtSO_4_]^−^, [N_1,1,8,8_]^+^[MeSO_4_]^−^ and [N_4,1,1,1_]^+^[(MeO)_2_OPO]^−^ were donated by the Sustainable Process Technologies (SPT) group at the University of Nottingham. [P_4,4,4,4_]^+^[Cl]^−^ was purchased from QUILL. [Me_3_SO]^+^[I]^−^ and [Me_3_S]^+^[I]^−^ were purchased from Sigma Aldrich. All ionic liquids were used as received without further purification.

### Characterisation of *Hv*ADH2 activity in ILs

All ionic liquid mixtures were made up in 50 mM glycine–KOH (pH 10.0) buffer containing 4 M KCl, unless stated otherwise. Oxidative reactions of *Hv*ADH2 were assayed using ethanol (100 mM) and NADP^+^ (1 mM) in aqueous ionic liquid mixtures. For a detailed composition of mixtures see the ESI.†

### Protein sequences and structures

The protein sequence of *Hv*ADH2 was retrieved from the National Centre for Biotechnology (NCBI) database with deposit number ELY36761.1. Homology models were built using Swiss-Model,^101–105^ Phyre2,^106^ and I-TASSER.^107,108^ Models were assessed *via* structural alignment (BLAST) and visualisation (PyMOL). The best model was selected based on the preservation of conserved residues of the catalytic triad coordinating the catalytic zinc (CYS-89, CYS-92, CYS-95 and CYS-103). Following this, the homology model from I-TASSER with the highest C-score (1.62) was chosen to build the tetrameric structure using BIOVIA Discovery Studios Visualizer.^109^ The tetrameric structure of TADH from *Thermus* sp. *Atn1* was retrieved from the protein data bank (RCSB) with the deposit reference 4cpd, and was used as a template to superimpose the monomeric model built by I-TASSER to obtain coordinate positions of the four homo-tetramer units. The geometrically cleaned-up structure was then validated by PROCHECK.^110^ The obtained Ramachandran plot reported the dihedral angles at 74.7% in the most favoured region, 20.1% in the allowed region, 2.8% in the generously allowed region and 2.4% in the disallowed region (see ESI Fig. S21†). The model was then used to create necessary topology files and was processed with AMBER18 (Assisted Model Building with Energy Refinement),^111^ as described below, to run MD simulations. From 200 ns MD time the root mean square deviation (RMSD) was calculated for backbone atoms of the four monomers (ESI Fig. S2†) as well as the root mean square fluctuations (RMSF) (ESI Fig. S3†) to validate the model stability during simulation. RMSF was plotted as standard deviation between the four monomers.

### Molecular dynamics simulation and preparation

The protonation states of amino acid side chains of the I-TASSER *Hv*ADH2 model were adjusted to pH 10 conditions using the H++ server. The software suite used for running all simulations was AMBER18,^111^ whereby xLEaP, antechamber and parmchk were used as preparatory programs, pmemd.cuda 9.2 was used to run simulations and cpptraj was used to transform obtained trajectories for visualisation. To obtain MD simulation input files (.inpcrd and .mdcrd) the routinely applied gaff force field was used.^8,112,113^ The forcefield leaprc.ff14SB was used for non-coordinating protein residues^114^ and the zinc Amber forcefield (ZAFF)^115^ was used for Zn^2+^ coordinating residues, whereby CY4 and HD2 parameters were applied for catalytic residues and CY1 parameters were chosen for structural residues.

A recent study by Daronkola *et al.* found that unoptimized anion–cation parameters for the interaction of potassium with acetate applied in the Amber-GAFF force fields can misrepresent activity derivatives varying with concentration.^116^ They found that contact-shared ion pairs (CIPs) are overestimated, while solvent-shared ion pairs (SIPs) are slightly underestimated. RDF values produced in their study with their newly chosen scaling factor showed the same peak position, but coordination numbers were overall lower for potassium around acetate compared to the amber scaling factor. They still found an increased proximal number density for K^+^ ions and H_2_O molecules around halophilic proteins when compared to their mesophilic counterparts. Use of the amber GAFF force field for the present study thus presents a limitation to quantitation that we acknowledge in the context of this finding. We have thus presented comparative data rather than quantitative, to ensure that our findings remain representative.

Ionic liquid structures were built in Avogadro^117^ and geometry optimised using Gaussian B3LYP/6-31G(d).^118^ The atomic point charges for the ILs were obtained by following the standard restrained electrostatic potential (RESP) fitting procedure,^119^ followed by scaling the charges by applying a factor of 0.8 in accordance with established literature.^112^ Leaprc.gaff was used to produce .mol2 and .lib files and antechamber was used to convert the .mol2 file into a .pdb file. Packmol was used to assemble molecules for simulations in variable numbers reflecting different ion concentrations.^120^ Molecule numbers were calculated according to *N*_ion_ = *N*_A_ × *c*_ion_ (mol l^−1^) × *V* (l), where *N*_ions_ is the number of ions, *N*_A_ is the Avogadro number (6.022 × 10^23^), *c*_ion_ the concentration of the ion and *V* the volume. The protein structure was placed in a quadratic box (100 × 100 × 100 Å) and neutralised by adding K^+^ and Cl^−^ ions using xLEaP. Following neutralisation, ionic liquid molecules were inserted to a concentration of 150 mM (a total number of 80 ion pairs) using packmol. In a final preparation step, the box was solvated with a minimum distance of 2 Å between water molecules using the TIP3P^121^ water model in xLEaP and K^+^ and Cl^−^ ions were added to a concentration of 4 M (total number was around 1800) using the addionsrand command. The forcefield leaprc.gaff with amber parameters sourced from frcmod.ionsjc_tip4pew was used in the production of .mdcrd and .inpcrd files for input in simulations.

Long-range electrostatic interactions and non-bonded interactions were modelled using the particle-mesh Ewald method for periodic boundaries with a cut-off of 10 Å. The first energy minimisation run (50 000 000 steps, steepest descent algorithm for the first 20 000 000 steps, then switched to conjugate gradient algorithm) was performed only on water with a restraining force of 10 kcal mol^−1^ on all other molecules. This was followed by a first heating step of 10 ps to 323.15 K applied to water molecules and a restraining force of 10 kcal mol^−1^ on all other molecules. The overall system was integrated to a temperature isotherm using the Berendsen thermostat with a close coupling of 0.5 ps. A second minimisation step was performed and applied to all atoms using no restraints, followed by a second heating step of the whole system for 0.25 ns under NPT conditions (constant volume in periodic boundaries, Berendsen thermostat) and a subsequent density equilibration step applying Langevin dynamics for 2.5 ns under NTP conditions (constant pressure in periodic boundaries) with restraining forces of 10 kcal mol^−1^ on the protein. Molecular dynamics production run simulations were subsequently run with unrestrained systems for between 150 and 200 ns.

### Analysis of MD simulations

Trajectories were visualised using VMD.^122^ The physical parameters RMSD,^123,124^ SP^125^ and RDFs were calculated using python package MDAnalysis.^126,127^ Principal component analyses on Cα atoms to obtain eigenvalues and eigenvectors were calculated using GROMACS^128,129^ and the first two eigenvectors, PC1 and PC2, which describe >55% of the structural transitions of the overall protein for every system were used to construct free energy landscapes. Post analysis of the molecular dynamics data was graphically represented with Matlab and python packages (MDAnalysis, matplotlib, numpy). TRAVIS was used to calculate SDFs,^130^ which were visualised in VMD with isovalues of 10 for water and 3 for potassium.

### Calculations of local properties and descriptors

The molecular electrostatic potential range (MEP_range_) and the polarizability for IL cations and anions were calculated using the Cepos Insilico software packages EMPIRE.^63^ Cube files were obtained through an eh5cube.sh off the EMPIRE wavefunction output file and visualised in VMD with isovalues of ∼0.02 e Å^−3^.

### Calculation of relative solubilities using OpenCOSMO-RS

The relative solubilities of NADPH and NADP^+^ between water and the ILs were calculated using the COnductor-like Screening MOdel for Real Solvents (COSMO-RS) approach,^131–134^ as implemented in the open source openCOSMO-RS software.^70^ The COSMO-RS method and openCOSMO-RS implementation have been described in detail previously.^135^ In brief, a selection of conformers was generated using the RDKit python package,^136^ followed by a workflow of quantum chemical calculations using the Orca software package,^137,138^ to generate a conformer ensemble for COSMO-RS calculations. To refine the ensemble, conformers were initially eliminated according to energy and similarity. For those conformers that remained, screening charge density was obtained with a COSMO single point calculation using the BP86 method and def2-TZVPD basis set. COSMO-RS calculations were then applied. Populations of conformers in the ensemble were determined according to the Boltzmann distribution by an iterative procedure based on the COSMO-RS chemical potential.^135^ The iterative system was considered converged if, among conformers, the mean chemical potential difference was below 1 × 10^−6^ kJ mol^−1^ and the highest chemical potential difference was below 3 × 10^−6^ kJ mol^−1^. For each IL, the weighted average by population of the converged conformer COSMO-RS chemical potentials, was used to obtain *γ*^inf^(NADPH) and *γ*^inf^(NADP^+^), the infinite dilution activity coefficients of NADPH and NADP^+^, respectively. Calculations were performed with respect to a pure water reference state. Therefore, infinite dilution IL/water partition coefficients are reported as −ln(*γ*^inf^(NADPH)) and −ln(*γ*^inf^(NADP^+^)).

## Data availability

Data are available both within the ESI† and within separate files and repositories as detailed. Data points obtained from measuring spectrophotometrically the increase in NADPH are provided in the Excel sheet named “Experimental_data_ADH2_IL_ActivityAssays.xlsx”. Amber input files for simulations (xxx.prmtop and xxx.inpcrd) alongside files derived from packmol (xxx_box.pdb) and xLEaP (xxx.pdb, HvADH2net.pdb) are deposited at the Zenodo repository under https://doi.org/10.5281/zenodo.10066549. Since xxx.mdcrd files were too big to be deposited (some >50 GB), files were converted into gromacs trajectory files xxx.xtc and xxx.gro using the trajectory converter from the python MDAnalysis suite in Jupyter Notebook. These are deposited at the Zenodo repository under https://doi.org/10.5281/zenodo.4706937. VMD output .dat files of salt-bridges occurring for each step of the trajectory are zipped in the file “saltbridges.zip” and deposited at the Zenodo repository under https://doi.org/10.5281/zenodo.11916740. Scripts derived from the python library ‘MD Analysis’ to calculate RDFs, SPs and RMSDs and the respective data points stored in .csv sheets used to plot RDFs, SPs and RMSDs are zipped in the file “MDAnalysis_Calculated_Plotted_Data.zip”. Calculated MEPrange, polarisability and other ion descriptors in form of .csv files as well as .cube files and others necessary for visualisation of descriptors are contained in “IonDescriptors.zip”. The .lib, .frcmod and .pdb files required to parametrise IL ions for simulations are contained in “ILsource.zip”.

## Author contributions

Alexandra Schindl: conceptualisation (equal), formal analysis (lead), investigation (lead), visualisation (lead), methodology (lead), writing – original draft preparation (lead), writing – review and editing (equal); M. Lawrence Hagen: formal analysis (support), visualisation (support); Isabel Cooley: solubility analysis (lead), solubility methodology (writing); Christof M. Jäger: methodology (support), writing – review and editing (equal); Andrew C. Warden: methodology (support); Mischa Zelzer: conceptualisation (equal), supervision (equal), writing – review and editing (equal); Thorsten Allers: conceptualisation (equal), supervision (equal), methodology (support), writing – review and editing (equal); Anna K. Croft: conceptualisation (equal), supervision (equal), writing – original draft preparation (support), writing – review and editing (equal).

## Conflicts of interest

There are no conflicts to declare.

## Supplementary Material

SU-002-D3SU00412K-s001

SU-002-D3SU00412K-s002

SU-002-D3SU00412K-s003

SU-002-D3SU00412K-s004

SU-002-D3SU00412K-s005
