# Supplementary material for: Ion-combination specific effects driving the enzymatic activity of halophilic alcohol dehydrogenase 2 from Haloferax volcanii in aqueous ionic liquid solvent mixtures
Source: RSC Sustain. 2024 Jul 8;2(9):2559–80. doi: 10.1039/d3su00412k (PMC11353702; doi:10.1039/d3su00412k)
Supplement: SU-002-D3SU00412K-s001 [file SU-002-D3SU00412K-s001.pdf]

Electronic Supplementary Material (ESI) for RSC Sustainability.
This journal is © The Royal Society of Chemistry 2024

Supporting Information

Ion-combination specific effects driving enzymatic activity of halophilic Alcohol Dehydrogenase 2 from *Haloferax volcanii* in aqueous ionic liquid solvent mixtures

Alexandra Schindl,*[a,b,c,d,e] M. Lawrence Hagen,[a] Isabel Cooley[h], Christof M. Jäger,[f,a] Andrew C. Warden,[g] Mischa Zelzer,[b] Thorsten Allers,[c] Anna K. Croft*[h]

[a]*Sustainable Process Technologies group, Department of Chemical and Environmental Engineering, University of Nottingham, Nottingham NG7 2RD, United Kingdom*

[b]*School of Pharmacy, University of Nottingham, University Park Campus, Nottingham NG7 2RD, United Kingdom*

[c]*School of Life Sciences, University of Nottingham, Queen's Medical Centre, Nottingham NG7 2UH, United Kingdom*

[d]*School of Molecular and Cellular Biology, University of Leeds, Leeds LS2 9JT, United Kingdom*

[e]*Astbury Centre for Structural Molecular Biology, Faculty of Biological Sciences, University of Leeds, Leeds LS2 9JT, United Kingdom*

[f]*Data Science and Modelling, Pharmaceutical Sciences, R&D, AstraZeneca Gothenburg, Pepparedsleden 1, SE-431 83 Mölndal, Sweden*

[g]*CSIRO Environment, Commonwealth Scientific and Industrial Research Organization (CSIRO), Research and Innovation Park, Acton, Canberra, ACT, 2600, Australia*

[h]*Department of Chemical Engineering, Loughborough University, LE11 3TU, United Kingdom*

Contents

Supplementary Data Description 3

Ionic liquid descriptor space 5

Table S1 Summary of applied ionic liquids 6

Preparation of ionic liquid mixtures 7

Table S2 Molecular electrostatic potentials for the cations considered 8

Table S3 Molecular electrostatic potentials for the anions considered 9

Relative activity of HvADH2 in aqueous ionic liquid mixtures 10

RMSDs of monomers 11

Standard deviation of RMSF values of four monomers 12

Bond distance of active residues in monomers of the native system 13

Bond distance of active residues in monomers B, C of the native system in comparison to aqueous ionic liquid systems 14

Conformation of gating residues Trp43 and His273 after MD run in the native system **15**
Distance between gating residues Trp43 and His273 over the course of the trajectory in the native system and ionic liquids **16**
Free Energy Landscapes and Secondary Structure Assignment Plots **21**
Table S4 Heatmap binding Energies between IL ions and acidic residues **25**
Radial Distribution Functions (RDFs) of ionic liquid ions around *Hv*ADH2 **26**
Legend for Figures 11, 12 **30**
RDFs and Survival Probabilities (SPs) between IL cations and anions **29**
SPs of cations and anions around *Hv*ADH2 **32**
RDFs of K^+ and O_W around Cγ/Cδ of Glu/Asp residues, Gln/Asn residues and between K^+- K^+, K^+- O_W and O_W- O_W **33**
RDFs of K^+, Cl^- and O_W around Cγ/Cδ of Glu/Asp and Cγ/Cε/Cζ of His/Lys/Arg residues **35**
Spatial distribution functions around non-negatively charged residues **36**
K^+ ion and water association to Glu1292 in monomer D **37**
SPs of O_w and K^+ around Cγ/Cδ of Glu/Asp and Cγ/Cε/Cζ of His/Lys/Arg residues in the native system **38**
SPs of K^+ to Cγ/Cδ of Glu/Asp and Cγ/Cε/Cζ of His/Lys/Arg residues in ionic liquids **39**
RDFs of K^+ to Cγ/Cε/Cζ of His/Lys/Arg residues in ionic liquids **40**
RDFs of Cl^- to Cγ/Cδ of Glu/Asp and Cγ/Cε/Cζ of His/Lys/Arg residues in ionic liquids **41**
SPs of O_w around Cγ/Cδ of Glu/Asp and Cγ/Cε/Cζ of His/Lys/Arg residues in ionic liquids **42**
Ramachandran plot **43**
References **44**

Supplementary Data Description

Experimental data

The data points obtained from measuring spectrophotometrically the increase in NADPH are provided in the excel sheet named "Experimental_data_ADH2_IL_ActivityAssays.xlsx".
Columns have been renamed for identification.

MD amber start files

Amber input files for simulations (xxx.prmtop and xxx.inpcrd) are deposited under https://doi.org/10.5281/zenodo.10066549. Additionally, files derived from packmol (xxx_box.pdb) and xLEaP (xxx.pdb, HvADH2net.pdb) are provided for most of the modelled IL systems. The model of tetrameric ADH2 (HvADH2.pdb), which was built on the homology model of the monomer obtained from I-TASSER and which serves as input to the IL systems built with packmol and xLEaP, is provided therein as well.

MD trajectories

Since xxx.mdcrd files were too big to be deposited (some > 50 GB), files were converted into gromacs trajectory files xxx.xtc and xxx.gro using the trajectory converter from the python MDAnalysis suite in Jupyter Notebook. These are deposited under https://doi.org/10.5281/zenodo.4706937. Due to a lack of matching annotations of Zinc coordinating residues in gromacs, structural and catalytic Zinc coordinating CYS and HIS residues (6 per monomer) are missing from converted trajectories.

H-bond data from intra-protein salt-bridges

VMD output .dat files of salt-bridges occurring for each step of the trajectory are zipped in the file "saltbridges.zip", which is available at https://doi.org/10.5281/zenodo.11916740.

MD Analysis Scripts and exported .csv files

Scripts derived from the python library 'MD Analysis' to calculate RDFs, SPs and RMSDs and the respective data points stored in .csv sheets used to plot RDFs, SPs and RMSDs are zipped in the file "MDAnalysis_Calculated_Plotted_Data.zip".

Cepos InSilico Derived Ion Descriptors

Calculated MEPrange, Polarisability and other ion descriptors in form of .csv files as well as .cube files and others necessary for visualisation of descriptors are contained in “IonDescriptors.zip”.

Parameterised IL ions for MD Simulations

The .lib, .frcmod and .pdb files required to parametrise IL ions for simulations are contained in “ILsource.zip”.

Ionic liquid descriptor space

The cation $[Me_3S]^+$ was selected based on its tetrahedral structure, which we theorised might be least disruptive to water structuring, and because it shows a remarkably low polarisability and a low range between the most negative and most positive molecular electrostatic potential (in the following referred to as MEP_{range}). In order to cover a wide experimental space, this cation was paired with a monatomic anion showing low polarisability and a low MEP_{range} ($[I]^-$), a water-miscible anion having medium polarisability and a high MEP_{range} ($[MeSO_4]^-$), and a hydrophobic anion showing high polarisability and a high MEP_{range} ($[NTf_2]^-$). To further cover for low polarisability, but in conjunction with a high MEP_{range} on part of the cation, $[Me_3SO]^+$ was combined with $[Iodide]^-$, and to cover for a high polarisability and high MEP_{range} on part of the cation, $[P_{6,6,6,14}]^+$ was combined with the anion $[NTf_2]^-$, yielding a biphasic system. Sulfuric acid-based ionic liquids are inexpensive, easily recyclable and efficient catalysts in a range of chemical syntheses.[1] Especially with regards to deriving precursors from biomass or processing these precursors further to obtain pharmaceuticals or fine chemicals, these protic ILs are an interesting class to be investigated towards their impact on protein structural integrity and enzymatic activity. In order to investigate a range of emergent properties, the sulfuric acid derivative $[MeSO_4]^-$ was further combined with a water-immiscible cation of high polarisability and medium MEP_{range} $[N_{1,1,8,8}]^+$, yielding a second bi-phasic system, as well as with a charge-dispersed imidazolium cation $[DiMIM]^+$. This latter cation shows similar low polarisability and MEP_{range} as $[Me_3S]^+$ and was chosen to interrogate the influence of the structure by comparing the tetrahedral structure of $[Me_3S]^+$ to the planar structure of $[DiMIM]^+$. A second sulfuric acid derivate, which was additionally ether-functionalised, $[MeOEtSO_4]^-$, was consequently combined with $[BMIM]^+$ introducing a medium polarisability and a medium MEP_{range} on the cation and a high polarisability and high MEP_{range} for comparison with their shorter-chained counter-parts.

Hydroxyl-functionalised ILs were of specific interest since the hydroxyl group can act as an additional hydrogen bond donor and acceptor, while carboxylate groups can only act as hydrogen acceptors. [2] This property makes them interesting to investigate, especially with regards to halophilic protein surfaces and the interactions with these.

$[Choline]^+$ is nontoxic and renewable. It possesses a low polarisability despite its polar hydroxyl group, which is distanced from the not densely charge populated central nitrogen. Its non-hydroxyl counterpart $[N_{4,1,1,1}]^+$, which has similar low polarisability and similar MEP_{range}, was consequently chosen and paired with the medium polarisability and high MEP_{range} anion, $[(MeO)_2OPO]^-$, with the aim of investigating differences in protein surface interactions. The polarity of hydroxylated cations strongly depends on their respective counter-anion, while their non-hydroxylated versions do not.[3] $[Choline]^+$ was hence paired with the monatomic anion $[Cl]^-$ and the highly polarisable anion $[Bitartrate]^-$. The latter contains two dense charged areas located at the carboxylate groups, in addition to three hydroxyl groups and therefore possess also a large MEP_{range}. By comparison, the other hydroxyl-functionalised cation, $[Tetrakis]^+$, contains four symmetrically distributed hydroxyl groups relatively close to the phosphorous charged centre, leading to a smaller MEP_{range} and the same low polarisability as found for $[Choline]^+$. This cation was likewise paired with the very low polarisable anion $[Cl]^-$. Finally, the non-hydroxyl-functionalised counterpart to $[Tetrakis]^+$, cation $[P_{4,4,4,4}]^+$, possessing a high polarisability and medium MEP_{range} was likewise paired with $[Cl]^-$.

Summary of applied ionic liquids

SI Table S1.

Name	Cation	Anion
Trimethylsulfonium Methylsulfate	$[Me_3S]^+$	$[MeSO_4]^-$
Tetrakis(hydroxymethyl)-phosphonium Chloride	$[Tetrakis]^+$	$[Cl]^-$
2-Hydroxy-N,N,N-trimethylethan-1-aminium Chloride	$[Choline]^+$	$[Cl]^-$
2-Hydroxy-N,N,N-trimethylethan-1-aminium 3-Carboxy-2,3-dihydroxypropanoate	$[Choline]^+$	$[Bitartrate]^-$
1,3-Dimethyl-1H-imidazol-3-ium Methylsulfate	$[DiMIM]^+$	$[MeSO_4]^-$
Trimethylsulfonium Bis((trifluoromethyl)sulfonyl)-amide	$[Me_3S]^+$	$[NTf_2]^-$
3-Butyl-1-methyl-1H-imidazol-3-ium 2-Methoxyethyl sulfate	$[BMIM]^+$	$[MeOEtSO_4]^-$
N,N-Dimethyl-N-octyloctan-1-aminium Methanesulfonate	$[N_{1,1,8,8}]^+$	$[MeSO_4]^-$
Tetrabutylphosphonium Chloride	$[P_{4,4,4,4}]^+$	$[Cl]^-$
N,N,N-Trimethylbutan-1-aminium Dimethyl phosphate	$[N_{4,1,1,1}]^+$	$[(MeO)_2OPO]^-$
Trihexyl(tetradecyl)phosphonium Bis((trifluoromethyl)sulfonyl)-amide	$[P_{6,6,6,14}]^+$	$[NTf_2]^-$
Trimethylsulfoxonium Iodide	$[Me_3SO]^+$	$[I]^-$
Trimethylsulfonium Iodide	$[Me_3S]^+$	$[I]^-$

Preparation of ionic liquid mixtures

Following aqueous ionic liquid mixtures were applied: **$[Me_3S]^+[MeSO_4]^-$** 0.025 M, 0.075 M, 0.15 M, 0.3 M and 0.75 M. Starting at a concentration of 0.15 M a pH change was observed and adjusted back to pH 10.0 using 5 M KOH. **$[Tetrakis]^+[Cl]^-$** 0.025 M, 0.15 M, and 0.75 M. A pH change was observed at all concentrations and adjusted accordingly. **$[Choline]^+[Cl]^-$** 0.025 M, 0.15 M, and 0.75 M. Concentration 0.75 M was mixed using 50 mM Glycine-KOH, pH 10.0 buffer, 2 M KCl. **$[Choline]^+[Bitartrate]^-$** 0.025 M and 0.15 M were mixed in 50 mM Glycine-KOH, pH 10.0 buffer, 1.5 M KCl, whereby concentration 0.15 M was pH adjusted. **$[DiMIM]^+[MeSO_4]^-$** 0.025 M, 0.15 M, and 0.75 M. **$[Me_3S]^+[NTf_2]^-$** 0.025 M, 0.15 M, and 0.75 M. The latter concentration formed an emulsion. **$[BMIM]^+[MeOEtSO_4]^-$** 0.025 M and 0.15 M. Concentration 0.15 M was pH adjusted. **$[N_{1,1,8,8}]^+[MeSO_4]^-$** 0.025 M and 0.15 M. The latter concentration formed an emulsion. The 0.15 M concentration was pH adjusted. **$[P_{4,4,4,4}]^+[Cl]^-$** 0.025 M formed an emulsion. **$[N_{4,1,1,1}]^+[(MeO)_2OPO]^-$** 0.025 M, 0.15 M, and 0.75 M. The 0.75 M concentration was mixed in 50 mM Glycine-KOH, pH 10.0 buffer, 1 M KCl. **$[P_{6,6,6,14}]^+[NTf2]^-$** 0.025 M, 0.075 M, 0.15 M, 0.3 M and 0.75 M formed an emulsion. **$[Me_3SO]^+[I]^-$** 0.025 M and 0.15 M. **$[Me_3S]^+[I]^-$** 0.025 M, 0.075 M, 0.15 M, 0.3 M and 0.75 M.

Molecular electrostatic potentials for the cations considered

SI Table S2. The molecular electrostatic potential (MEP) for each of the cations of the ionic liquids used in this study. MEP colour range is represented on a blue-green-red colour scale ranging from 0 to 80 kcal mol^{-1} mapped onto the molecular electronic isodensity surface (isovalues ~ 0.02 e Å^{-3}).

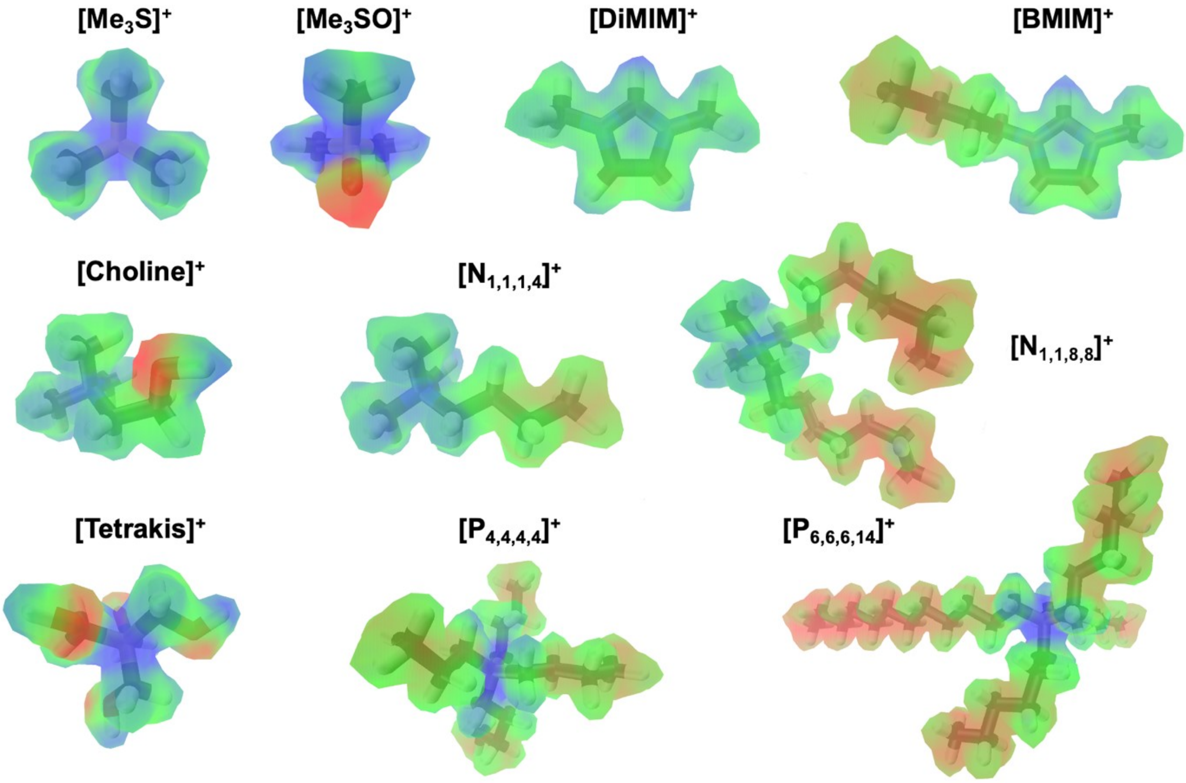

Molecular electrostatic potentials for the anions considered

SI Table S3. The molecular electrostatic potential (MEP) for each of the anions of the ionic liquids used in this study. MEP colour range is represented on a blue-green-red colour scale ranging from -100 to -20 kcal mol^{-1} mapped onto the molecular electronic isodensity surface (isovalues ~ 0.02 e Å^{-3}).

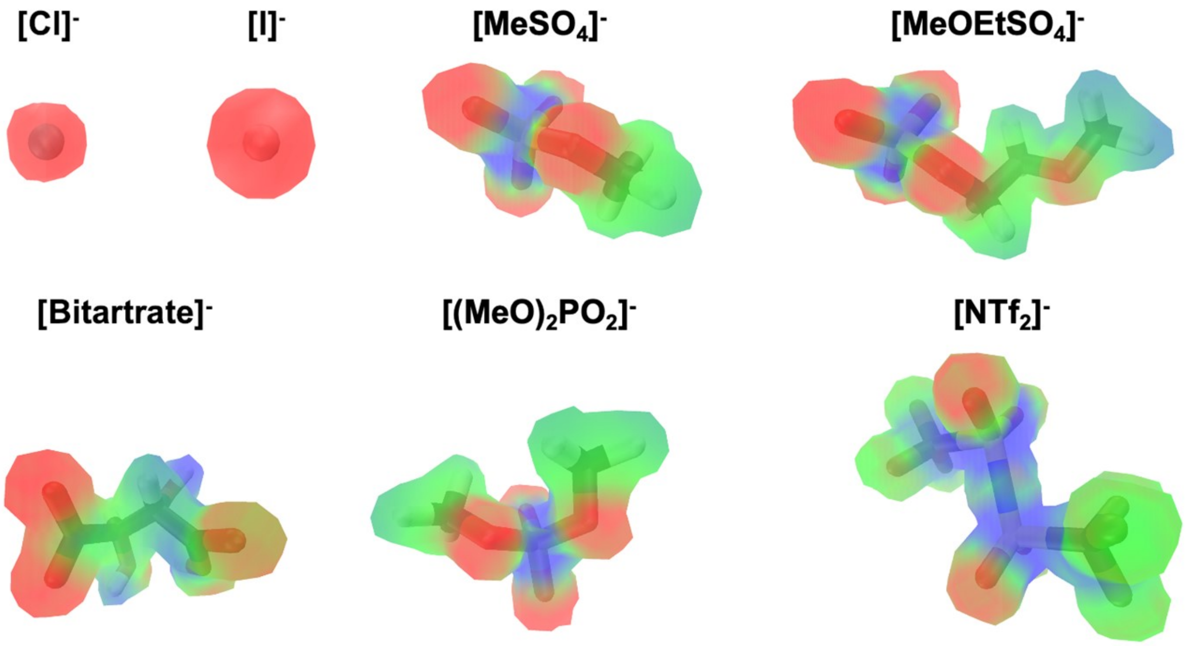

Relative activity of HvADH2 in aqueous ionic liquid mixtures

SI Figure S1. Ionic liquid solvent effect on the activity of *Hv*ADH2 in buffer (blue) and in aqueous ionic liquid mixtures (orange-hues) for ionic liquid systems $[Me_3S]^+[MeSO_4]^-$, $[P_{6,6,6,14}]^+[NTf2]^-$ and $[Me_3S]^+[I]^-$ (triplicates, error bars indicate standard deviations). While activity in $[Me_3S]^+[I]^-$ declines linearly with increasing concentration of IL, activity in $[Me_3S]^+[MeSO_4]^-$ and $[P_{6,6,6,14}]^+[NTf2]^-$ does not follow a linear trend. For both mixtures an activity optimum is observed at 300 mM IL.

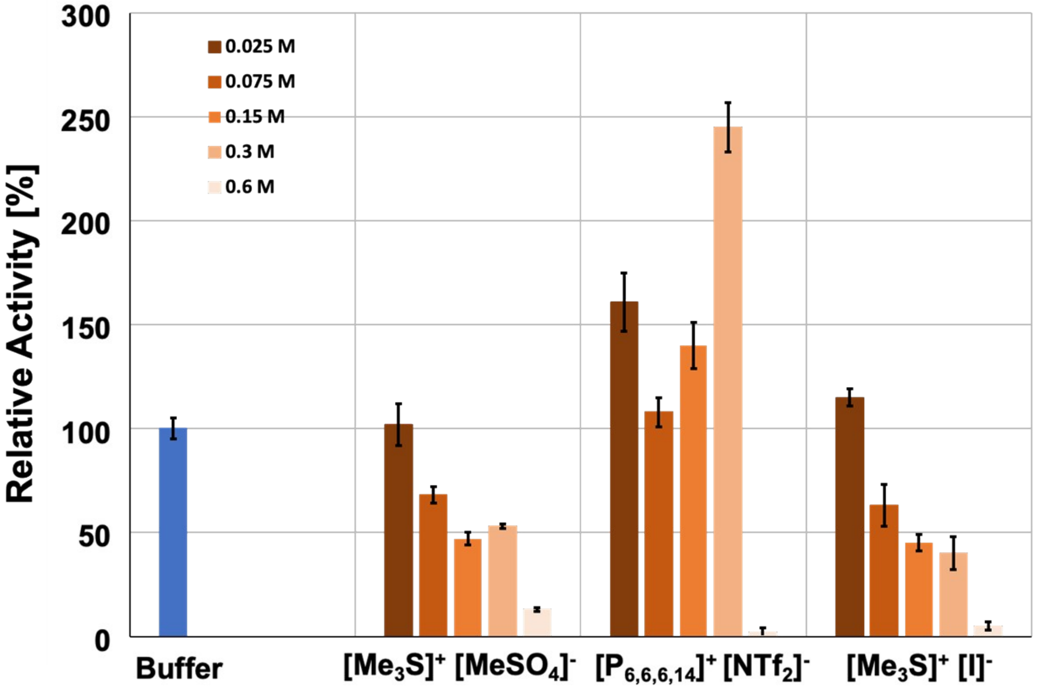

RMSD of four monomers

SI Figure S2. Ionic liquid solvent effect on the RMSD of *Hv*ADH2 monomers A (black), B (blue), C (green) and D (red) in buffer and in aqueous ionic liquid mixtures. All systems converge over the course of the trajectories, with exception of monomer C in the aqueous mixture containing $[BMIM]^+$ $[MeOEtSO_4]^+$ and confirm that simulations have been run long enough. Aggregates of IL ions on the surface Monomer C are observed in trajectories of $[BMIM]^+$ $[MeOEtSO_4]^+$ and explain why there is less convergence. Overall, RMSDs demonstrate that trajectories of monomers converge similarly, staying within a range of ~ 0.8 Å for the native system, 4 M KCl, confirming reproducibility. Despite different stochastic interactions with IL ions the majority of monomers in aqueous IL systems show comparable RMSDs between monomers of the same system too.

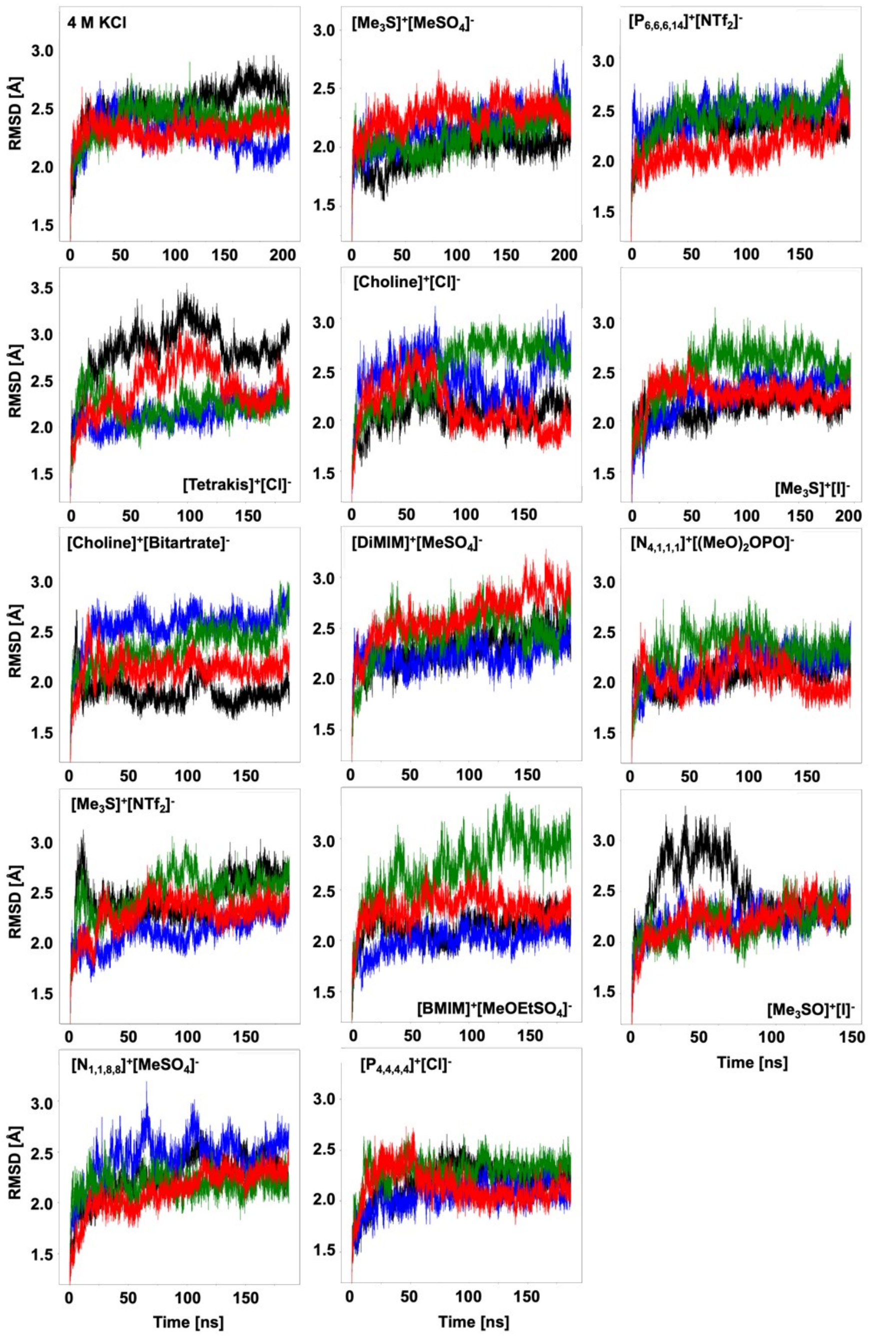

Standard deviation of RMSF values of four monomers

SI Figure S3. Ionic liquid solvent effects examined for native HvADH2 and in ionic liquid mixtures, throughout MD simulation (150 - 210 ns). The sum of the RMSF standard deviation values over the whole sequence was: 8.26 nm for 4 M KCl; 17.97 nm for $[Me_3S]^+[MeSO_4]^-$; 9.25 nm for $[Tetrakis]^+ [Cl]^-$, 9.64 nm for $[Choline]^+ [Cl]^-$, 10.85 nm for $[Choline]^+ [Bitartrate]^-$, 13.87 nm for $[DiMIM]^+ [MeSO_4]^-$, 17.72 nm for $[Me_3S]^+ [NTf_2]^-$, 20.68 nm for $[BMIM]^+ [MeOEtSO_4]^-$, 17.60 nm for $[N_{1,1,8,8}]^+ [MeSO_4]^-$, 10.00 nm for $[P_{4,4,4,4}]^+ [Cl]^-$, 12.00 nm for $[N_{4,1,1,1}]^+ [(MeO)_2OPO]^-$, 11.66 nm for $[P_{6,6,6,14}]^+ [NTf_2]^-$, 8.51 nm for $[Me_3SO]^+ [I]^-$ and 10.82 nm for $[Me_3S]^+ [I]^-$.

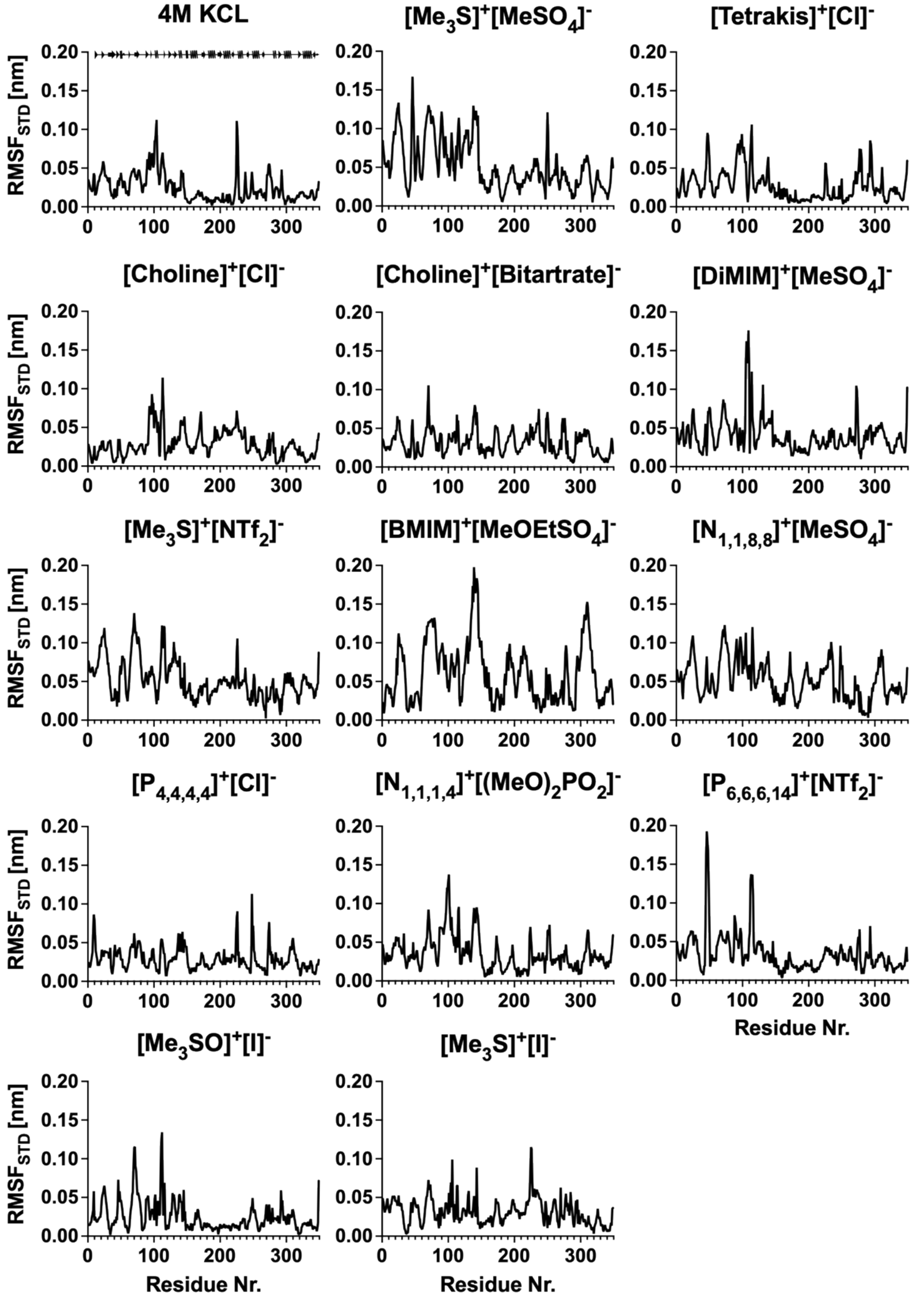

Bond distance of active residues in monomers A and C of the native system

SI Figure S4. Distance between residues of the catalytic triad Ser_{40}, His_{59} and Asp_{155} and zinc-coordinating Cys_{38} is plotted over the course of the simulation of the native system for monomers A and C. Monomers plotted here are in addition to monomers B and D plotted in **SI Figure 5** of the main manuscript. Comparison between monomers clarifies slight differences observed in RMSDs in **SI Figure S2**. While monomer B and monomer D remain static over the course of the trajectories, because monomer B has no cofactor bound and monomer D stays bound to NAD^+ over the whole course of the trajectory, monomers A and C expel NAD^+. In monomer A the cofactor is guided out of the binding pocket between 10 and 20 ns, while in monomer C NAD^+ is released from the binding pocket at around 100 ns. This reflects well in the diverging distance between residues Asp_{155} to His_{59} between monomers. Distance between Cys_{38} and His_{59} stays remarkably constant for all monomers and confirms the overall stability of and reproducibility between monomers.

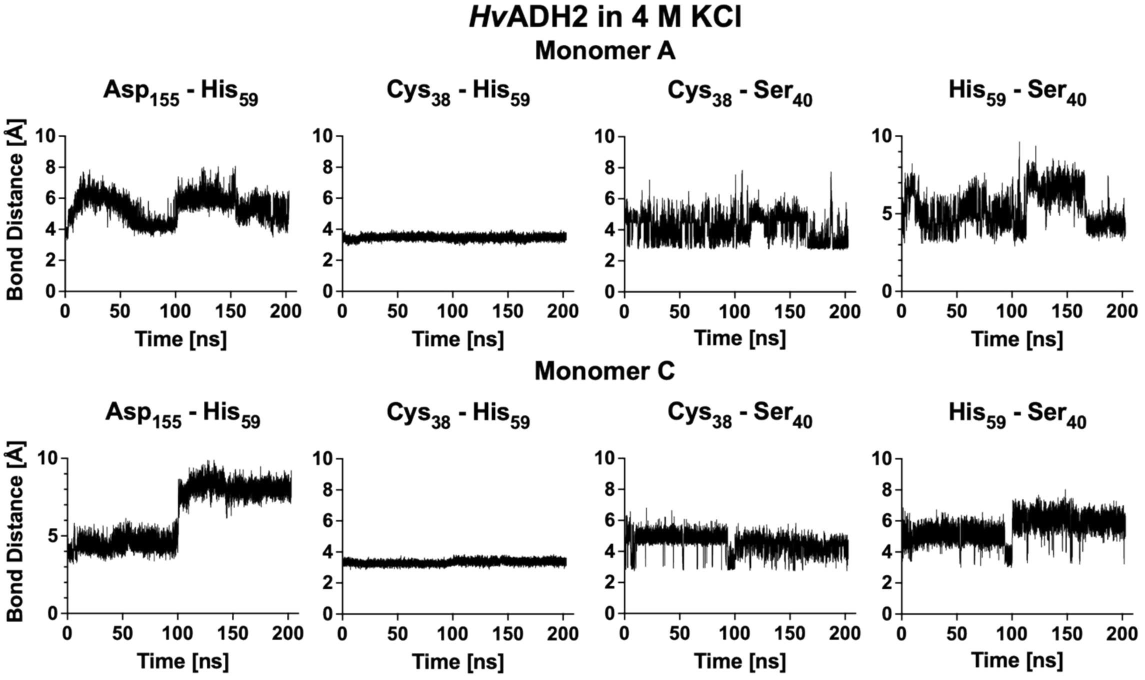

Bond distance of active residues in monomers B, C of the native system in comparison to aqueous ionic liquid systems

SI Figure S5. Distance between residues of the catalytic triad Ser_{40}, His_{59} and Asp_{155} and zinc-coordinating Cys_{38} is plotted over the course of the simulation of the native system (two top rows) and of selected residues of the catalytic triad of ionic liquid containing systems of monomer B (black) and monomer D (grey).

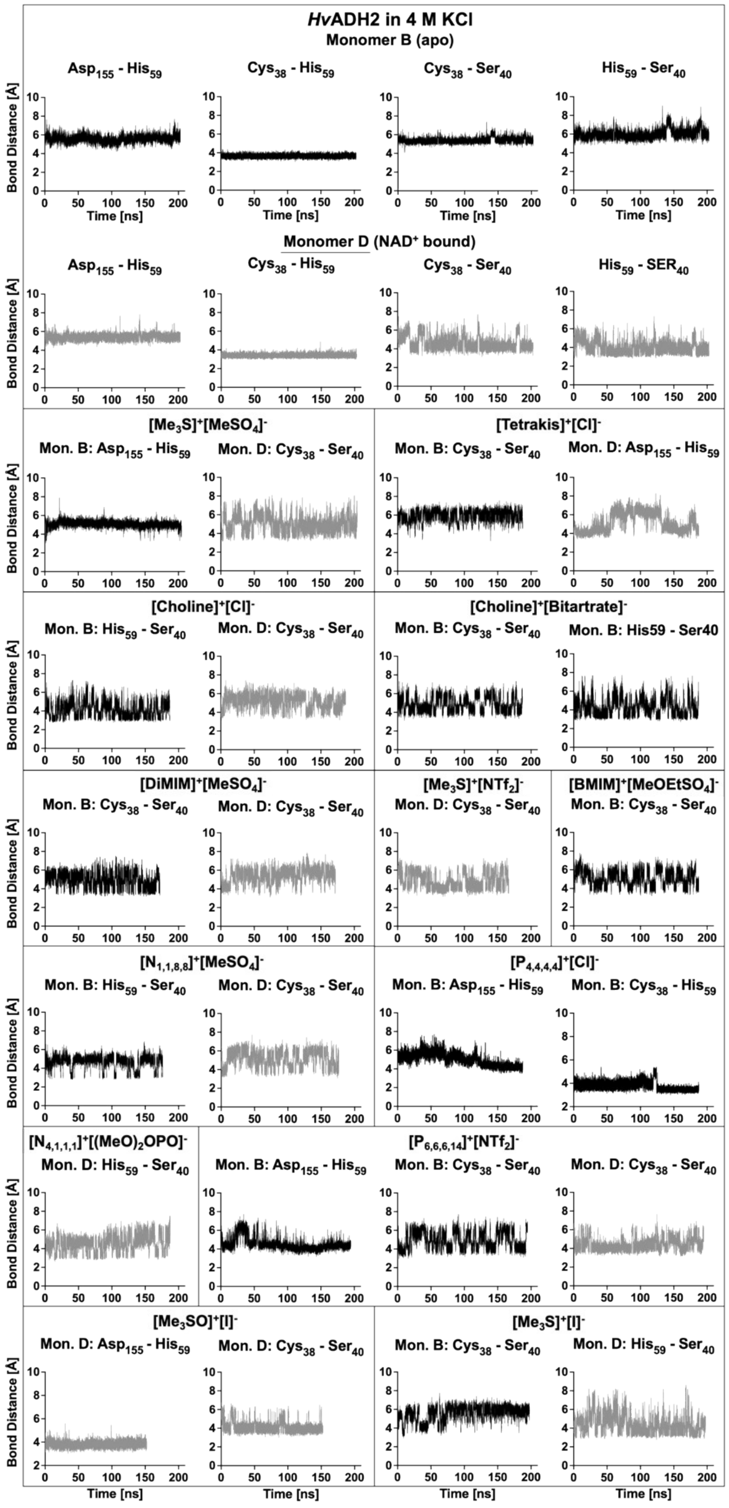

Conformation of gating residues Trp_{43} and His_{273} after MD run within the native system

SI Figure S6. (A) Active-conformation-gating residues within monomer A towards the end of the trajectory (~ 180 ns) and **(B)** same residues within monomer D at the same time step. While NAD^+ stays in proximity to the catalytic zinc in monomer D, it gets removed to an outer surface crevice in monomer A, and gate-keeping residues assume a π-stacking conformation.

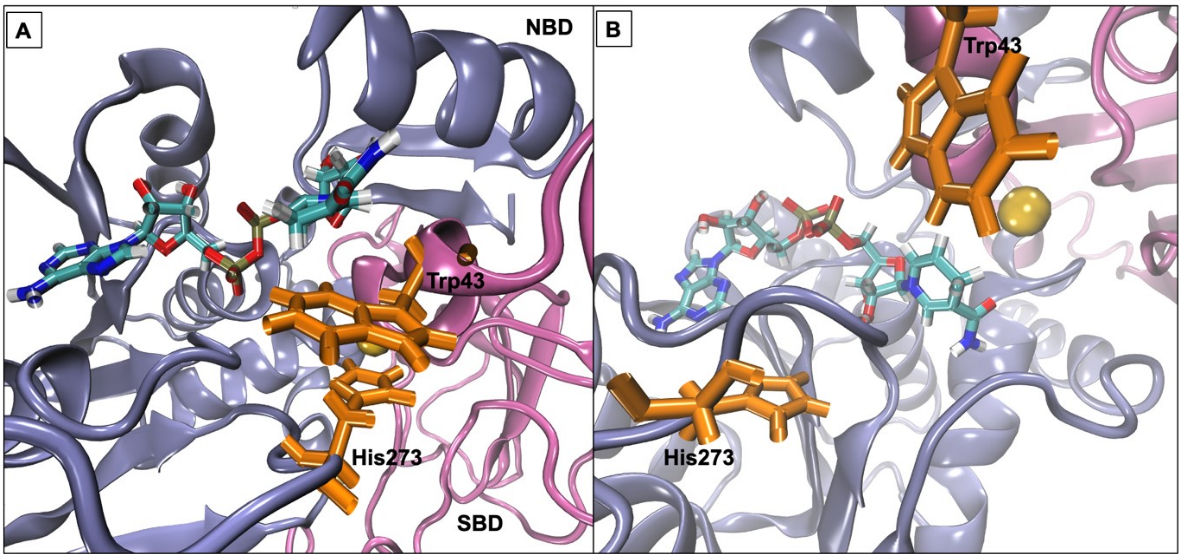

Distance between gating residues Trp_{43} and His_{273} over the course of the trajectory in the native system and ionic liquids

SI Figure S7. The change of bond distance between vanguard residues Trp_{43} and His_{273} in monomers A and D in the native system as well as in ILs is plotted with VMD over the course of trajectories. Within the native system gating residues in monomer A jointly coordinate NAD^+ out of the binding pocket until 20 ns of the trajectory. A rearrangement of the residues takes place between 20 to 60 ns of the trajectory as can be seen from an increase in distance between residues. Thereafter residues close in on each other until the π-stacking conformation is achieved between Trp_{43} and His_{273} at 100 ns. Although the distance between gating residues varies between 100 ns and the end of the trajectory at 200 ns, the π-stacking interaction persists and stays below 6 Å past 130 ns. Distance between gating residues in monomer D varies between 10 and 15 Å in the native system and NAD^+ stays coordinated to the catalytic zinc over the course of the whole trajectory.

The following IL systems prevent expulsion of NAD^+ from monomer A: $[Choline]^+[Cl]^-$, $[Me_3S]^+[MeSO_4]^-$, $[BMIM]^+[MeOEtSO_4]^-$, $[N_{1,1,8,8}]^+[MeSO_4]^-$, $[P_{4,4,4,4}]^+[Cl]^-$, $[N_{1,1,1,4}]^+[(MeO)_2OPO]^-$, $[Me_3SO]^+[I]^-$ and $[Me_3S]^+[I]^-$. While gating residues of monomer A in systems $[BMIM]^+[MeOEtSO_4]^-$, $[N_{1,1,8,8}]^+[MeSO_4]^-$, $[P_{4,4,4,4}]^+[Cl]^-$ and $[N_{1,1,1,4}]^+[(MeO)_2OPO]^-$ assume a similar distance average as in the native monomer D, gating residues in systems $[Choline]^+[Cl]^-$, $[Me_3S]^+[MeSO_4]^-$and $[Me_3SO]^+[I]^-$ and $[Me_3S]^+[I]^-$ remain in close distance, more similar to native monomer A.

In the following IL systems NAD^+ gets pulled from the catalytic zinc vicinity of monomer D: $[Choline]^+[Cl]^-$, $[Tetrakis]^+[Cl]^-$, $[DiMIM]^+[MeSO_4]^-$, $[BMIM]^+[MeOEtSO_4]^-$ and $[N_{1,1,1,4}]^+[(MeO)_2OPO]^-$. Only system $[Choline]^+[Cl]^-$ shows at the end of the trajectory a similar distance between gating residues as found in native monomer A. The other systems, $[Tetrakis]^+[Cl]^-$, $[DiMIM]^+[MeSO_4]^-$, $[BMIM]^+[MeOEtSO_4]^-$ and $[N_{1,1,1,4}]^+[(MeO)_2OPO]^-$, are prevented from assuming the π-stacking interaction by IL ions.

The following systems show the same dissociation of NAD^+, or lack thereof, as in the native system: $[Choline]^+[Bitartrate]^-$, $[Me_3S]^+[NTf_2]^-$ and $[P_{6,6,6,14}]^+[NTf_2]^-$. Gating residues distance in these IL systems is overall similar to the native system. However, in systems $[Choline]^+[Bitartrate]^-$ and $[Me_3S]^+[NTf_2]^-$, the variation in distance is markedly more rigid compared to the native system.

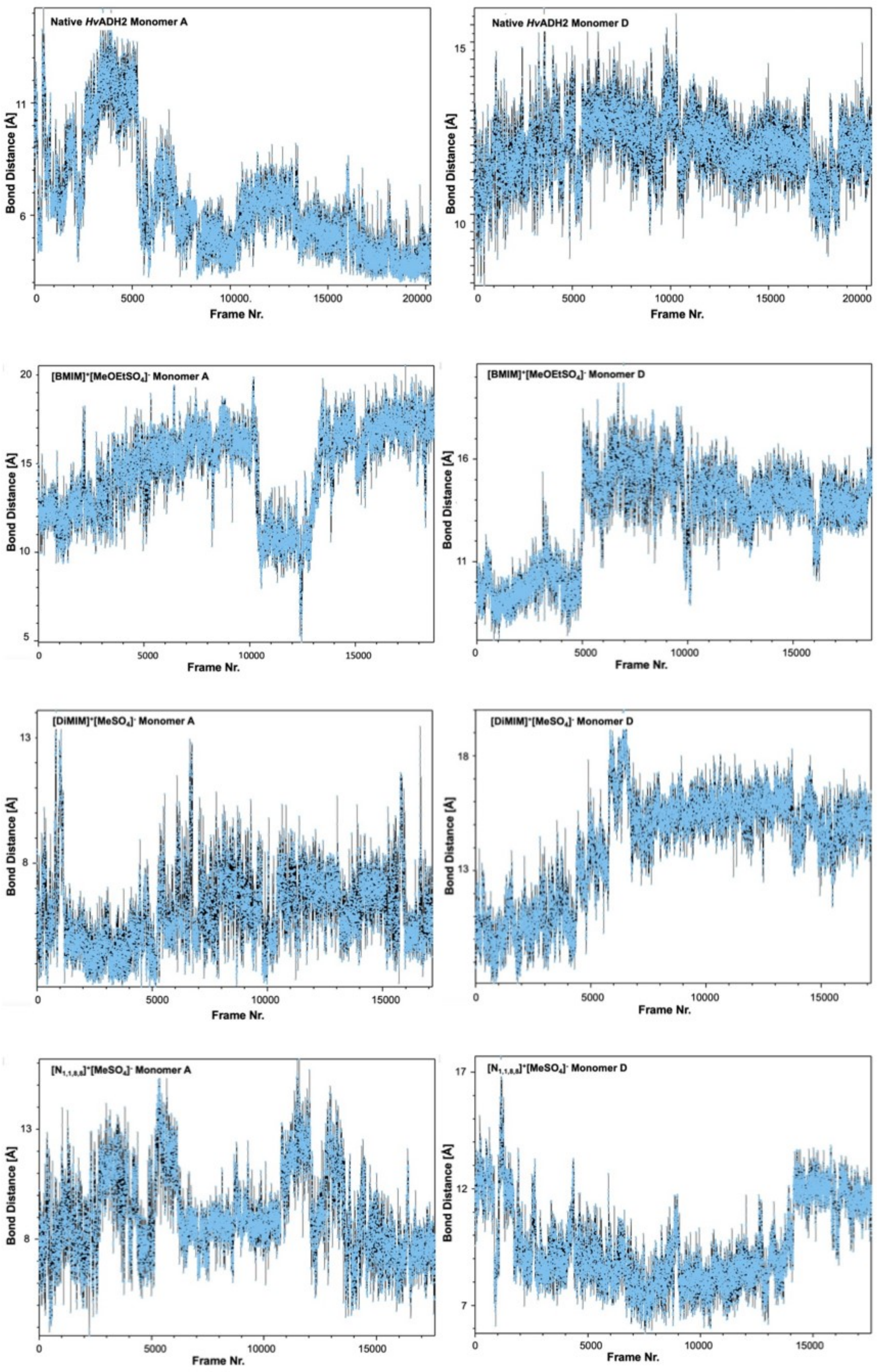
Native *Hv*ADH2 Monomer A
Native *Hv*ADH2 Monomer D
[BMIM]+[MeOEtSO4]- Monomer A
[BMIM]+[MeOEtSO4]- Monomer D
[DiMIM]+[MeSO4]- Monomer A
[DiMIM]+[MeSO4]- Monomer D
[N1,1,8,8]+[MeSO4]- Monomer A
[N1,1,8,8]+[MeSO4]- Monomer D
Bond Distance [Å]
Frame Nr.

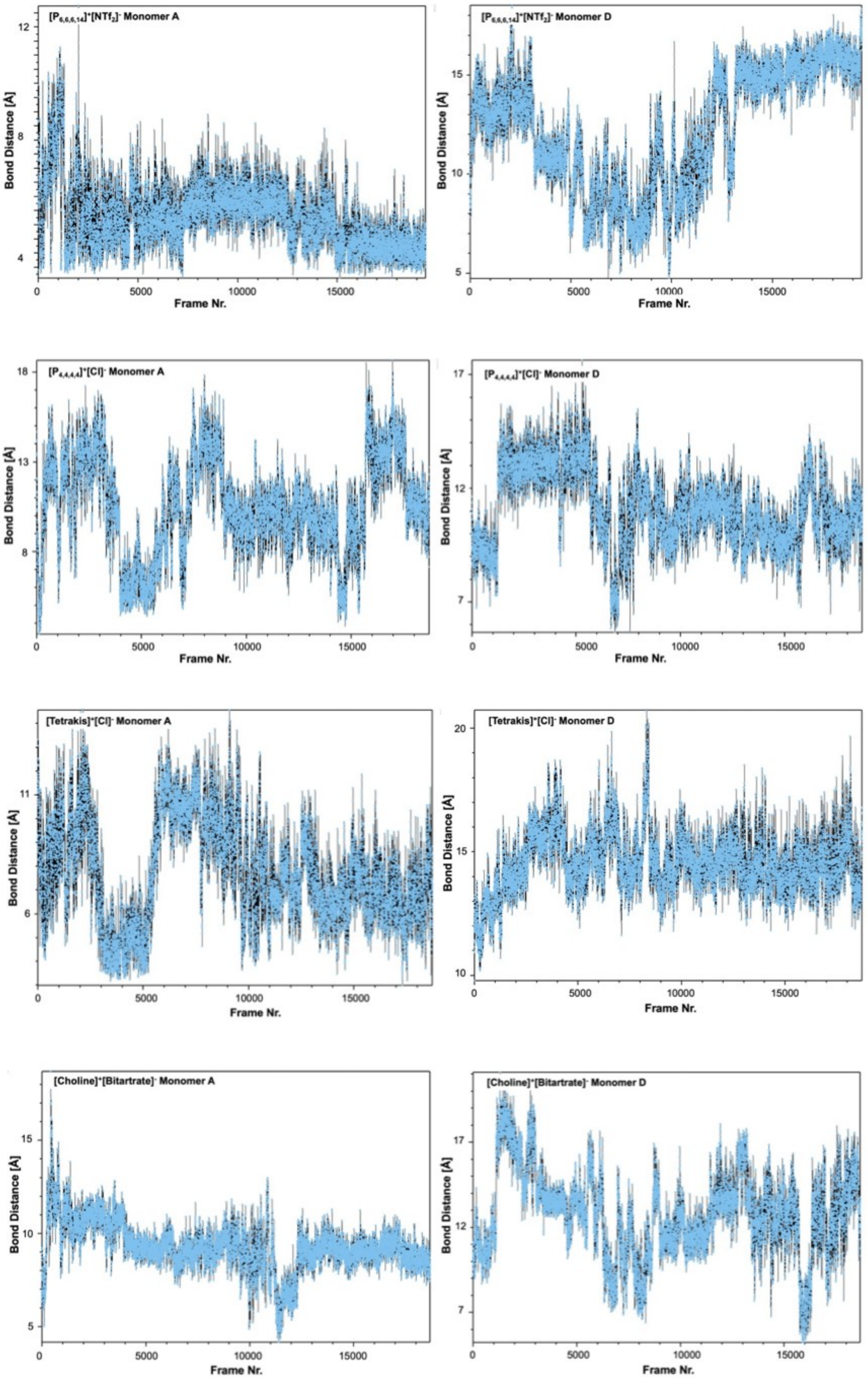
[P6,6,6,14]+[NTf2]- Monomer A
[P6,6,6,14]+[NTf2]- Monomer D
[P4,4,4,4]+[Cl]- Monomer A
[P4,4,4,4]+[Cl]- Monomer D
[Tetrakis]+[Cl]- Monomer A
[Tetrakis]+[Cl]- Monomer D
[Choline]+[Bitartrate]- Monomer A
[Choline]+[Bitartrate]- Monomer D
Bond Distance [Å]
Frame Nr.

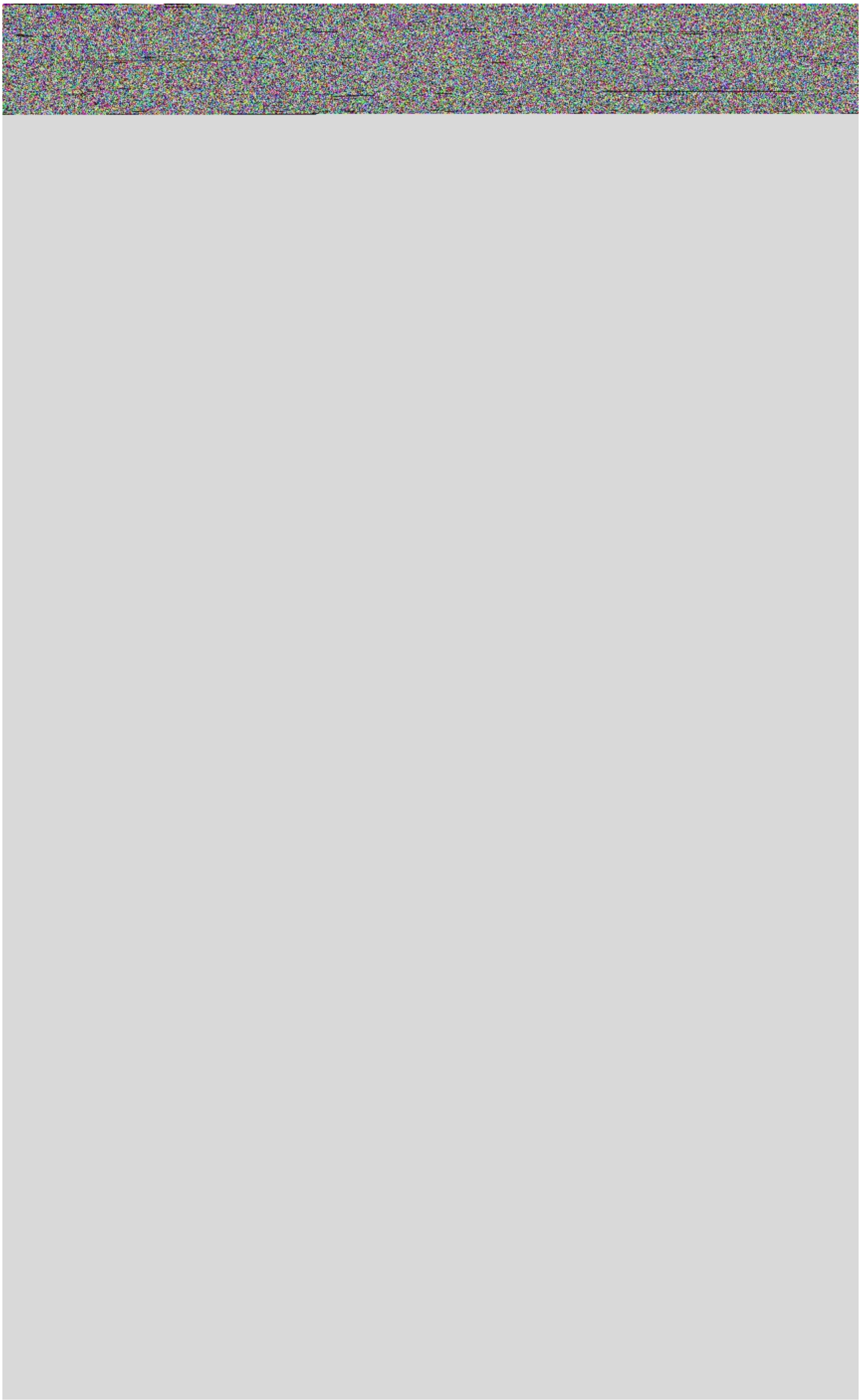

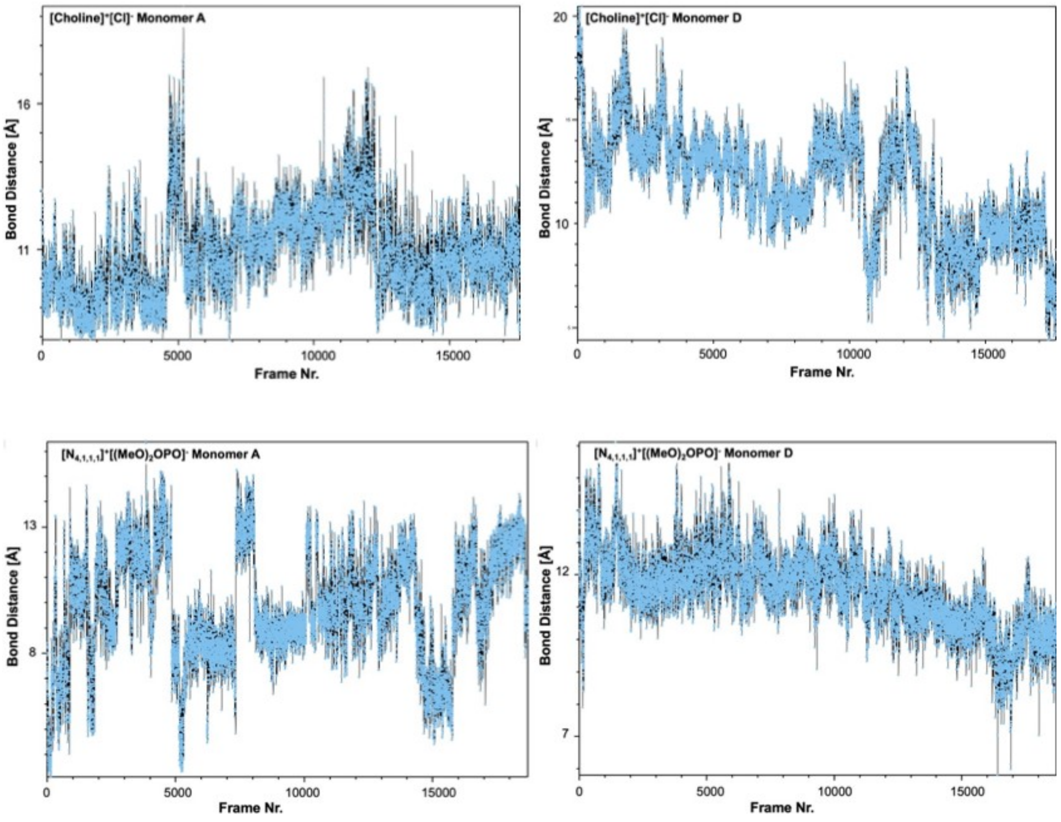
[Choline]+[Cl]- Monomer A
[Choline]+[Cl]- Monomer D
[N4,1,1,1]+[(MeO)2OPO]- Monomer A
[N4,1,1,1]+[(MeO)2OPO]- Monomer D
Bond Distance [Å]
Frame Nr.

Free Energy Landscapes and Secondary Structure Assignment Plots

SI Figure S8. Free Energy Landscapes of the native *Hv*ADH2 system and ionic liquid systems (Left) and Secondary structure plots (right) of HvADH2 in aqueous mixtures of ILs.

FELs of ILs [Choline]$^+$[Bitartrate]$^-$, [DiMIM]$^+$[MeSO$_4$]$^-$, [BMIM]$^+$[MeOEtSO$_4$]$^-$, [P$_{4,4,4,4}$]$^+$[Cl]$^-$, [P$_{6,6,6,14}$]$^+$[NTf$_2$]$^-$ and [Me$_3$SO]$^+$[I]$^-$ are characterised by narrow troughs and a steep descent towards the final minimum, visiting very few or no stabilising minima from which structures need to ascend first to be able to descend into the next minimum. This suggests a trapping of the enzyme in non-native states, since stabilising minima allow for a reconfiguration of folded structural elements before the structure with lowest energy can be achieved. This rearrangement of structure appears to be prevented by these IL ion combinations.

FELs of ILs [Me$_3$S]$^+$[MeSO$_4$]$^-$, [Tetrakis]$^+$[Cl]$^-$, [Choline]$^+$[Cl]$^-$, [Me$_3$S]$^+$[NTf$_2$]$^-$, [N$_{1,1,8,8}$]$^+$[MeSO$_4$]$^-$, [N$_{4,1,1,1}$]$^+$[(MeO)$_2$OPO]$^-$ and [Me$_3$S]$^+$[I]$^-$ show an increased number of local deep minima, which are mainly separated by low energy barriers, and some show no discernible descent with minima being more or less equal in energy. This suggests that the native structure of the enzyme is destabilised, since a clear energy preference for one conformation is prevented by these ILs.

Most similar to the FEL of the native system is the FEL of [Me$_3$S]$^+$[MeSO$_4$]$^-$, showing three local minima separated by comparably high energy barriers as found for the native system. Exceptional FELs are found for [Me$_3$SO]$^+$[I]$^-$ and [Tetrakis]$^+$[Cl]$^-$, where ions appear to de-stabilise enzyme structure to such an extent that exchange between lowest energy minima requires an ascent to the highest energy conformations.

$[Me_3S]^+ [MeSO_4]^-$

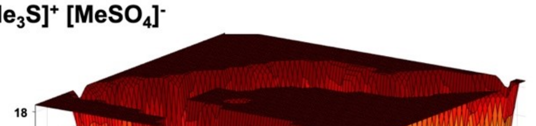

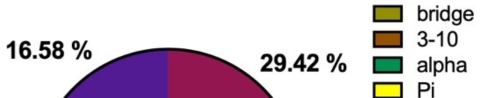

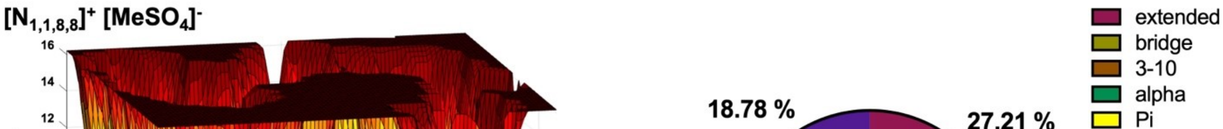
$[N_{1,1,8,8}]^+$ $[MeSO_4]^-$
16
14
12
18.78 %
27.21 %
extended
bridge
3-10
alpha
Pi

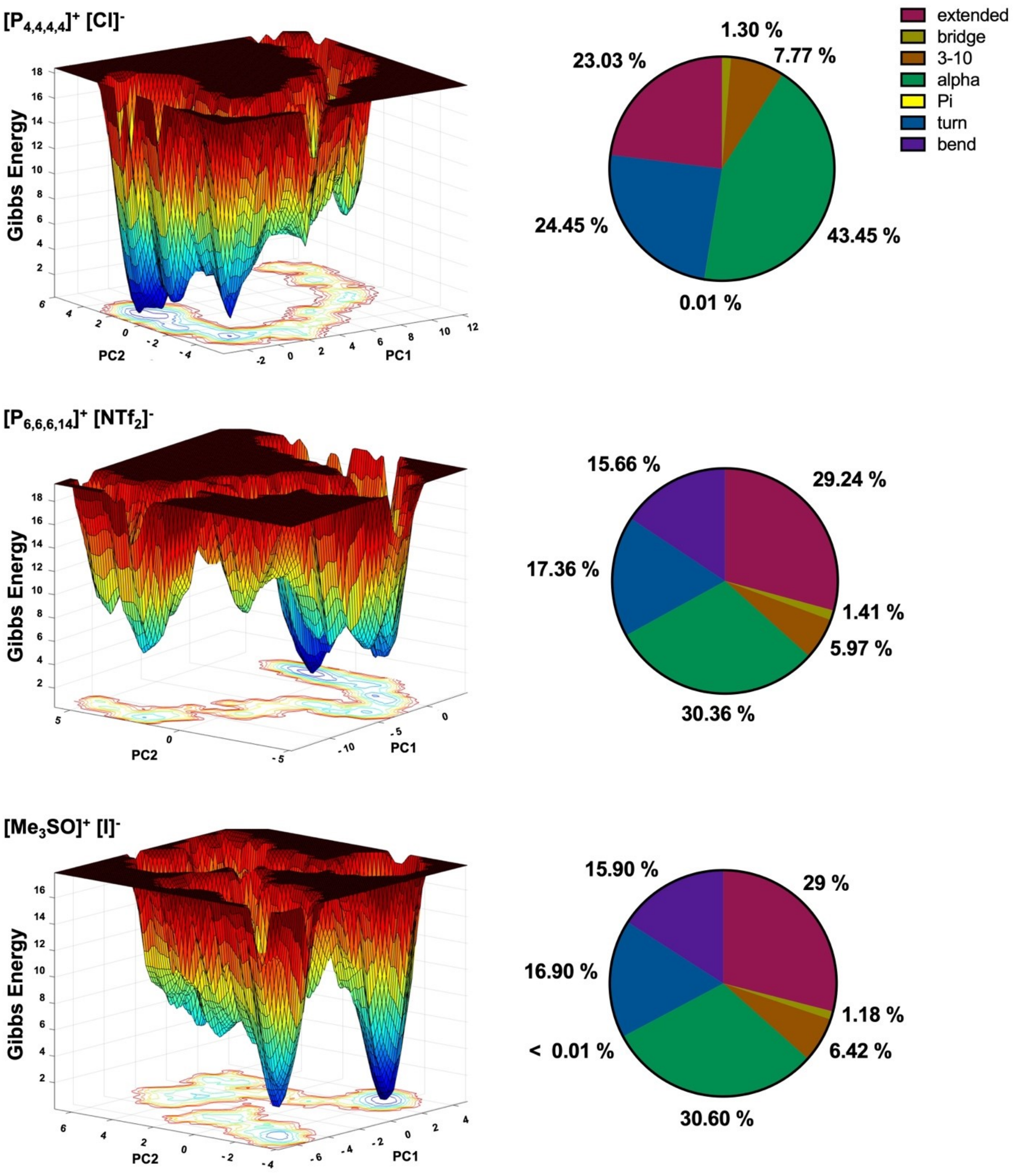

[P4,4,4,4]+ [Cl]-
Gibbs Energy
PC2
PC1
23.03 %
1.30 %
7.77 %
24.45 %
43.45 %
0.01 %
extended
bridge
3-10
alpha
Pi
turn
bend
[P6,6,6,14]+ [NTf2]-
Gibbs Energy
PC2
PC1
15.66 %
29.24 %
17.36 %
1.41 %
5.97 %
30.36 %
[Me3SO]+ [I]-
Gibbs Energy
PC2
PC1
15.90 %
29 %
16.90 %
1.18 %
< 0.01 %
6.42 %
30.60 %

Heatmap binding energies between IL ions and acidic residues

SI Table S4. Binding energies of IL cations and anions to acidic residues. Binding energies are influenced by the respective counter-ion in the system.

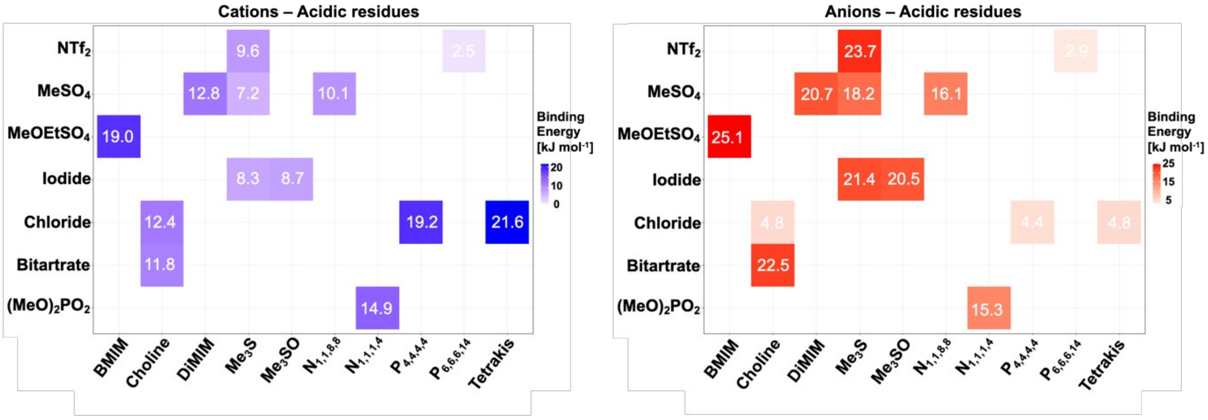

Radial Distribution Functions (RDFs) of ionic liquid ions around *Hv*ADH2

SI Figure S9. RDFs ($g_{(r)}$) of IL anions and cations around acidic, basic or all uncharged surface residues of *Hv*ADH2. All ionic liquids containing $[Cl]^-$, ($[Tetrakis]^+[Cl]^-$, $[Choline]^+[Cl]^-$ and $[P_{4,4,4,4}]^+[Cl]^-$), are the only systems in which the cation coordinates closer and more frequent to the protein surface than the anion. For all other systems the IL-anions appear to preferentially displace the IL-cations around the protein surface. Comparison of the coordination of $[Tetrakis]^+$ and $[P_{4,4,4,4}]^+$ to *Hv*ADH2 demonstrates the difference between the presence and absence of hydroxyl-groups. While the nonpolar alkyl groups of $[P_{4,4,4,4}]^+$ are non-preferentially interacting with the protein surface, the polar hydroxyl-groups on $[Tetrakis]^+$ intrude below the hydrogen bond network at 2 Å around negatively charged residues. The first coordination shell of $[Tetrakis]^+$ is found peaking at around 1.8 Å. For the other two hydroxyl-functionalised ions $[Choline]^+$ and $[Bitartrate]^-$ a similar profile is observed. However, while $[Tetrakis]^+$ and $[Choline]^+$ coordinate preferentially to negatively charged residues, [Bitartrate]- intrudes similarly close at 1.9 Å into the H-bond network around positively charged residues. Contrastingly to $[Cl]^-$ IL-anions $[I]^-$ monatomic ions coordinate closely and abundantly to the protein surface. In both systems, $[Me_3S]^+$ $[I]^-$ and $[Me_3SO]^+$ $[I]^-$, $[I]^-$ coordinates more frequently non-preferentially to the protein surface than to charged residues. Unexpected is the difference in coordination of $[I]^-$ to positively charged residues between these two systems. The peak of the first coordination shell in the RDF profile of $[I]^-$ paired with $[Me_3SO]^+$ is decreased by almost one half when compared to $[I]^-$ paired with $[Me_3S]$+. An explanation could be a preferential $[I]^-$ interaction with the additional, polar and sterically different sulfoxide group over the apolar methyl groups, which results in a screening of ionic interactions with positively charged residues for the system $[Me_3SO]^+[I]^-$. That the interaction between ions is important in their relation to the protein surface is further observed for the comparison of RDF profiles of $[Me_3S]^+$ around *Hv*ADH2 when paired with either $[MeSO_4]^-$, $[NTf_2]^-$ or $[I]^-$. Least frequent interaction of $[Me_3S]^+$ with the protein is observed for $[MeSO_4]^-$. Within this system $[MeSO_4]^-$ itself is found least coordinating of all IL-anions to the protein surface, with the exception of $[Cl]^-$ and $[P_{6,6,6,14}]^-$. By comparison, $g_{(r)}$ values are increased for $[Me_3S]^+$ around *Hv*ADH2 when paired with $[I]^-$ and $[NTf_2]^-$, increasing in this order. However, RDF profiles look highly similar. For $[MeSO_4]^-$ paired with either $[Me_3S]^+$ or $[DiMIM]^+$ $g_{(r)}$ values are substantially increased for $[MeSO_4]^-$ paired with $[DiMIM]^+$, highlighting the importance of counter-ion interaction and influence on protein surface. The ether group present in $[MeOEtSO4]^-$ leads to a direct interaction with the protein surface below the H-bond network at 1 Å. This intrusion is non-preferential to charged residues and leads to multiple distinct coordination distances within 1- 2.5 Å populated with increasing probability following increasing distance from the surface. A second main coordination shell is present between 3- 10 Å. Preferential coordination to both acidic and basic residues is observed for this anion with one distinct coordination shell peaking at 1.5 Å. However, ionic interaction between $[MeOEtSO_4]^-$ and basic residues is prevalent as indicated by almost double the peak height compared to its coordination to acidic residues.

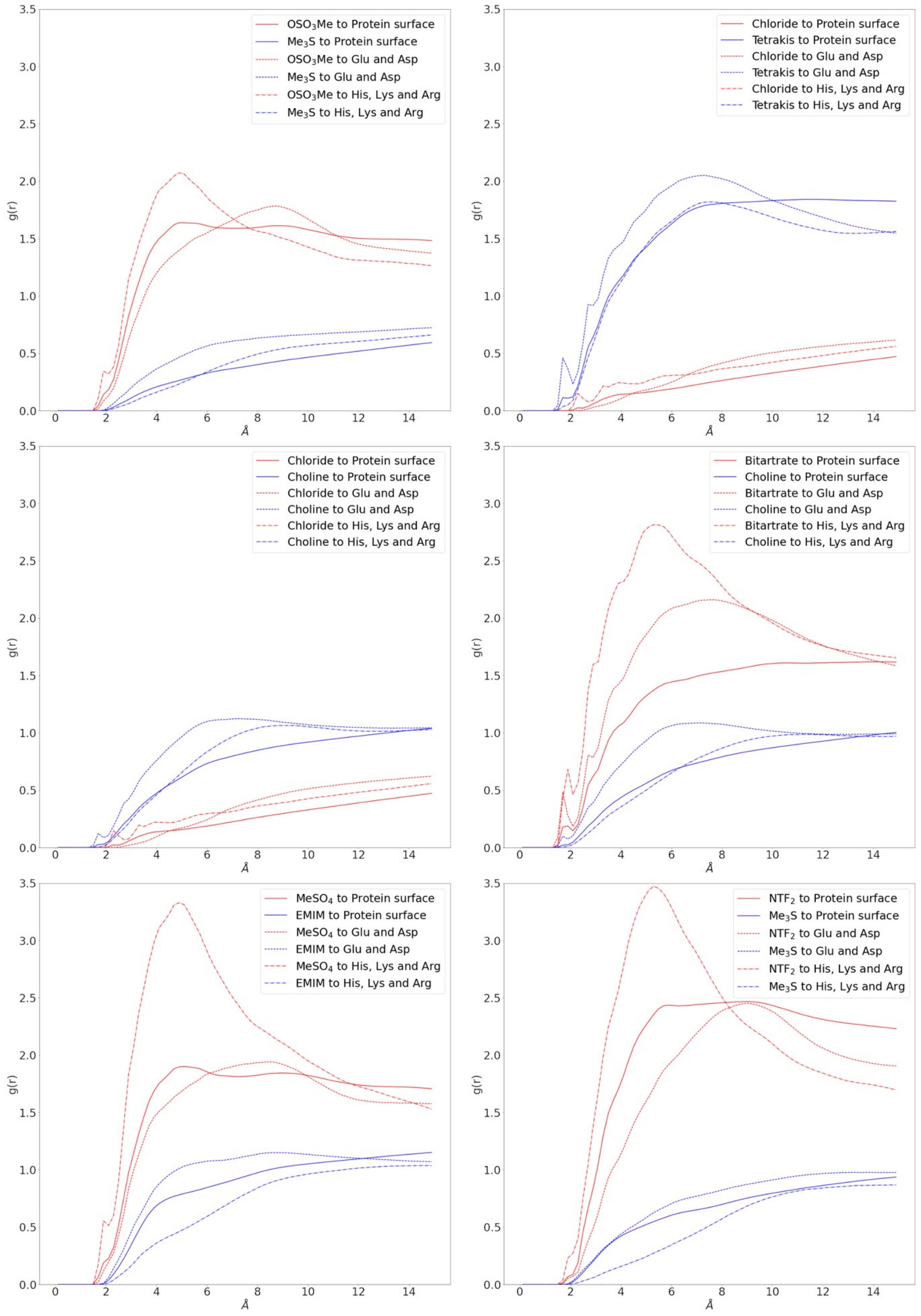
OSO_3Me to Protein surface
Me_3S to Protein surface
OSO_3Me to Glu and Asp
Me_3S to Glu and Asp
OSO_3Me to His, Lys and Arg
Me_3S to His, Lys and Arg
Chloride to Protein surface
Tetrakis to Protein surface
Chloride to Glu and Asp
Tetrakis to Glu and Asp
Chloride to His, Lys and Arg
Tetrakis to His, Lys and Arg
Chloride to Protein surface
Choline to Protein surface
Chloride to Glu and Asp
Choline to Glu and Asp
Chloride to His, Lys and Arg
Choline to His, Lys and Arg
Bitartrate to Protein surface
Choline to Protein surface
Bitartrate to Glu and Asp
Choline to Glu and Asp
Bitartrate to His, Lys and Arg
Choline to His, Lys and Arg
$MeSO_4$ to Protein surface
EMIM to Protein surface
$MeSO_4$ to Glu and Asp
EMIM to Glu and Asp
$MeSO_4$ to His, Lys and Arg
EMIM to His, Lys and Arg
NTF_2 to Protein surface
Me_3S to Protein surface
NTF_2 to Glu and Asp
Me_3S to Glu and Asp
NTF_2 to His, Lys and Arg
Me_3S to His, Lys and Arg
g(r)
Å

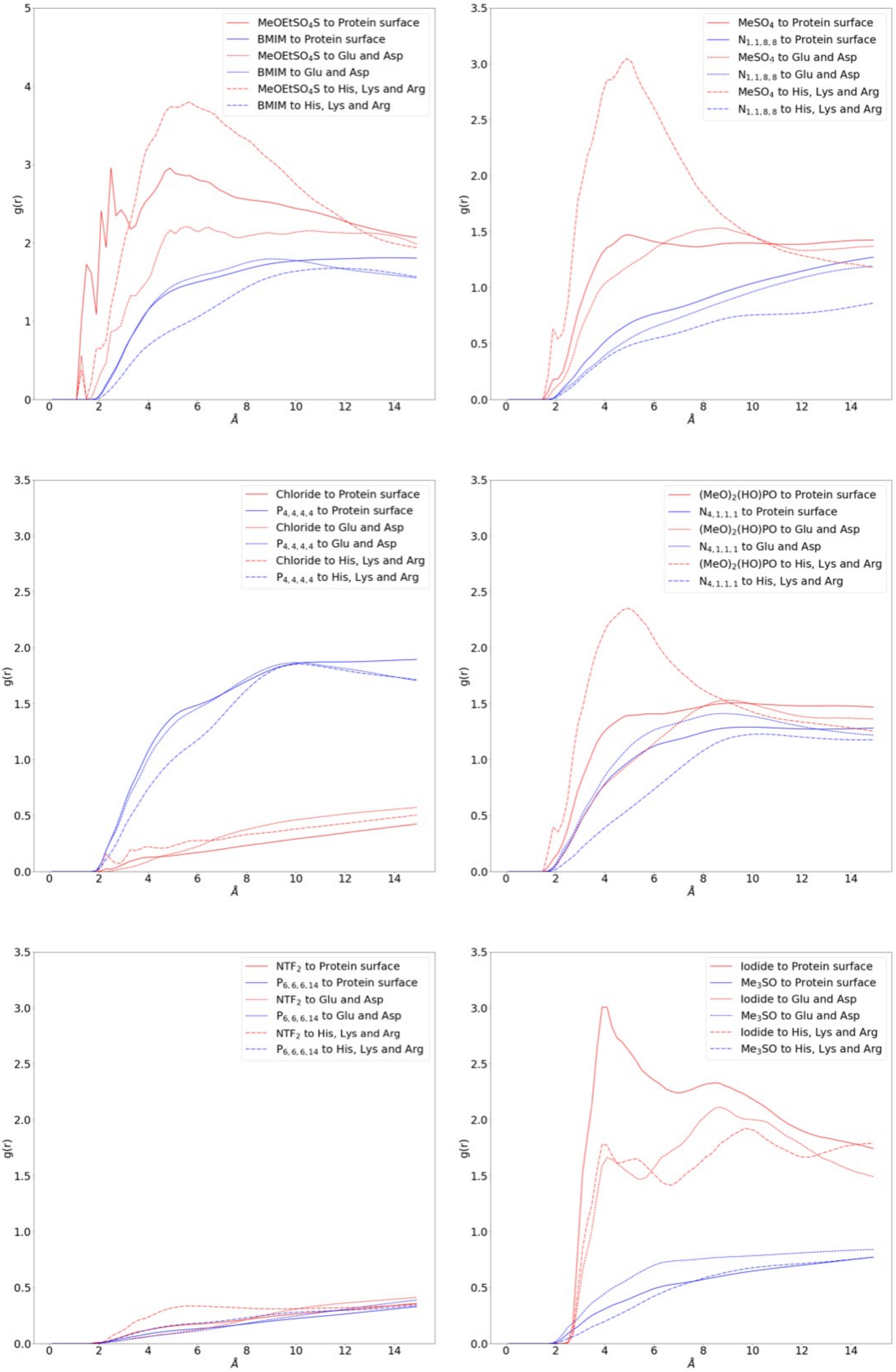

$MeOEtSO_4S$ to Protein surface
BMIM to Protein surface
$MeOEtSO_4S$ to Glu and Asp
BMIM to Glu and Asp
$MeOEtSO_4S$ to His, Lys and Arg
BMIM to His, Lys and Arg
$MeSO_4$ to Protein surface
$N_{1,1,8,8}$ to Protein surface
$MeSO_4$ to Glu and Asp
$N_{1,1,8,8}$ to Glu and Asp
$MeSO_4$ to His, Lys and Arg
$N_{1,1,8,8}$ to His, Lys and Arg
Chloride to Protein surface
$P_{4,4,4,4}$ to Protein surface
Chloride to Glu and Asp
$P_{4,4,4,4}$ to Glu and Asp
Chloride to His, Lys and Arg
$P_{4,4,4,4}$ to His, Lys and Arg
$(MeO)_2(HO)PO$ to Protein surface
$N_{4,1,1,1}$ to Protein surface
$(MeO)_2(HO)PO$ to Glu and Asp
$N_{4,1,1,1}$ to Glu and Asp
$(MeO)_2(HO)PO$ to His, Lys and Arg
$N_{4,1,1,1}$ to His, Lys and Arg
NTF_2 to Protein surface
$P_{6,6,6,14}$ to Protein surface
NTF_2 to Glu and Asp
$P_{6,6,6,14}$ to Glu and Asp
NTF_2 to His, Lys and Arg
$P_{6,6,6,14}$ to His, Lys and Arg
Iodide to Protein surface
Me_3SO to Protein surface
Iodide to Glu and Asp
Me_3SO to Glu and Asp
Iodide to His, Lys and Arg
Me_3SO to His, Lys and Arg
g(r)
Å

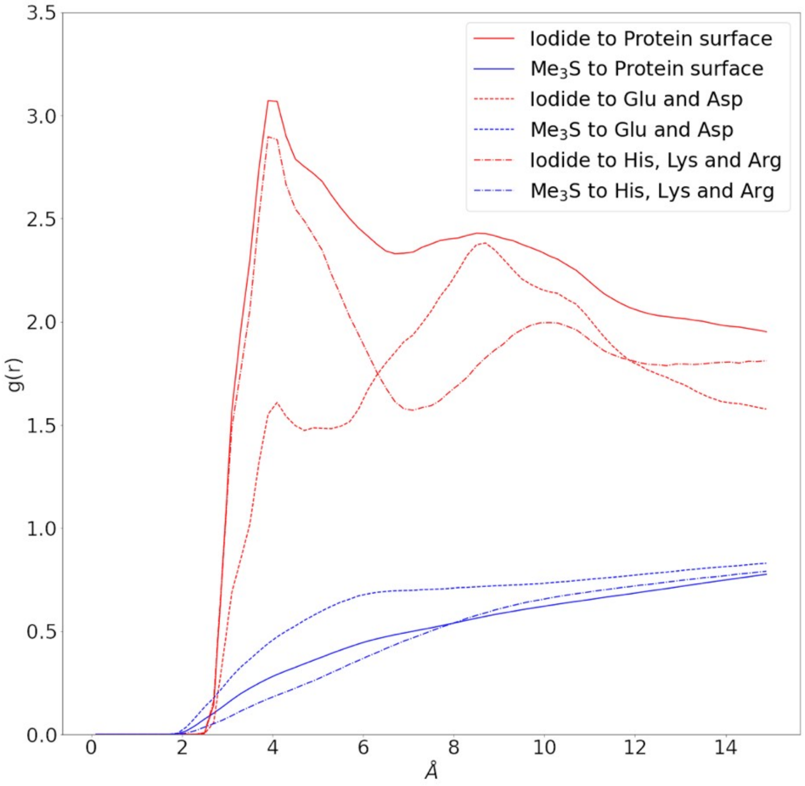

Iodide to Protein surface
Me_3S to Protein surface
Iodide to Glu and Asp
Me_3S to Glu and Asp
Iodide to His, Lys and Arg
Me_3S to His, Lys and Arg
g(r)
Å
0.0
0.5
1.0
1.5
2.0
2.5
3.0
3.5
0
2
4
6
8
10
12
14

Legend for following Figures 11, 12

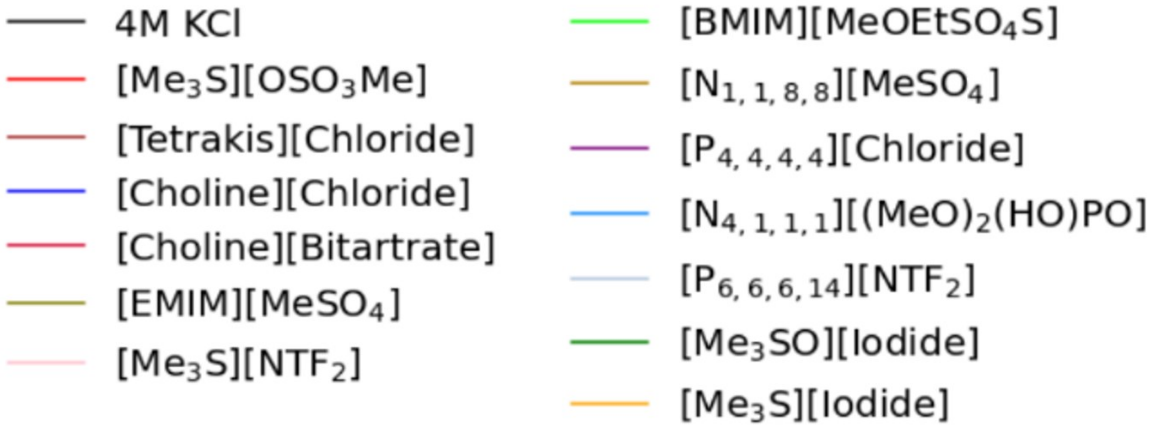

SI Figure S10. Legend for SI figures 11-13

RDFs and Survival Probabilities between IL cations and anions

SI Figure S11. RDFs of anions around cations in ionic liquids (**top**), SP of anions around cations (**bottom**). RDFs and SPs of ions around counter-ions combined can be indicative of ion-pair and ion-patch formation. The ability of forming long-lived ion pairs as found for $[BMIM]^+[MeOEtSO_4]^-$ (medium-range RDF, high SP) rather than ion patches as found for $[N_{1,1,1,4}]^+[(MeO)_2OPO]^-$ (high RDF, high SP) or short-lived ion pairs as found for $[Me_3S]^+[MeSO_4]^-$ (low RDF, low SP) allows the cation to correlate to the anions SP on the protein surface. In congruence with this, $[BMIM]^+$ is found with twice the probability (RDF between IL ions) in proximity to the protein surface compared to $[DiMIM]^+$. Comparing systems $[DiMIM]^+[MeSO_4]^-$ and $[BMIM]^+[MeOEtSO_4]^-$ shows that SP between ions is a direct indicator for the interaction of ion pairs or ion patches with the protein surface. The longer-lived interaction between ions $[BMIM]^+$ and $[MeOEtSO_4]^-$ and the exceptional coordination by $[MeOEtSO_4]^-$ to the protein surface affects $[BMIM]^+$ SP around *Hv*ADH2 (see figure below) to be the longest for all cations not paired with $[Cl]^-$ and the same as the maximum observed for cations paired with $[Cl]^-$. While $[DiMIM]^+$ has an almost doubled higher probability in interacting with its counter-anion than $[BMIM]^+$, the interaction duration is half as long and leads to a lessened influence of $[MeSO_4]^-$ on $[DiMIM]^+$ at the protein surface (see also figure below). This is not only observed for the systems $[DiMIM]^+[MeSO_4]^-$ and $[BMIM]^+[MeOEtSO_4]^-$, but becomes even more apparent with the systems where a prolonged interaction exists between ions. Both ions in IL $[P_{6,6,6,14}]^+[NTf_2]^-$ which interact longest with each other, have a similarly long interaction with the protein surface (see also figure below). In terms of similarity of counter-ion interaction with the protein surface this system is followed by $[N_{1,1,1,4}]^+[(MeO)_2OPO]^-$ and $[N_{1,1,8,8}]^+[MeSO_4]^-$ (see figure below). The discrepancy with regards to similarity of counter-ion interaction with the protein between $[BMIM]^+[MeOEtSO_4]^-$ and $[N_{1,1,1,4}]^+[(MeO)_2OPO]^-$ can be explained by looking at SPs and RDFs of these two systems. While the SP between the ions of $[N_{1,1,1,4}]^+[(MeO)_2OPO]^-$ is comparable to $[BMIM]^+[MeOEtSO_4]^-$, the probability of finding $[(MeO)_2OPO]^-$ around $[N_{1,1,1,4}]^+$ is twice as high as for $[MeOEtSO_4]^-$ around $[BMIM]^+$.

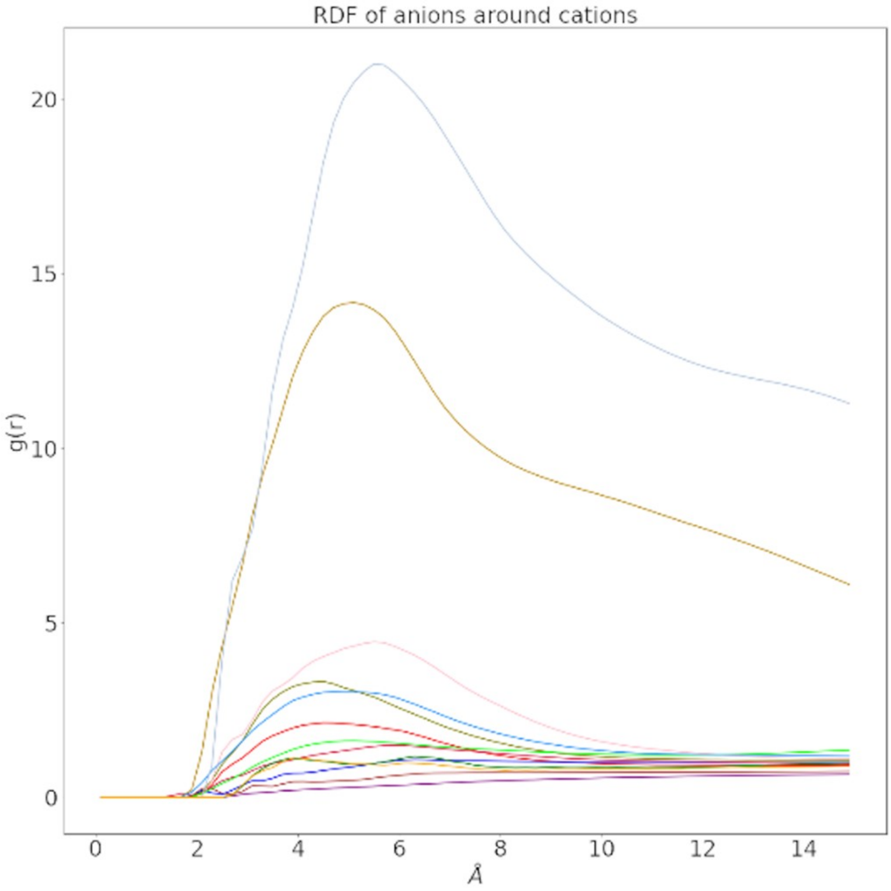

RDF of anions around cations
g(r)
0
5
10
15
20
0
2
4
6
8
10
12
14
Å

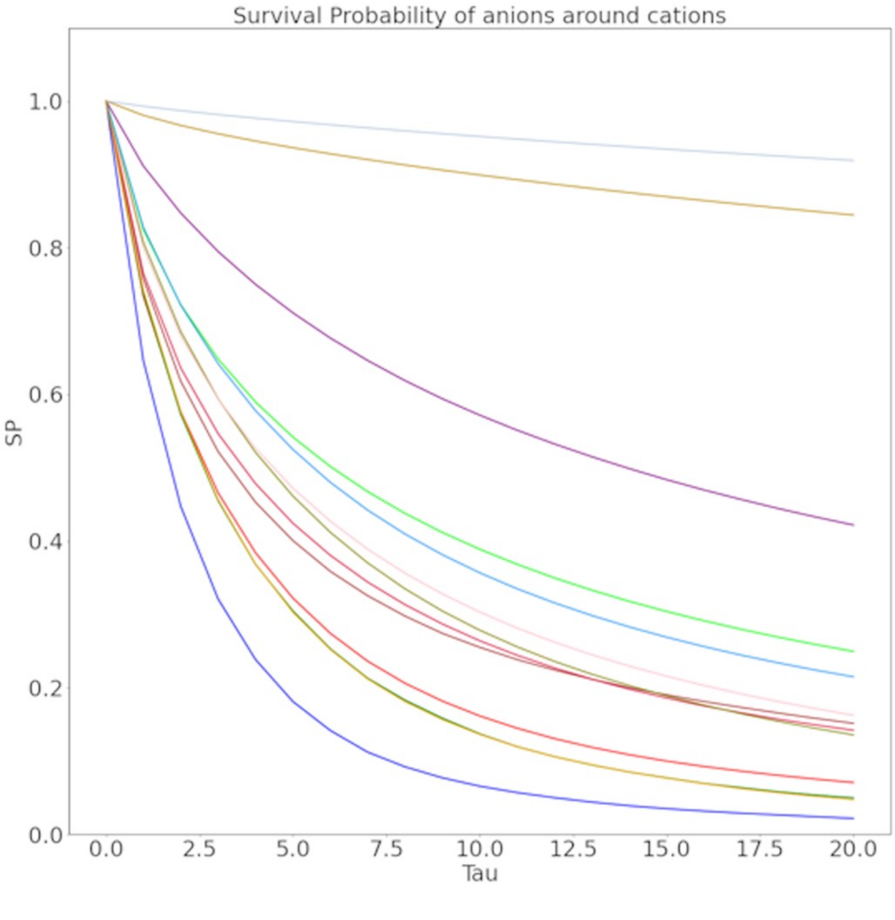

Survival Probability of anions around cations
SP
0.0
0.2
0.4
0.6
0.8
1.0
0.0
2.5
5.0
7.5
10.0
12.5
15.0
17.5
20.0
Tau

SPs of IL cations and anions around *Hv*ADH2

SI Figure S12. Survival probability of IL cations **(top)** and anions **(bottom)** around *Hv*ADH2. SP of [I]$^-$ around the protein is marginally increased when paired with $[Me_3S]^+$ compared to when paired with $[Me_3SO]^+$, while SP of $[Me_3S]^+$ is marginally decreased compared to $[Me_3SO]^+$, which indicates a less strong reciprocal effect within the system $[Me_3SO]^+[I]^-$, possibly owed to the presence of the polarity-inducing oxygen. Least probable interaction of $[Me_3S]^+$ with the protein is observed when paired with $[MeSO_4]^-$. By comparison a slightly higher probability of $[Me_3S]^+$ around *Hv*ADH2 is found when paired with [I]$^-$ and $[NTf_2]^-$, increasing in this order.

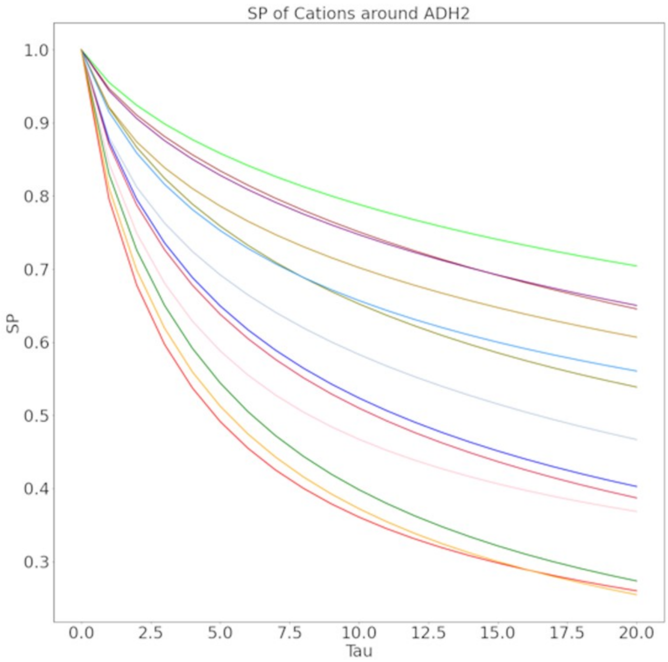

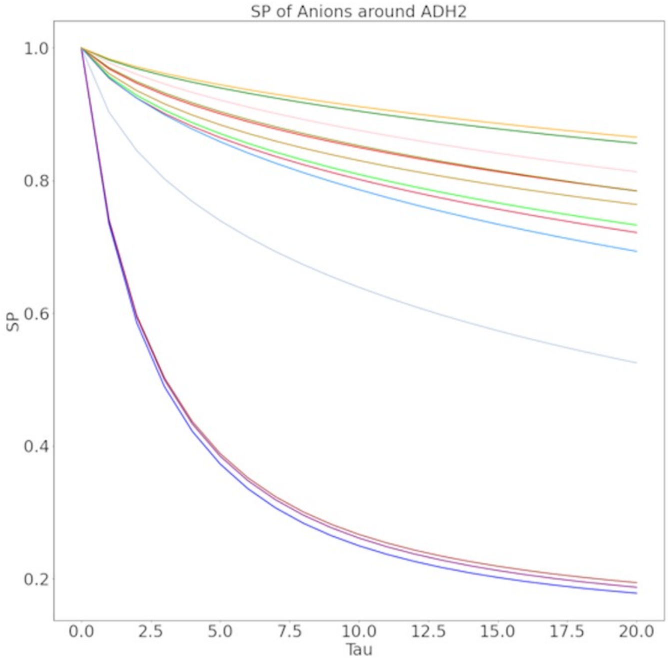

Radial distribution functions of K^+ and O_w around Cγ/Cδ of Glu/Asp residues, Gln/Asn residues and between K^+- K^+, K^+- O_w and O_w- O_w

SI Figure S13. RDFs obtained from MD simulation of the native *Hv*ADH2 system for K^+ and H_2O. Three association distances are apparent from RDF values for K^+ around carboxylic acid residues, peaking at 2.7 Å, 3.7- 4.3 Å and 4.3- 6.0 Å respectively. Oxygens of water molecules (O_W) show discernible peaks at 1.5- 2.1 Å, 2.9- 3.1 Å, 3.3- 4.1 Å and bulk past 4.1 Å around ASP and GLU residues. O_W is surrounding K^+ in two layers, whereby the first is distinguished by two peaks at 2.9 Å and 3.3 Å, respectively, and the second hydration shell spans from 4.5- 6.5 Å. Inter-H_2O distances show peaks at 2.5 Å and between 5.1- 7.1 Å. Radial pairs between K^+ ions peak at 3.5- 5.3 Å, 5.7-6.3 Å, 6.5- 7.3 Å and 7.9- 8.5 Å.

K^+ associating in its second and/or third shell at 3.7-4.3 Å and 4.3-5.8 Å, respectively, may allow for the occupation of H_2O in the fourth hydration shell starting at 4.1 Å consistent with the second association distance between K^+ and H_2O of 3.3 Å. The association between K^+ and H_2O based on their third interaction distance between 4.5-6.5 Å may give rise to the separation of H_2O molecules according to their second interaction distance of 5.1-7.1 Å and separates K^+ ions according to their third interaction distance of 5.1-7.1 Å. Direct association of water to COO^- at 1.5-2.1 Å may account for the second coordination shell of K^+ around COO^- at 3.7-4.3 Å, in concordance with the first hydration shell of H_2O around K^+ of 2.9 Å. The second hydration shell of H_2O around COO^- at 2.9-3.1 Å may account for the third hydration shell of H_2O to COO^- at 3.3-4.1 Å in concordance with the inter H_2O distance of 2.5 Å. Farther than 6.0 Å from COO^-, K^+ and H_2O can assume either of their three association distances, which is also in congruence with the 'spikey' profile of K^+ ions to K^+ ions association distance between 5.7-6.3 Å and 6.5-7.3 Å. These distinct peaks merge in bulk water (< 500 Å around COO^-). Closer to the protein surface this allows for varying hydration shells of H_2O around COO^-. Further elaboration of how shell distances may explain or fit in with other shell distances in conjunction with SDFs is given in Figure 4 of the main manuscript.

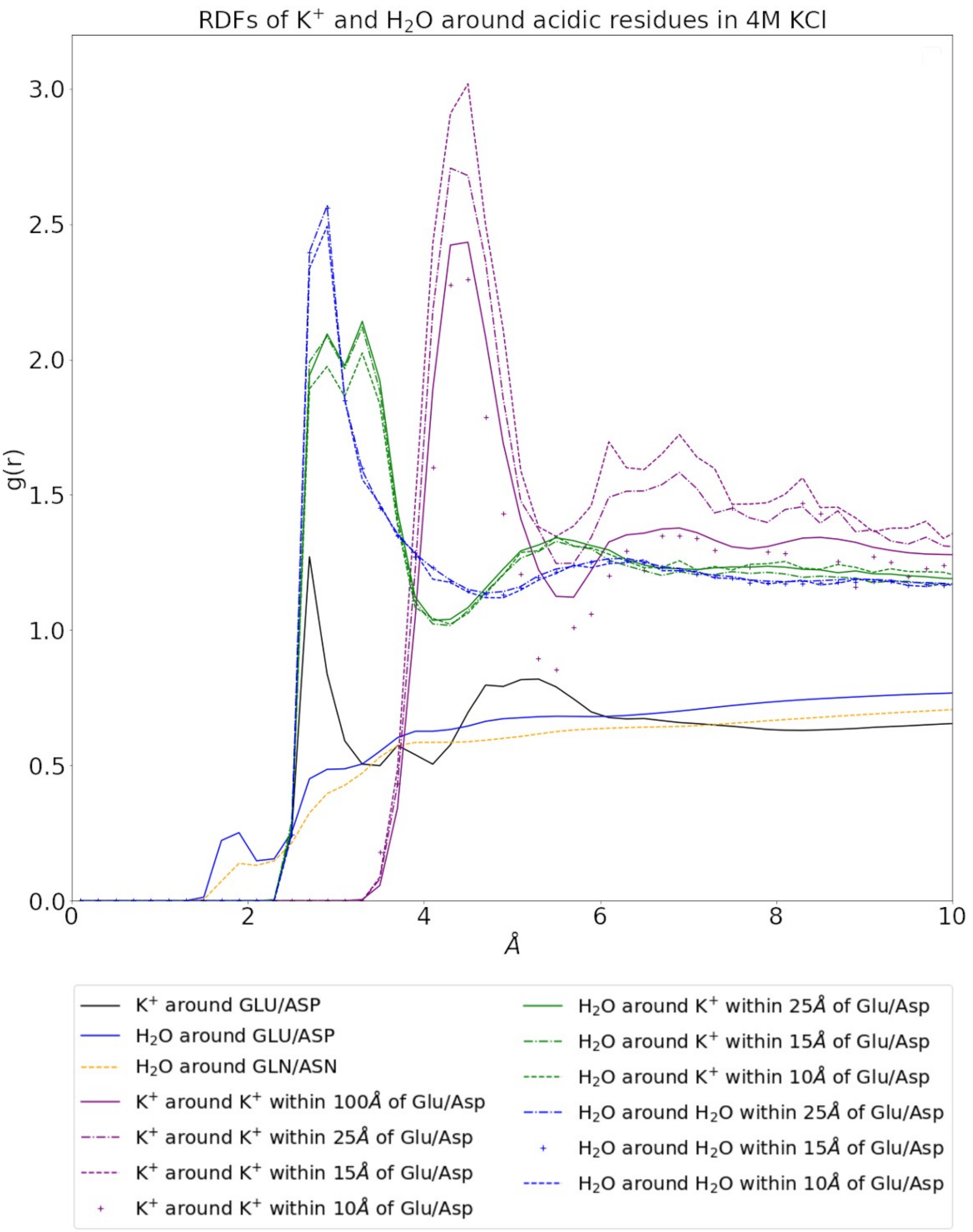
RDFs of K+ and H2O around acidic residues in 4M KCl
g(r)
Å
0.0
0.5
1.0
1.5
2.0
2.5
3.0
0
2
4
6
8
10
K+ around GLU/ASP
H2O around GLU/ASP
H2O around GLN/ASN
K+ around K+ within 100Å of Glu/Asp
K+ around K+ within 25Å of Glu/Asp
K+ around K+ within 15Å of Glu/Asp
K+ around K+ within 10Å of Glu/Asp
H2O around K+ within 25Å of Glu/Asp
H2O around K+ within 15Å of Glu/Asp
H2O around K+ within 10Å of Glu/Asp
H2O around H2O within 25Å of Glu/Asp
H2O around H2O within 15Å of Glu/Asp
H2O around H2O within 10Å of Glu/Asp

RDFs of K^+, Cl^- and O_w around Cγ/Cδ of Glu/Asp and Cγ/Cε/Cζ of His/Lys/Arg residues

SI Figure S14. RDFs of K^+ and Cl^- ions **(top)** and H_2O **(bottom)** around surface residues of *Hv*ADH2. K^+ ions preferentially coordinate to negatively charged residues and are removed from positively charged residues. Cl^- ions preferentially coordinate to positively charged residues, but are less present than K^+ around uncharged surfaces. H_2O has a first distinct solvation shell at ~ 1.9 Å followed by less distinct solvation shells. Overall, the profile is similar to mesophilic surfaces, [4] but water content is increased for halophilic surfaces.

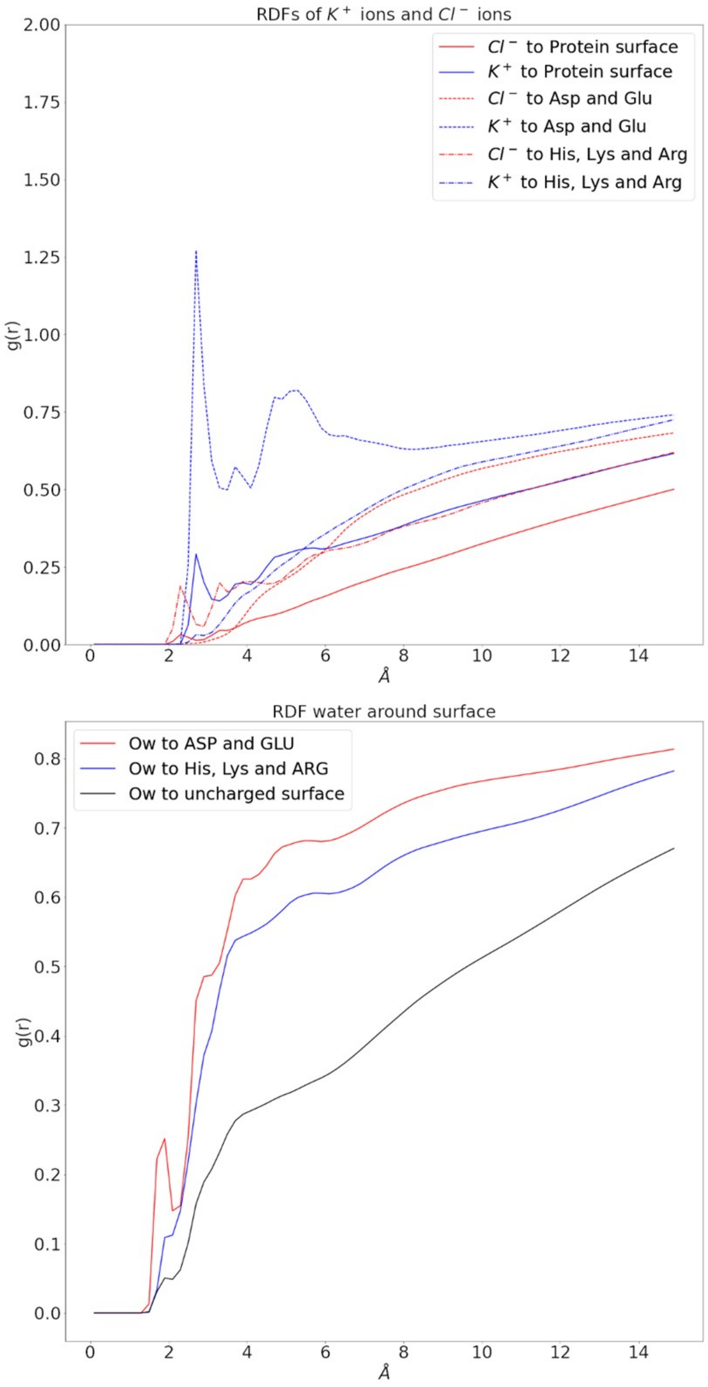

Spatial distribution functions around non-negatively charged residues

SI Figure S15. SDFs of non-acidic residues **(A)** Thr1073, **(B)** Lys1064, **(C)** Gln1070 and **(D)** Leu280. The polar uncharged side chain of Thr1073 and Gln1070 show residue specific coordination of K^+ ions. Coordination of K^+ ions is farthest removed around the positively charged residue Lys1064 and the apolar residue Leu280. However, solvent structure surrounding residues remains unbroken for all four residues. A highly interesting coordination is observed for Threonine, where the tetrahedral structure of the C_4 sidechain atom appears to subject the water structure in the second solvation shell as well as K^+ ion distribution to a coordination maintaining the character of a triad, in case of K^+ a double triad.

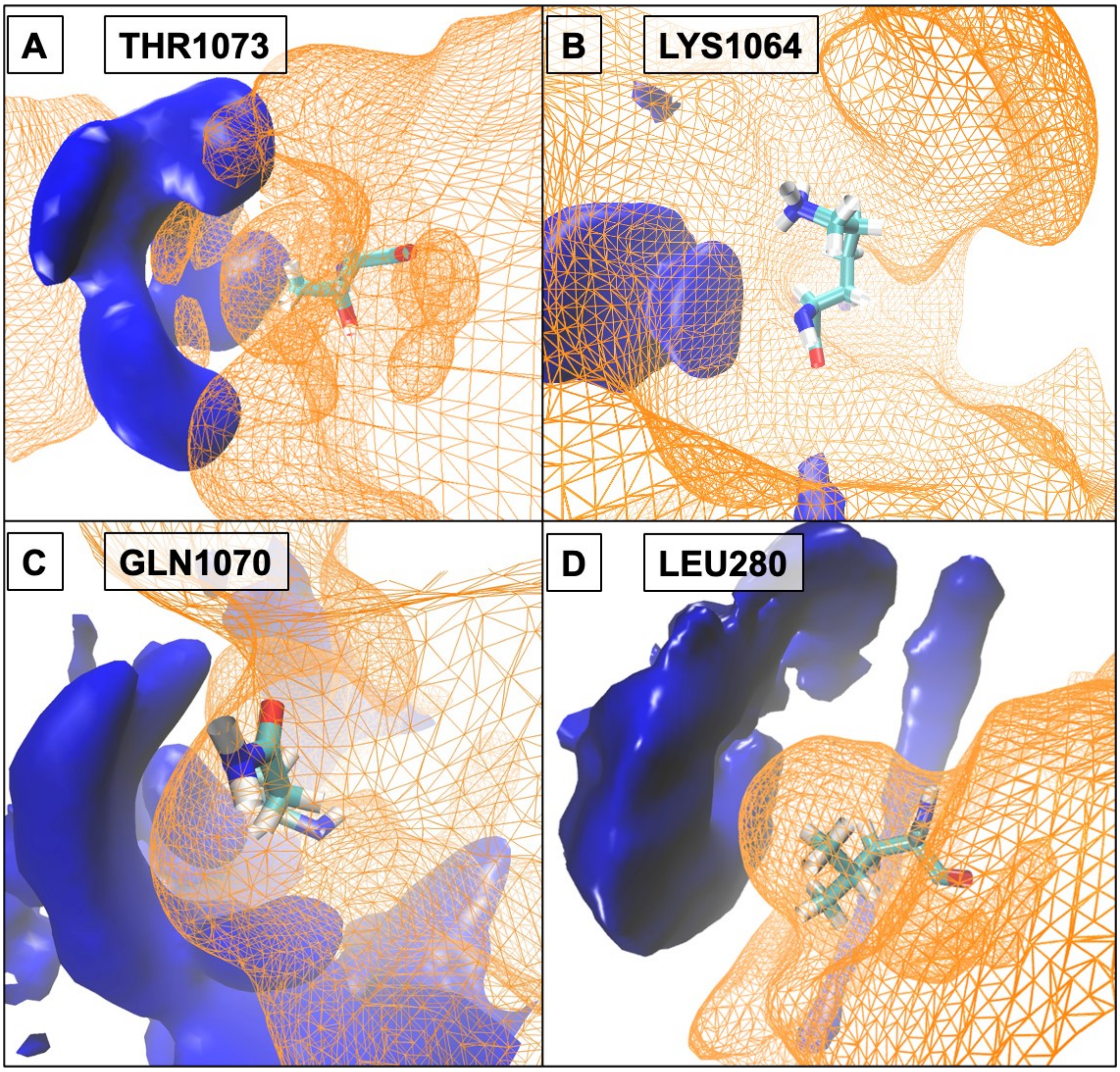

K^+ ion and water association to Glu1292 in monomer D

SI Figure S16. The change of bond distance between a K^+ ion and residue Glu245 **(top left)** and a K^+ ion and residue Glu1292 **(top right)** is plotted over the course of the trajectory. The change in bond distance of four different water molecules coordinating to the K^+ ion around Glu1292 **(middle and bottom rows)** is plotted over the course of the trajectory. The K^+ ions stay coordinated for the whole length of the simulation, and water molecules stay interstitial and coordinated for a prolonged time.

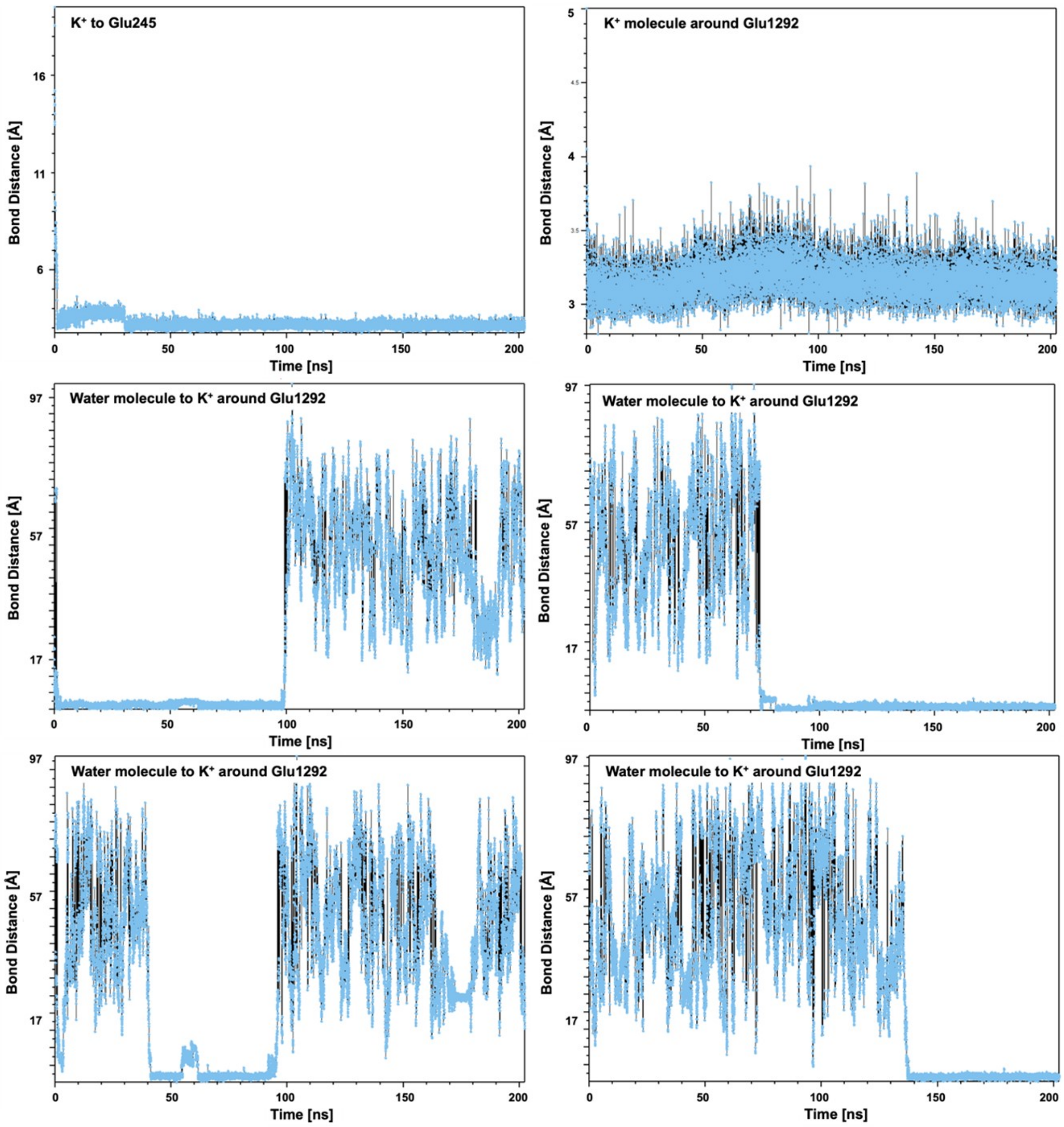

SPs of O_W and K^+ around Cγ/Cδ of Glu/Asp and Cγ/Cε/Cζ of His/Lys/Arg residues in the native system

SI Figure S17. Survival Probability of K^+ and H_2O around acidic and basic residues in the native system containing 4 M KCl.

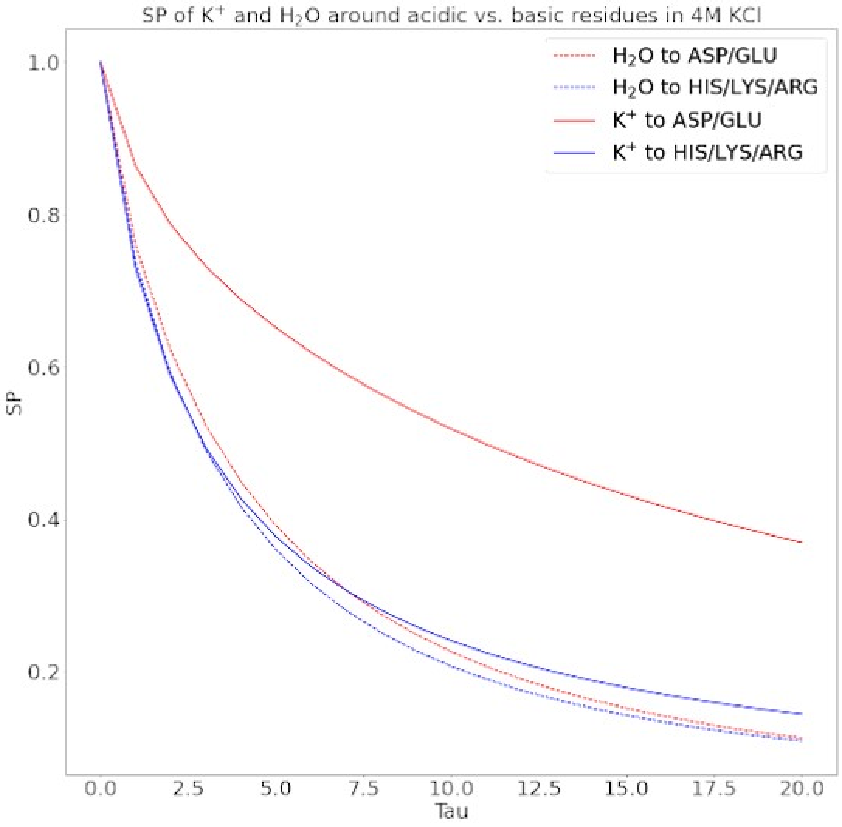

SPs of K^+ around Cγ/Cδ of Glu/Asp and Cγ/Cε/Cζ of His/Lys/Arg residues in ionic liquids

SI Figure S18. SP of K^+ around acidic **(top)** and basic **(bottom)** residues.

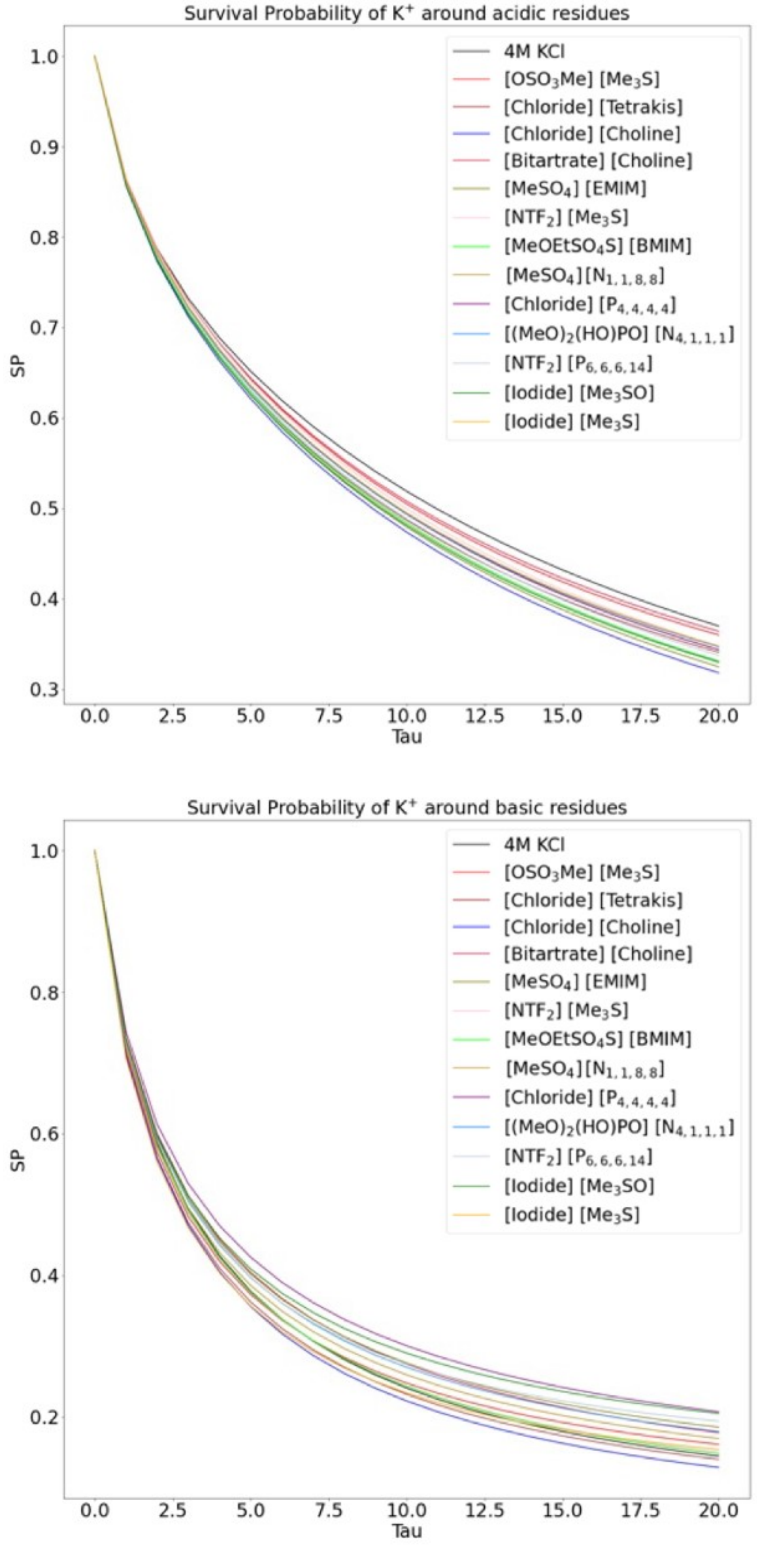

RDFs of K^+ to Cγ/Cε/Cζ of His/Lys/Arg residues in ionic liquids

SI Figure S19. RDF of K^+ around basic residues.

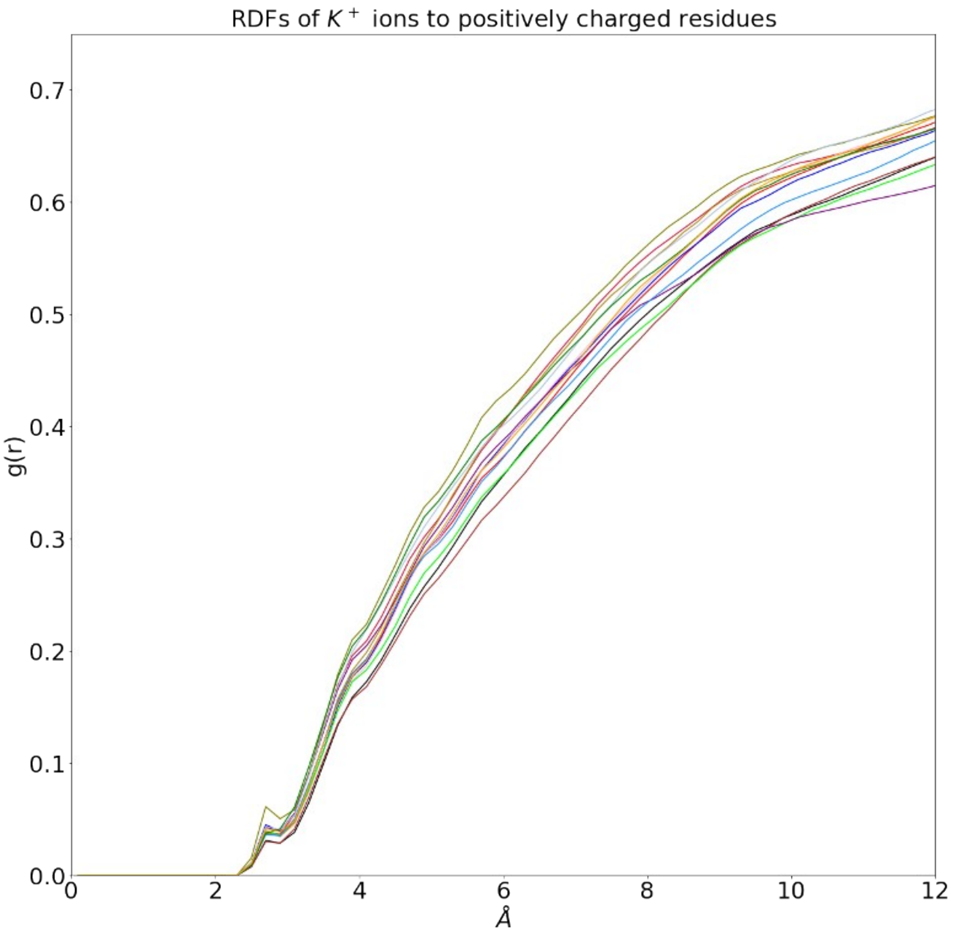

RDFs of Cl^- to Cγ/Cδ of Glu/Asp and Cγ/Cε/Cζ of His/Lys/Arg residues in ionic liquids

SI Figure S20. RDF of Cl^- around acidic **(top)** and basic **(bottom)** residues.

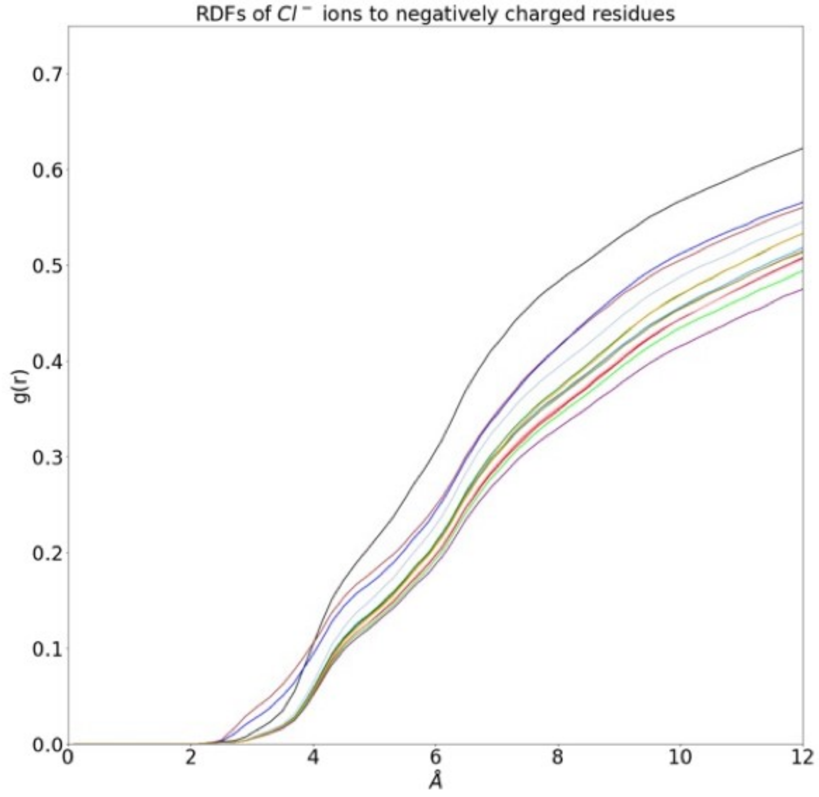

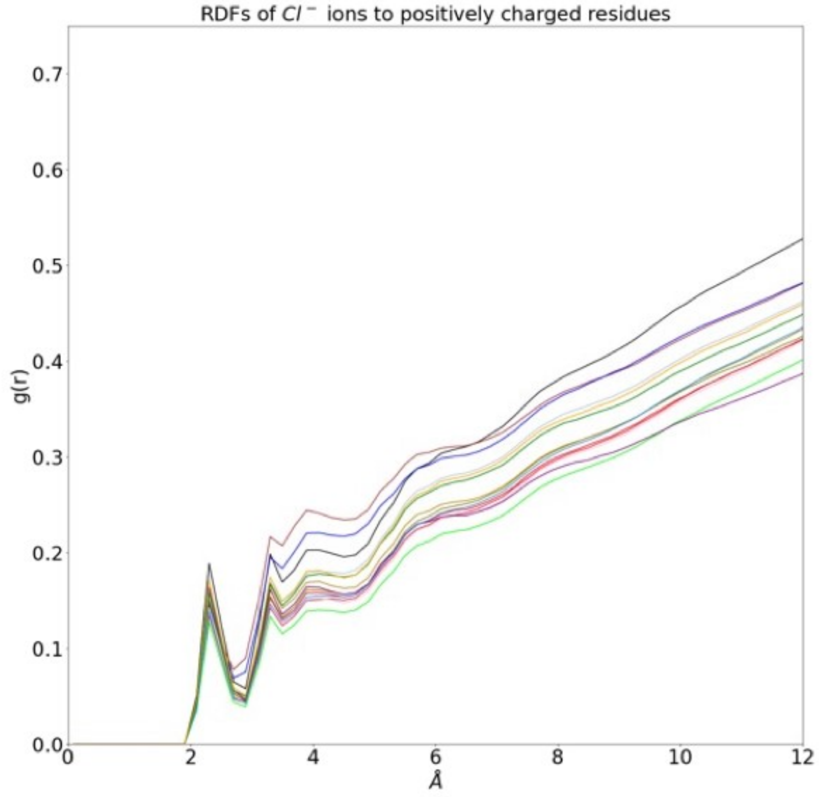

SPs of O_w around Cγ/Cδ of Glu/Asp and Cγ/Cε/Cζ of His/Lys/Arg residues in ionic liquids

SI Figure S21. SP of O_W around acidic **(top)** and basic **(bottom)** residues. SP of O_W around IL cations and counter-anions does not correlate, despite the long-lived proximity of some cations around anions (e.g. the hydrophobic ILs $[P_{6,6,6,14}]^+[NTf_2]^-$ and $[N_{1,1,8,8}]^+[MeSO_4]^-$). This indicates an interaction with water that is ion type specific, and comparison of SP of H_2O around cations $[Me_3S]^+$ and $[Me_3SO]^+$ groups them together regardless of their pairing with a charge localised counter-anion $[I]^-$, a charge distributed ion $[MeSO_4]^-$ or a water-immiscible anion $[NTf_2]^-$. This is also true for $[Choline]^+$ in both systems, once paired with a charge localised anion $[Cl]^-$ and once with another hydroxyl-functionalised anion $[Bitartrate]^-$.

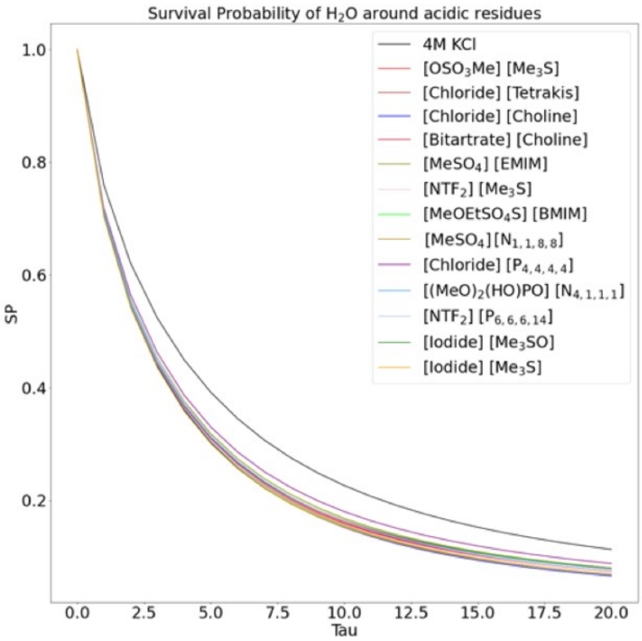

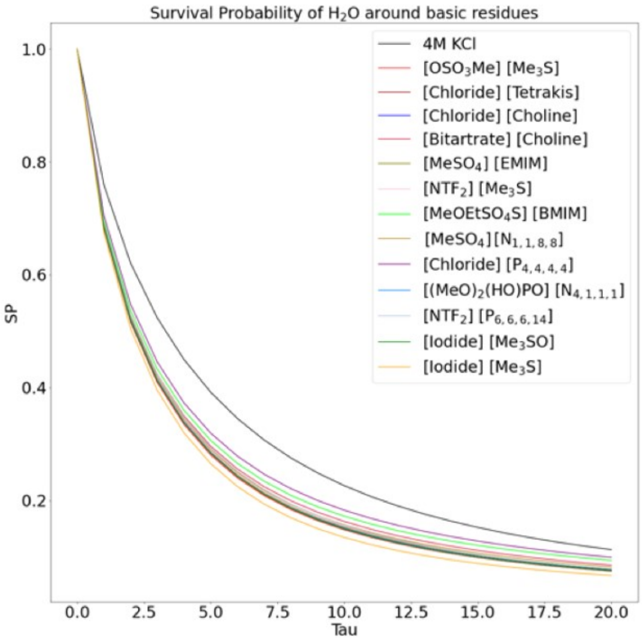

Ramachandran plot

SI Figure S22. Ramachandran plot of the HvADH2 tetramer derived by PROCHECK to validate the homology model derived from I-Tasser.

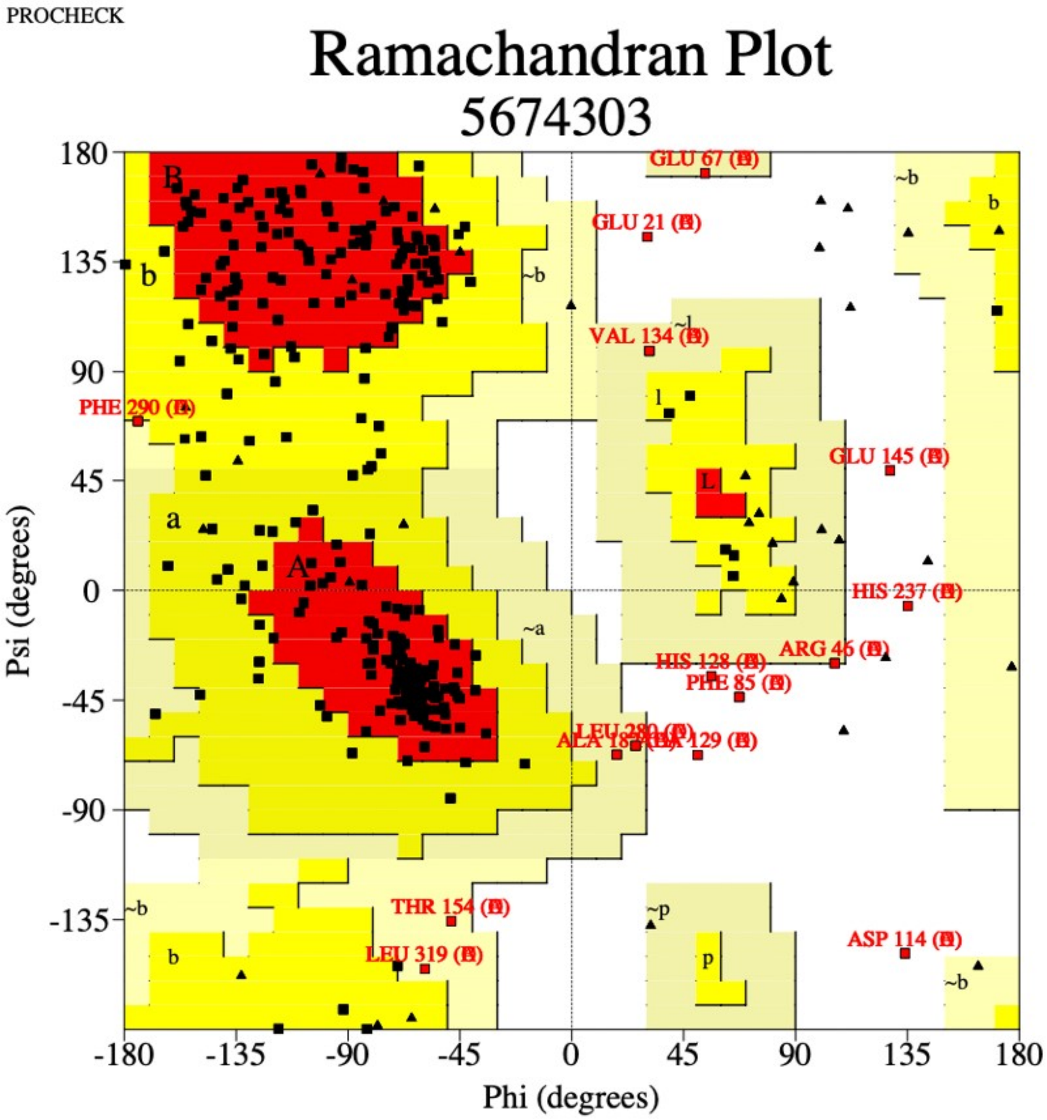

Plot statistics

Residues in most favoured regions [A,B,L]	864	74.7%
Residues in additional allowed regions [a,b,l,p]	232	20.1%
Residues in generously allowed regions [~a,~b,~l,~p]	32	2.8%
Residues in disallowed regions	28	2.4%
	----	------
Number of non-glycine and non-proline residues	1156	100.0%
Number of end-residues (excl. Gly and Pro)	19	
Number of glycine residues (shown as triangles)	164	
Number of proline residues	68	

Total number of residues	1407	

Based on an analysis of 118 structures of resolution of at least 2.0 Angstroms and R-factor no greater than 20%, a good quality model would be expected to have over 90% in the most favoured regions.

References

1. Przypis, M., et al., *Inexpensive and tuneable protic ionic liquids based on sulfuric acid for the biphasic synthesis of alkyl levulinates.* Journal of Molecular Liquids, 2020. **308**: p. 113166.
2. Gontrani, L., *Choline-amino acid ionic liquids: past and recent achievements about the structure and properties of these really ``green''chemicals.* Biophysical Reviews, 2018. **10**(3): p. 873--880.
3. Zhang, S., et al., *Hydroxyl Ionic Liquids: The Differentiating Effect of Hydroxyl on Polarity due to Ionic Hydrogen Bonds between Hydroxyl and Anions.* The Journal of Physical Chemistry B, 2010. **114**(11): p. 3912--3920.
4. Dahanayake, J.N. and K.R. Mitchell-Koch, *Entropy connects water structure and dynamics in protein hydration layer.* Physical Chemistry Chemical Physics, 2018. **20**(21): p. 14765--14777.
