# Supplementary figures and images for: Ion-combination specific effects driving the enzymatic activity of halophilic alcohol dehydrogenase 2 from Haloferax volcanii in aqueous ionic liquid solvent mixtures
Source: RSC Sustain. 2024 Jul 8;2(9):2559–80. doi: 10.1039/d3su00412k (PMC11353702; doi:10.1039/d3su00412k)

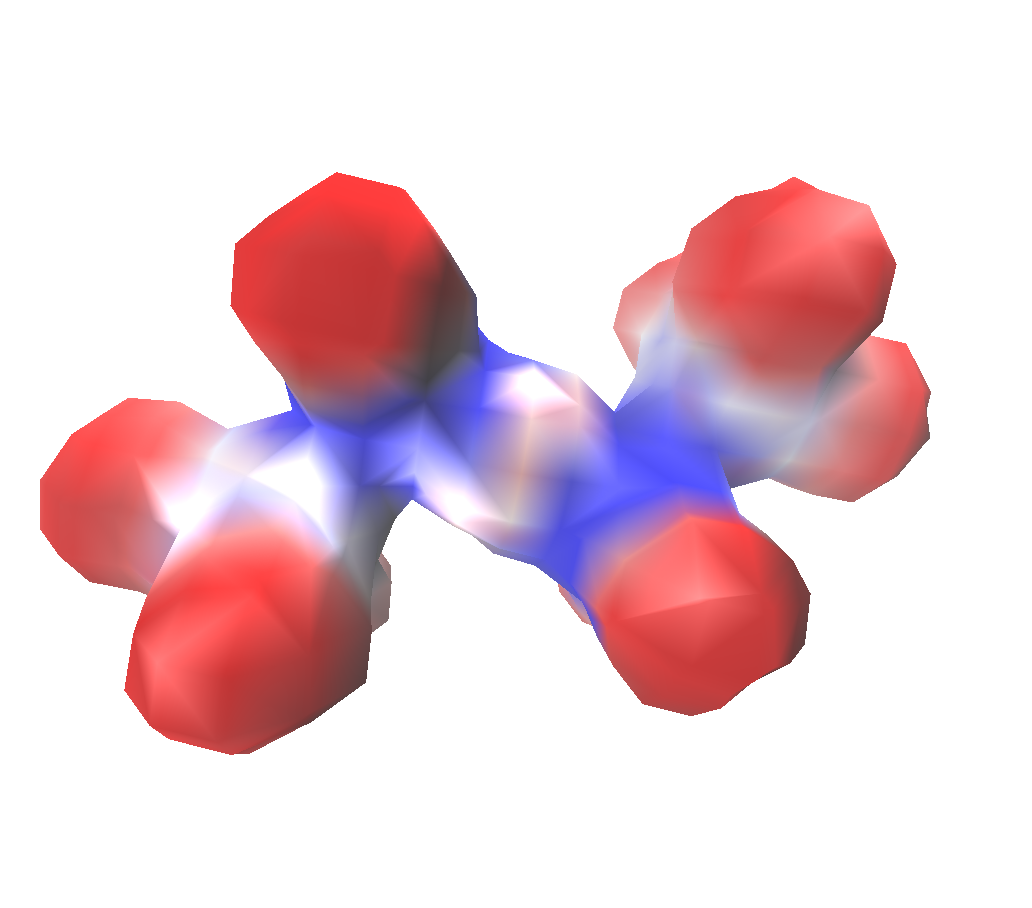

Supplement: SU-002-D3SU00412K-s004 [file SU-002-D3SU00412K-s004.zip › IonDescriptors/anions_MEP_processed/ntf.png]

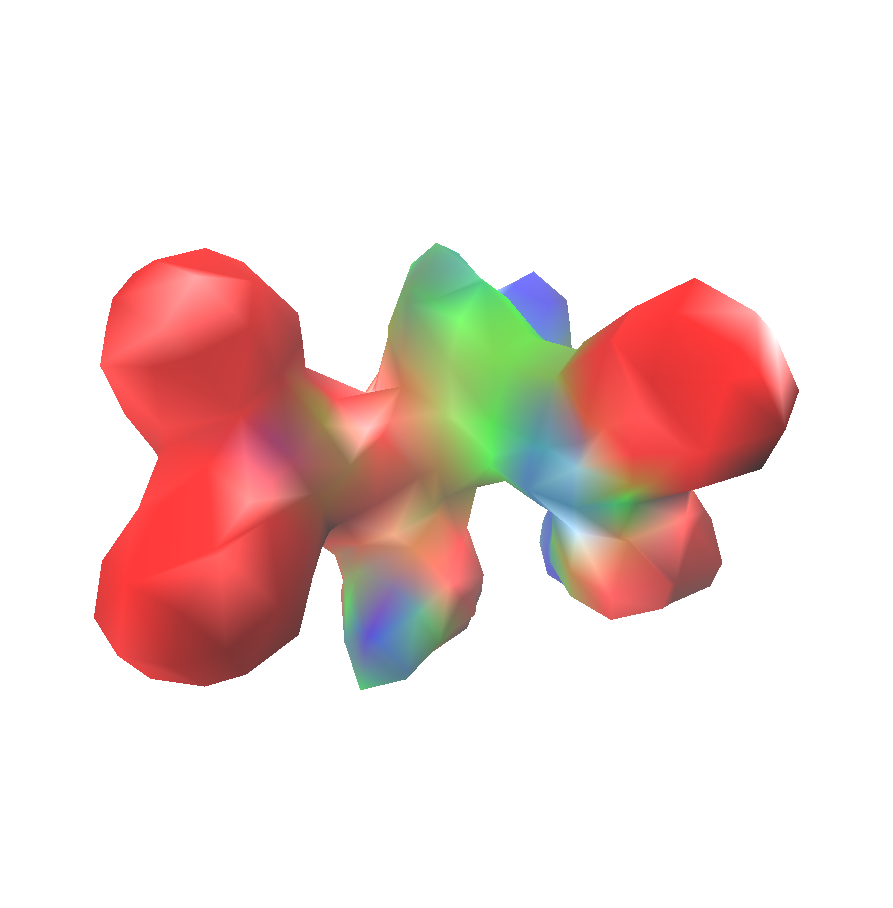

Supplement: SU-002-D3SU00412K-s004 [file SU-002-D3SU00412K-s004.zip › IonDescriptors/anions_MEP_processed/bit_RGB.png]

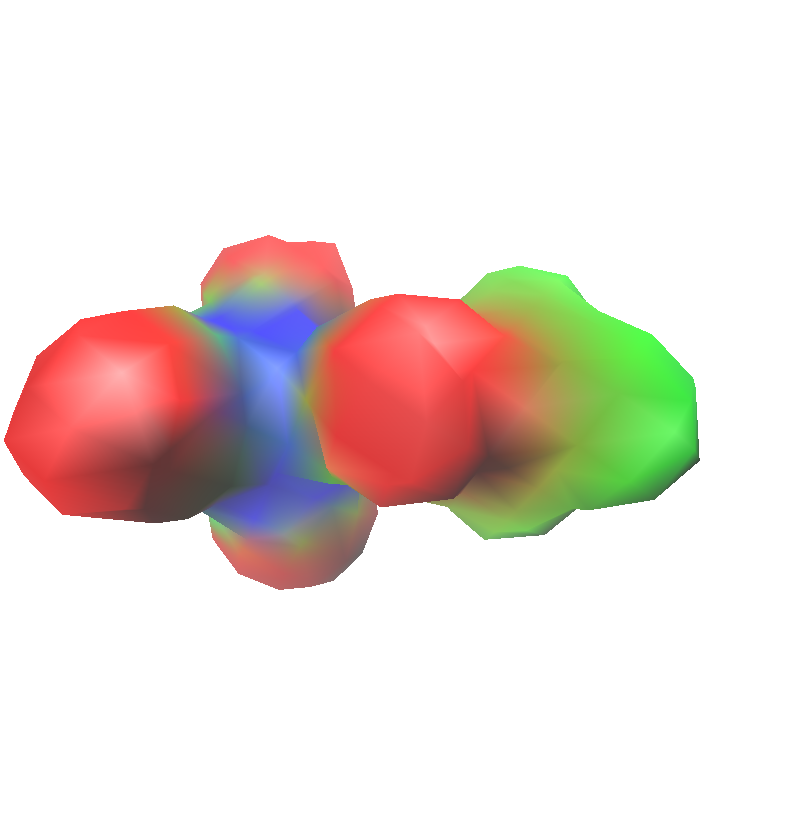

Supplement: SU-002-D3SU00412K-s004 [file SU-002-D3SU00412K-s004.zip › IonDescriptors/anions_MEP_processed/osm_RGB.png]

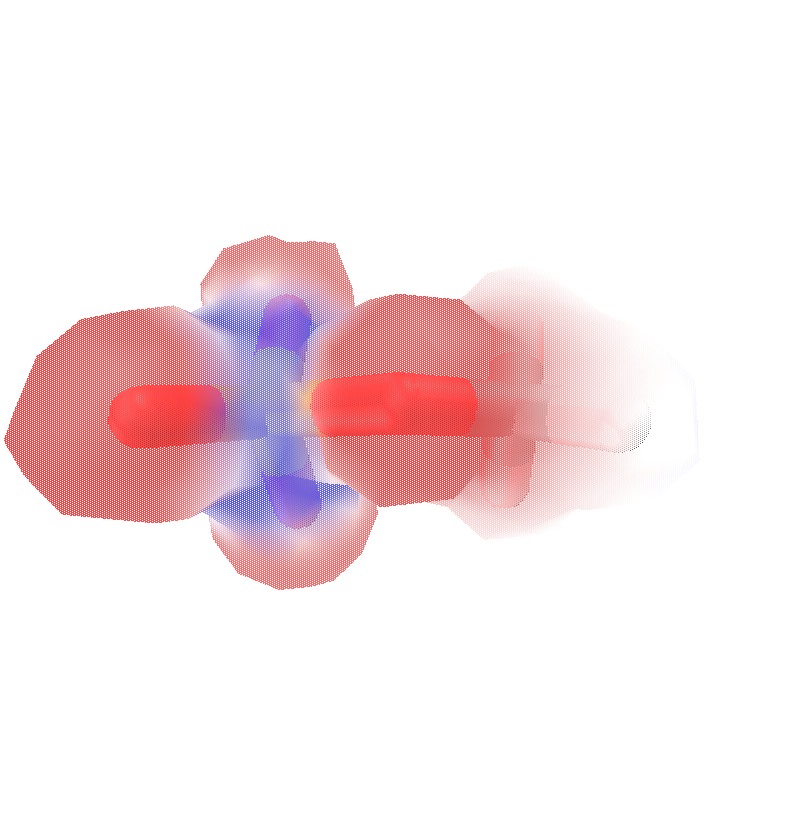

Supplement: SU-002-D3SU00412K-s004 [file SU-002-D3SU00412K-s004.zip › IonDescriptors/anions_MEP_processed/osm_ol.png]

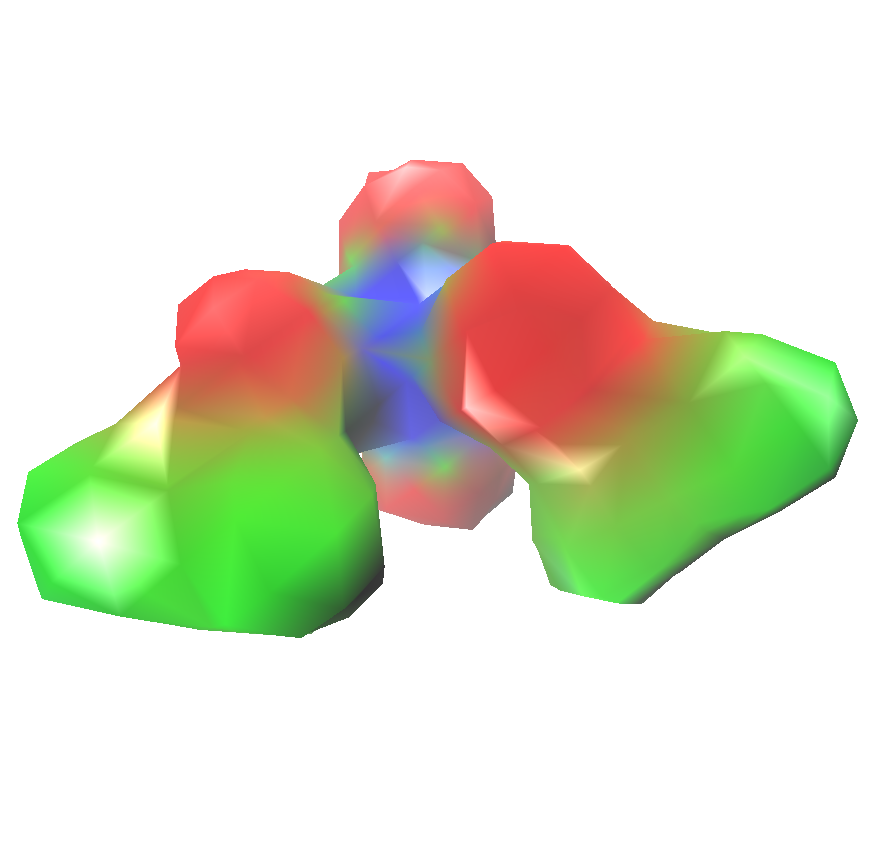

Supplement: SU-002-D3SU00412K-s004 [file SU-002-D3SU00412K-s004.zip › IonDescriptors/anions_MEP_processed/mop_RGB.png]

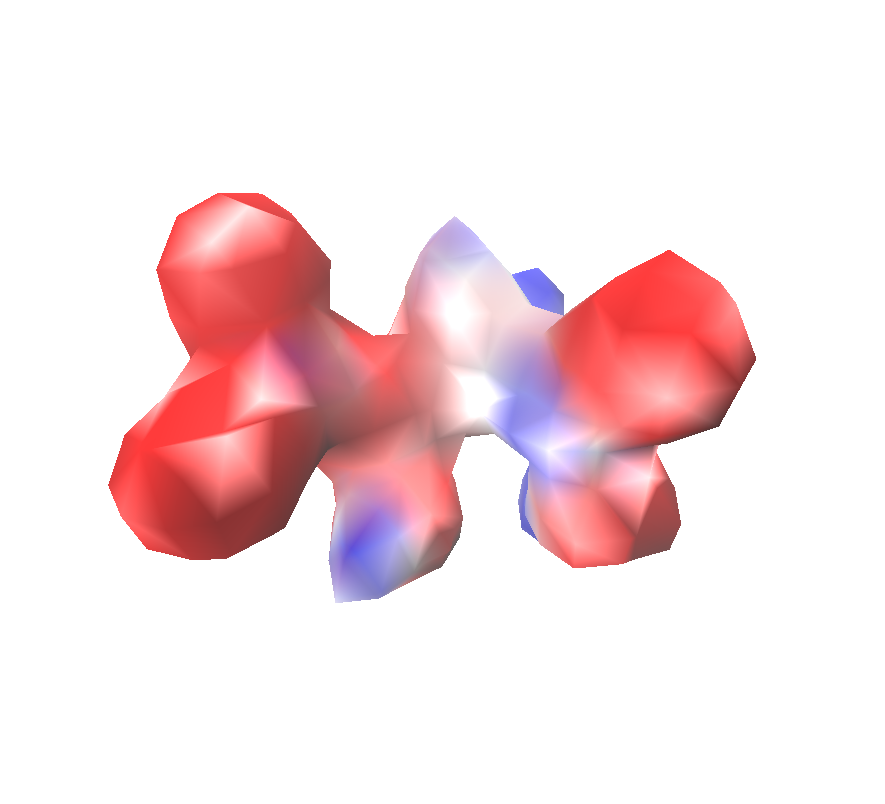

Supplement: SU-002-D3SU00412K-s004 [file SU-002-D3SU00412K-s004.zip › IonDescriptors/anions_MEP_processed/bit.png]

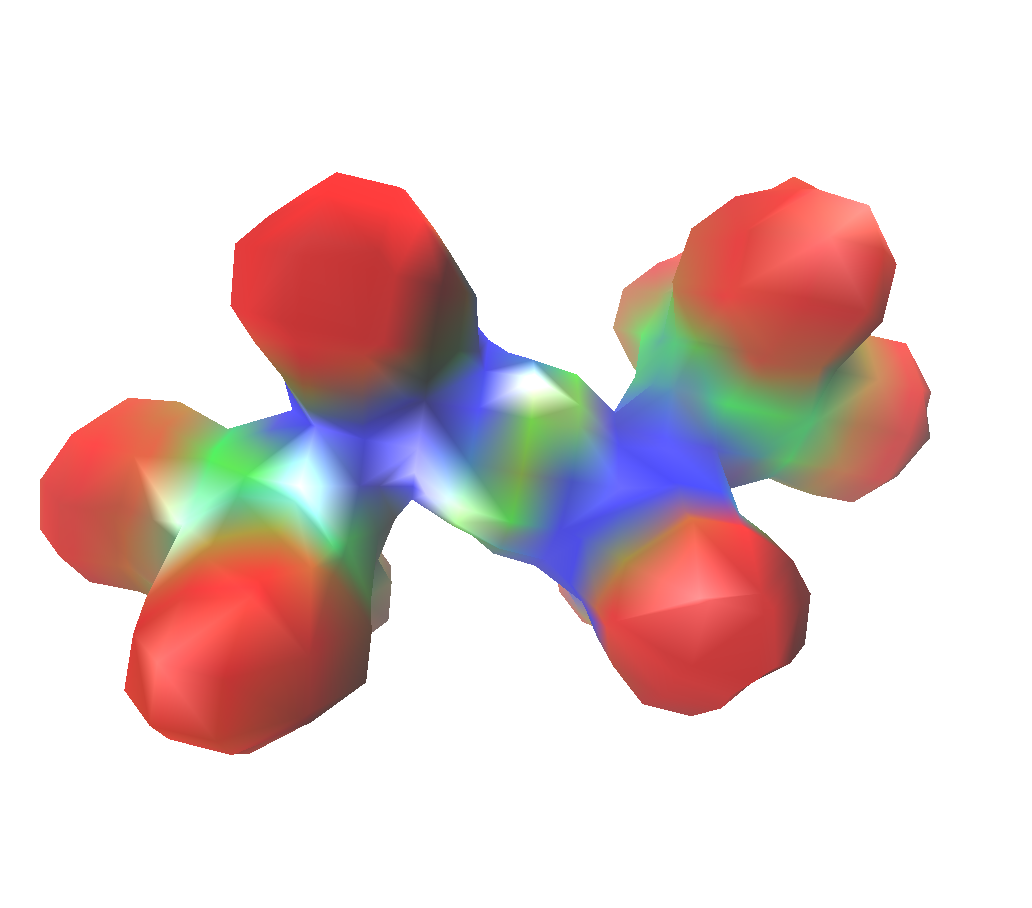

Supplement: SU-002-D3SU00412K-s004 [file SU-002-D3SU00412K-s004.zip › IonDescriptors/anions_MEP_processed/ntf_RGB.png]

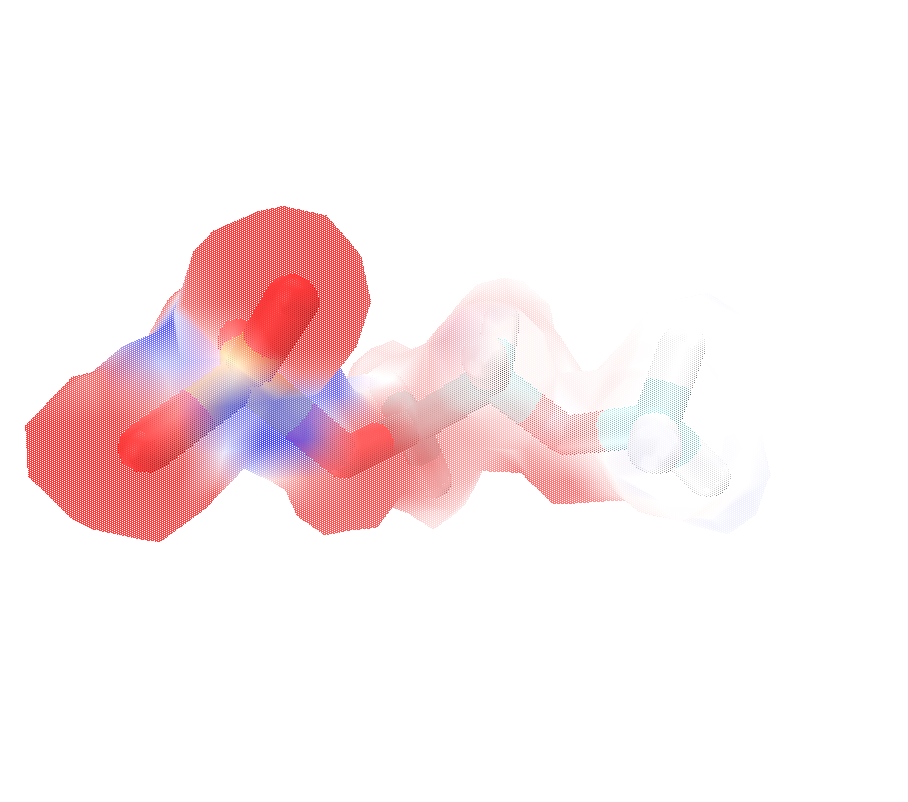

Supplement: SU-002-D3SU00412K-s004 [file SU-002-D3SU00412K-s004.zip › IonDescriptors/anions_MEP_processed/met_ol.png]

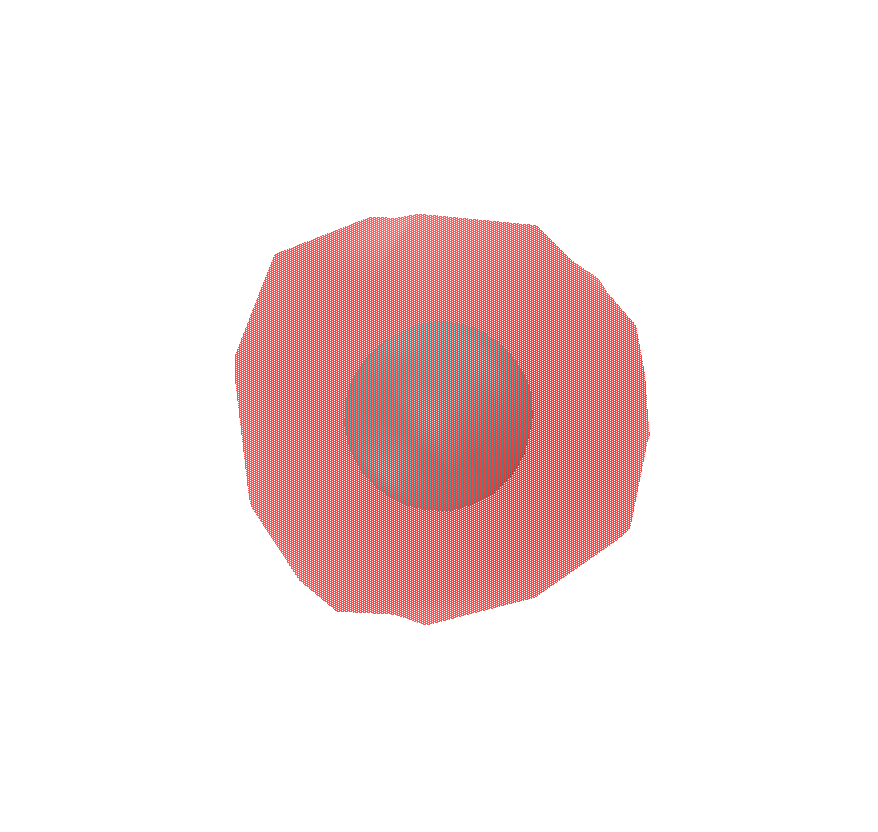

Supplement: SU-002-D3SU00412K-s004 [file SU-002-D3SU00412K-s004.zip › IonDescriptors/anions_MEP_processed/chl_ol.png]

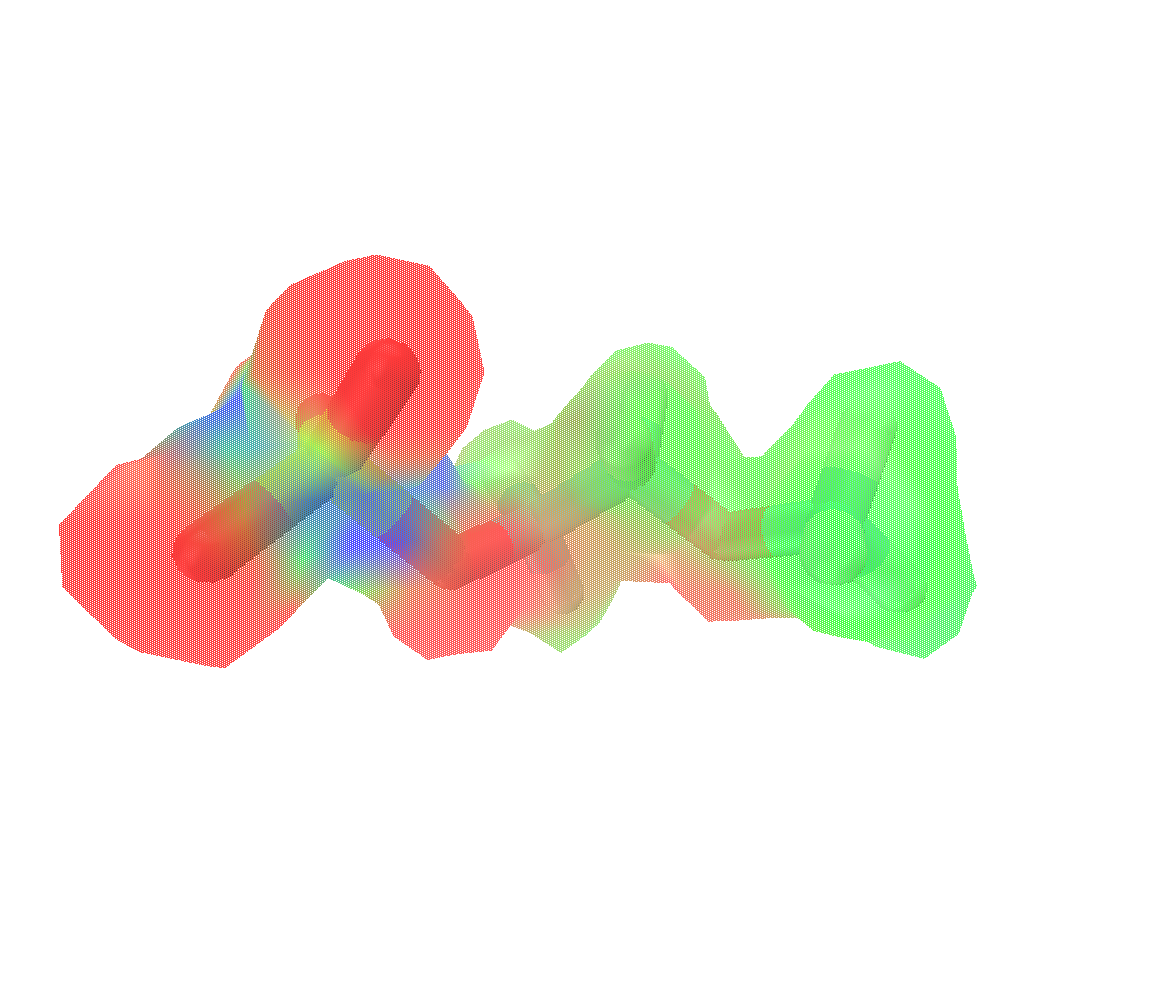

Supplement: SU-002-D3SU00412K-s004 [file SU-002-D3SU00412K-s004.zip › IonDescriptors/anions_MEP_processed/met_ol_RGB.png]

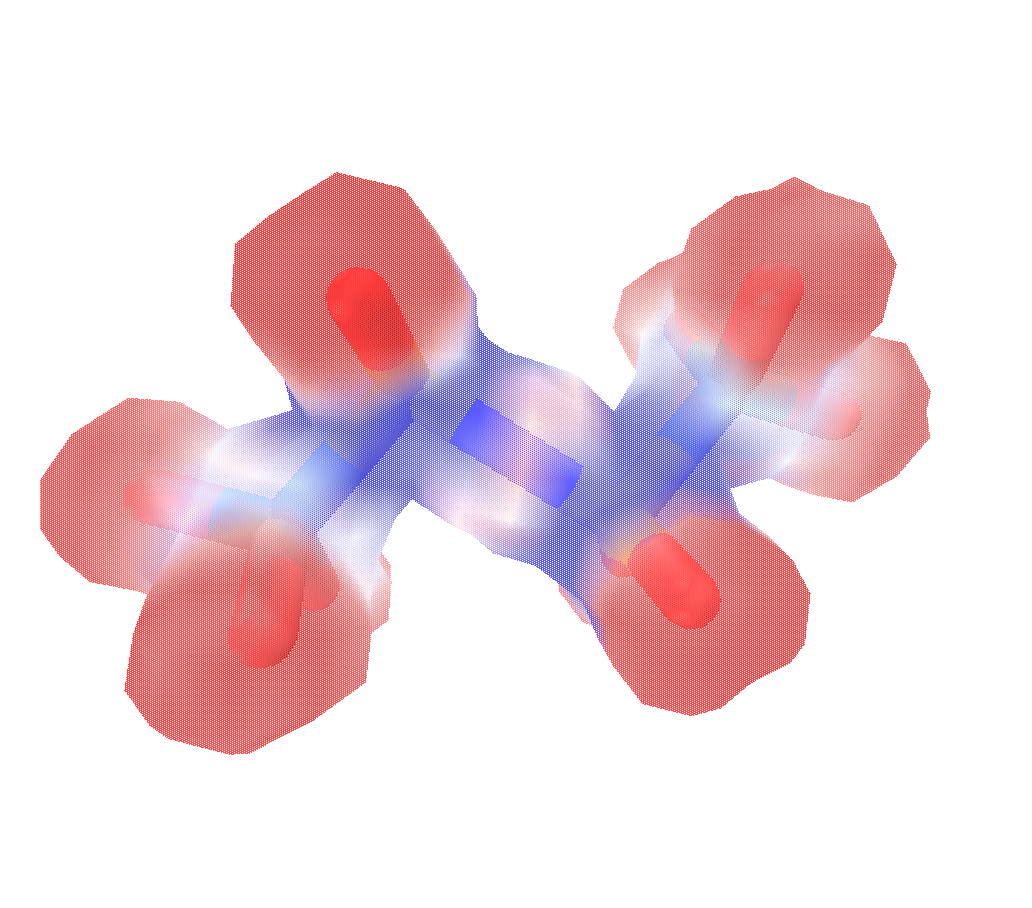

Supplement: SU-002-D3SU00412K-s004 [file SU-002-D3SU00412K-s004.zip › IonDescriptors/anions_MEP_processed/ntf_ol.png]

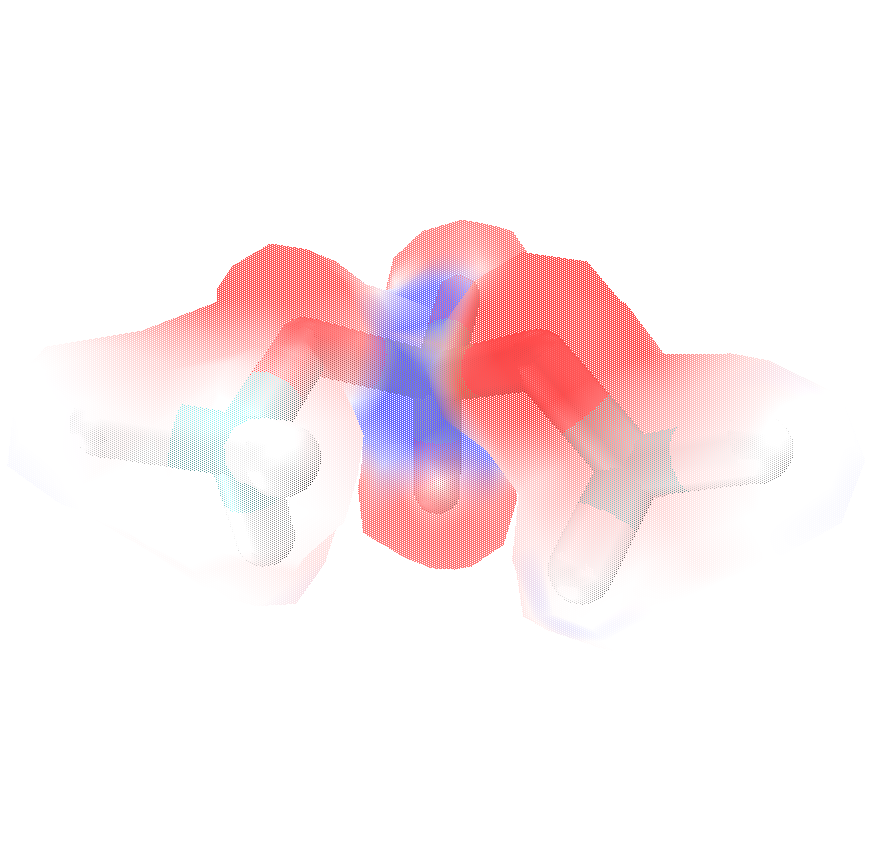

Supplement: SU-002-D3SU00412K-s004 [file SU-002-D3SU00412K-s004.zip › IonDescriptors/anions_MEP_processed/mop_ol.png]

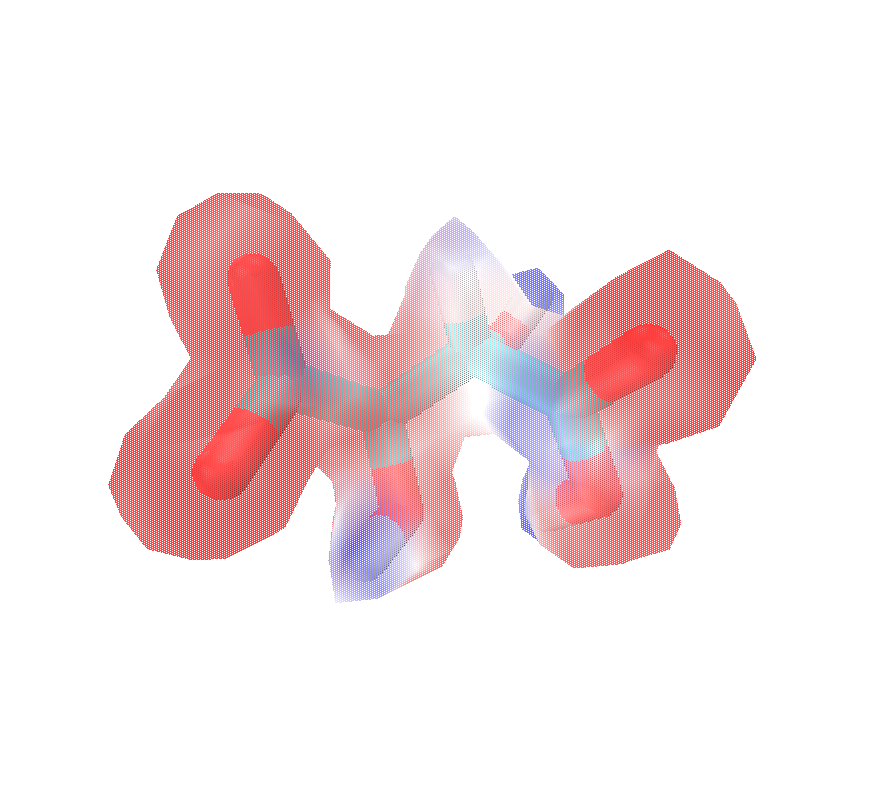

Supplement: SU-002-D3SU00412K-s004 [file SU-002-D3SU00412K-s004.zip › IonDescriptors/anions_MEP_processed/bit_ol.png]

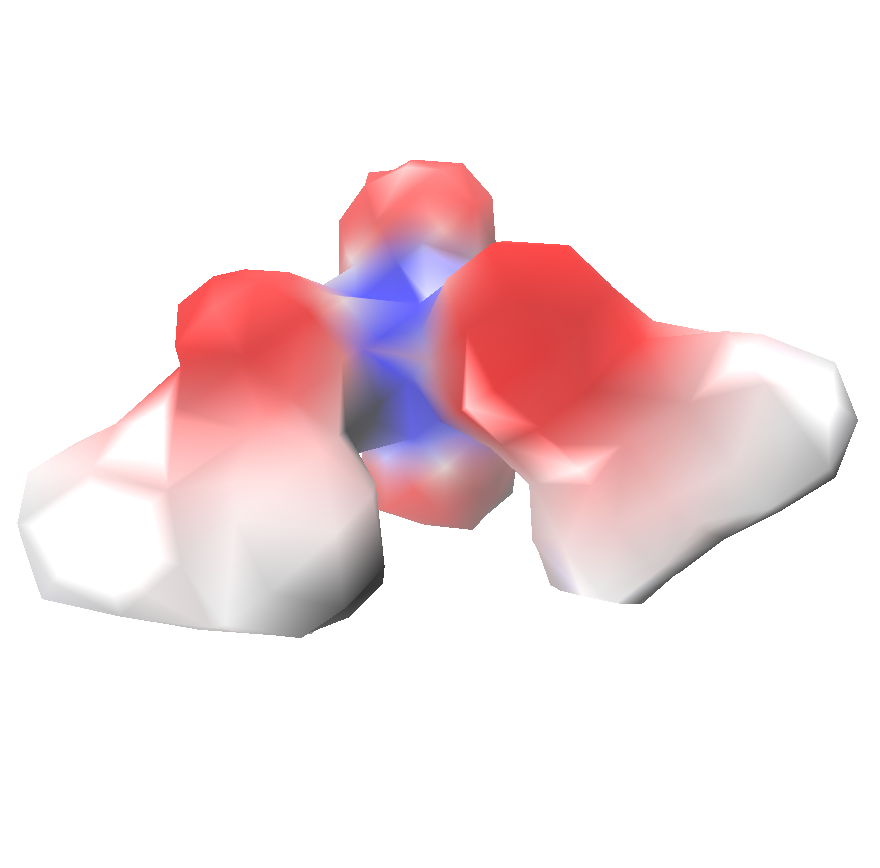

Supplement: SU-002-D3SU00412K-s004 [file SU-002-D3SU00412K-s004.zip › IonDescriptors/anions_MEP_processed/mop.png]

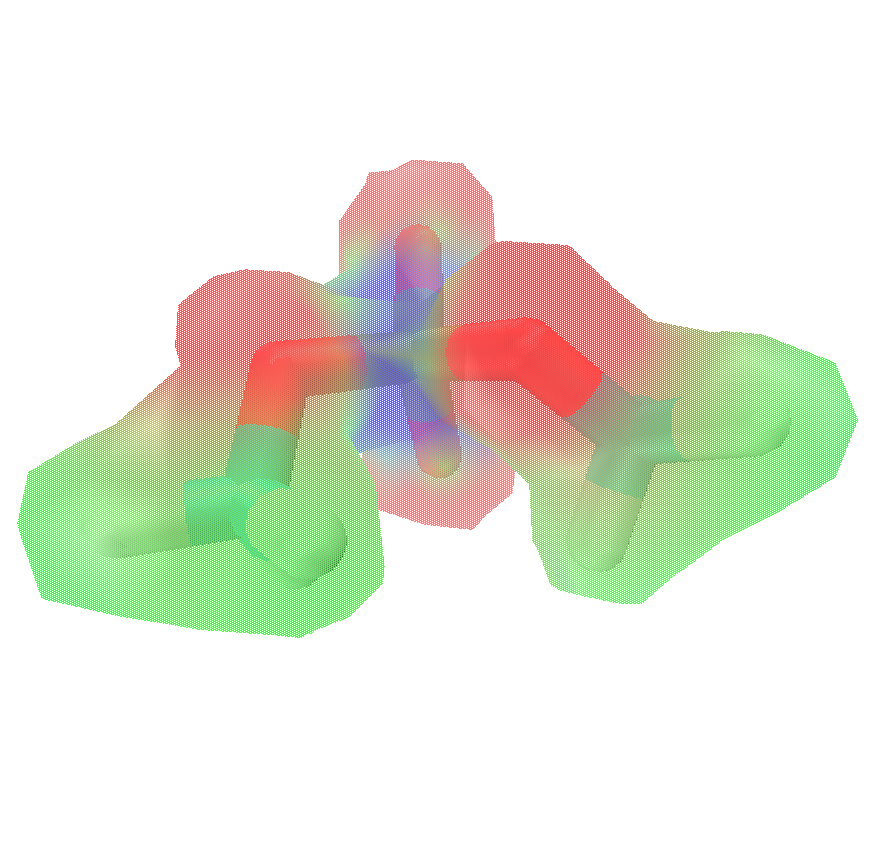

Supplement: SU-002-D3SU00412K-s004 [file SU-002-D3SU00412K-s004.zip › IonDescriptors/anions_MEP_processed/mop_ol_RGB.png]

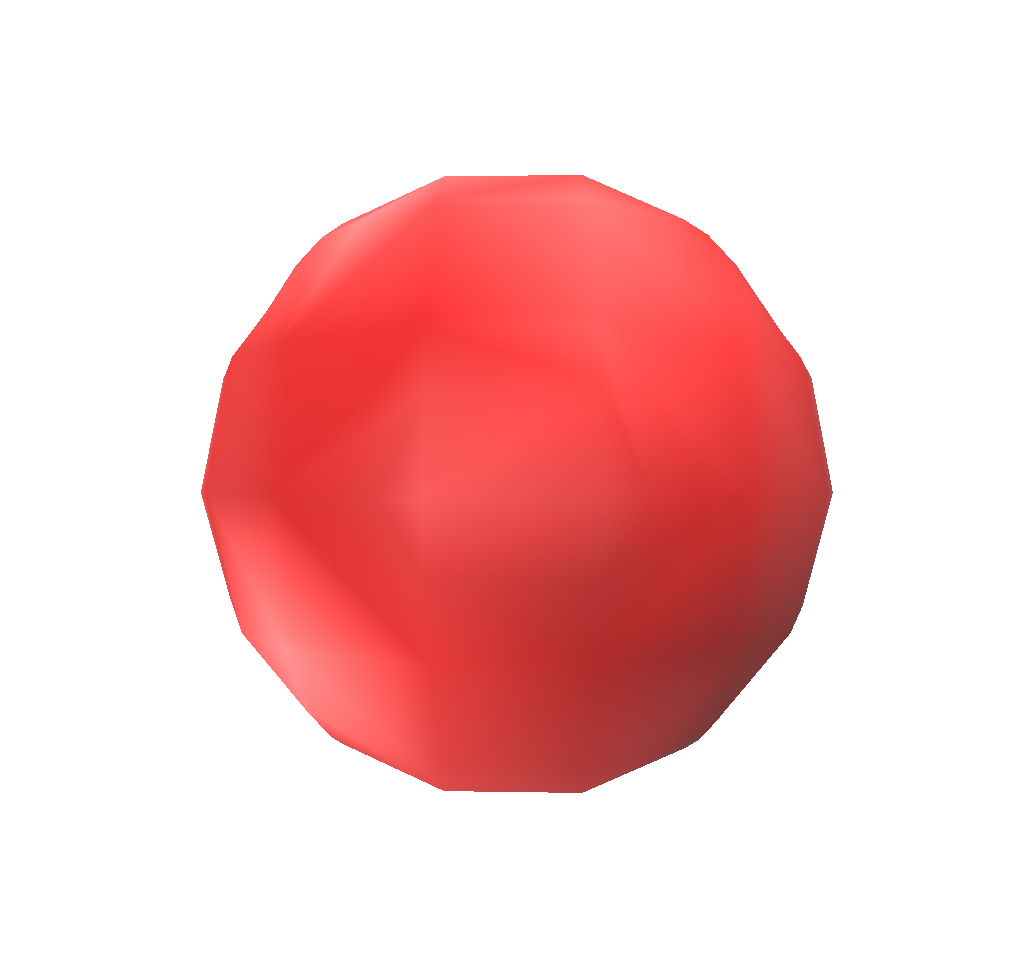

Supplement: SU-002-D3SU00412K-s004 [file SU-002-D3SU00412K-s004.zip › IonDescriptors/anions_MEP_processed/iod.png]

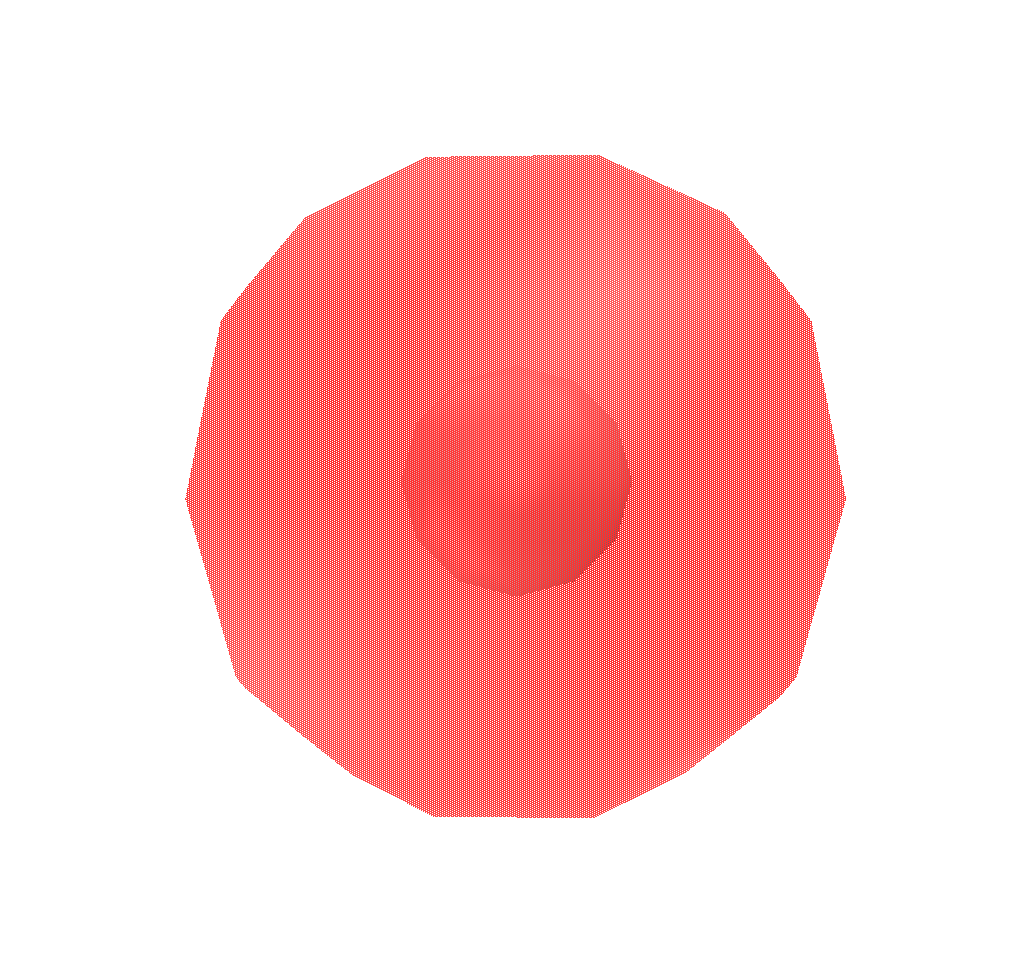

Supplement: SU-002-D3SU00412K-s004 [file SU-002-D3SU00412K-s004.zip › IonDescriptors/anions_MEP_processed/iod_ol.png]

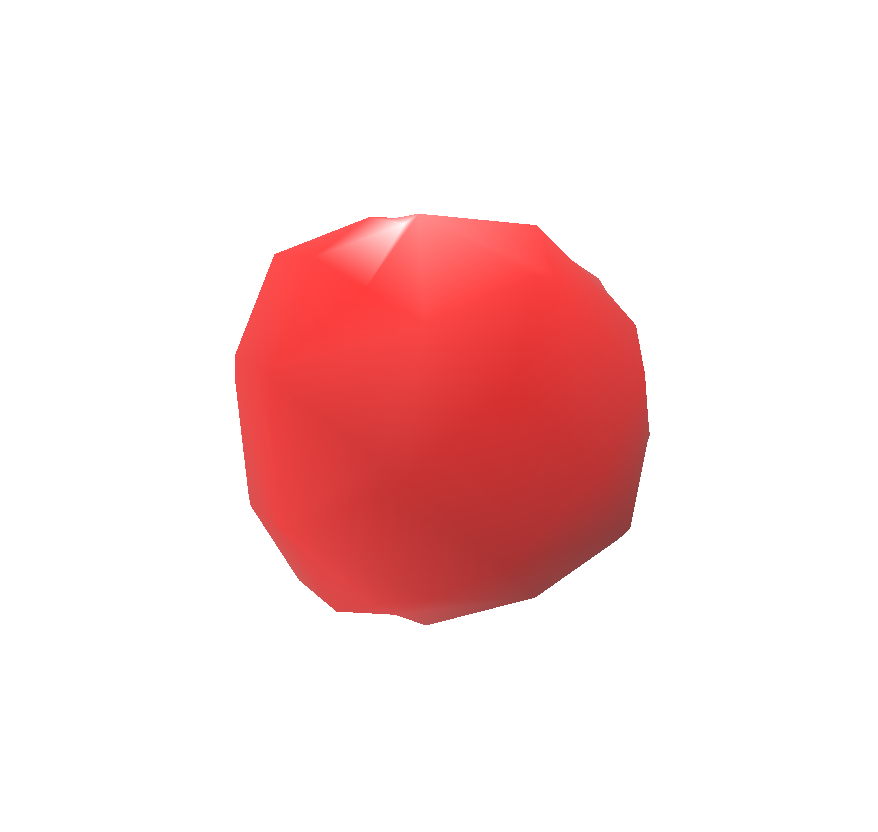

Supplement: SU-002-D3SU00412K-s004 [file SU-002-D3SU00412K-s004.zip › IonDescriptors/anions_MEP_processed/chl.png]

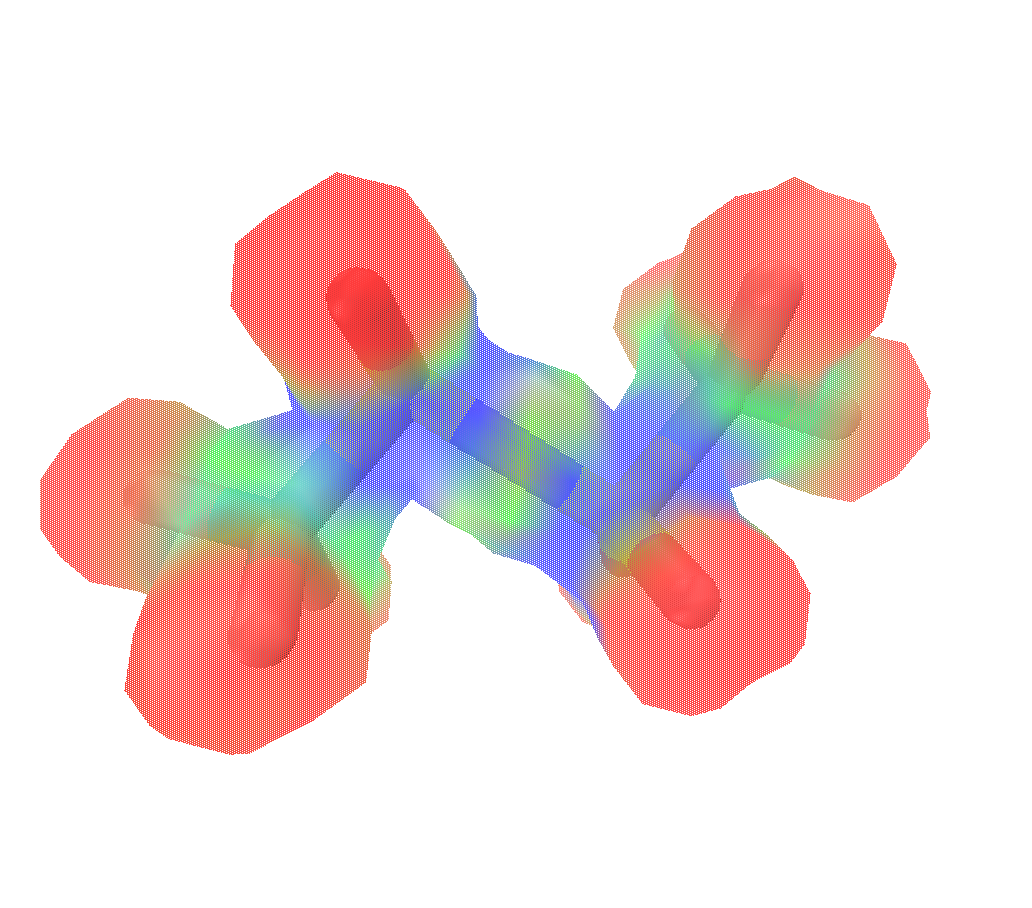

Supplement: SU-002-D3SU00412K-s004 [file SU-002-D3SU00412K-s004.zip › IonDescriptors/anions_MEP_processed/ntf_ol_RGB.png]

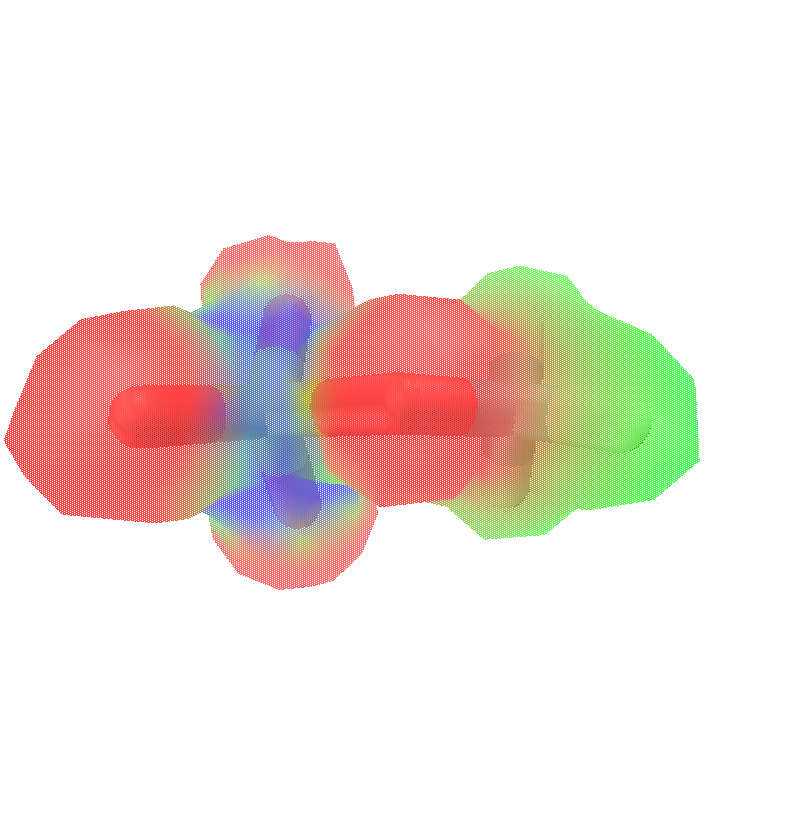

Supplement: SU-002-D3SU00412K-s004 [file SU-002-D3SU00412K-s004.zip › IonDescriptors/anions_MEP_processed/osm_ol_RGB.png]

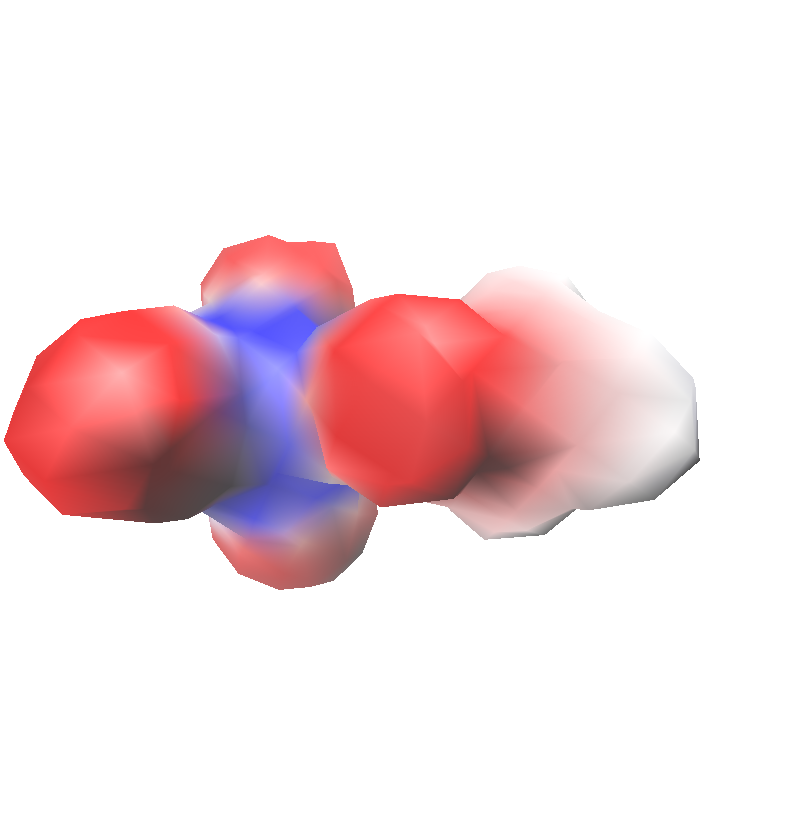

Supplement: SU-002-D3SU00412K-s004 [file SU-002-D3SU00412K-s004.zip › IonDescriptors/anions_MEP_processed/osm.png]

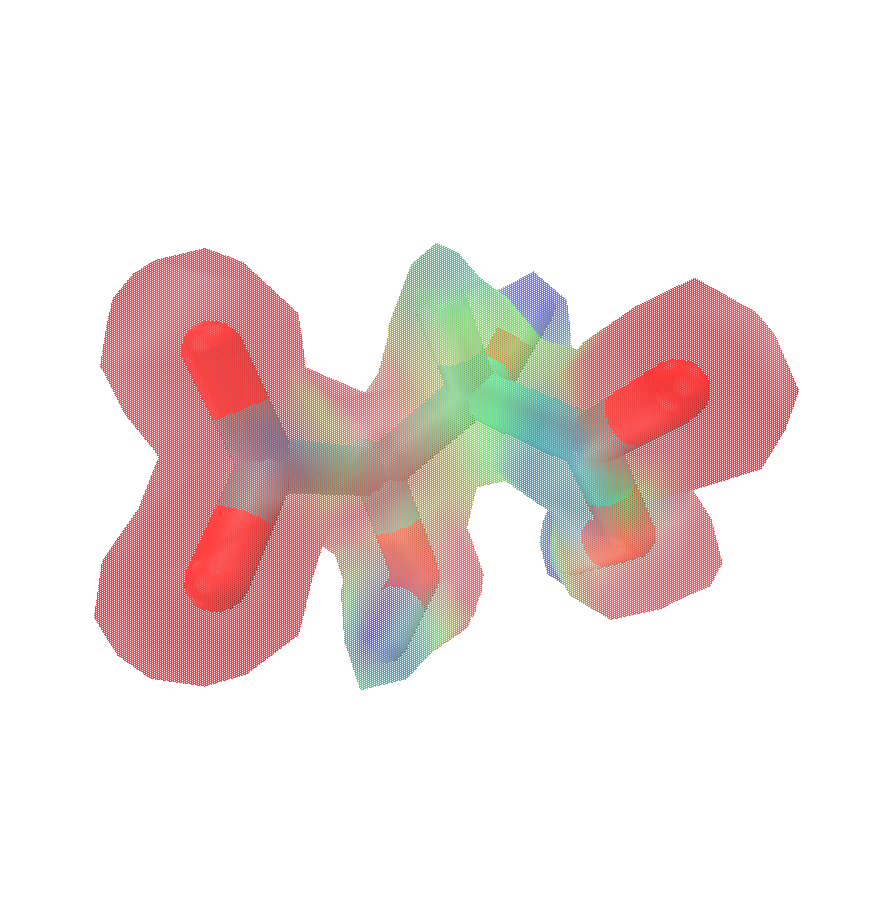

Supplement: SU-002-D3SU00412K-s004 [file SU-002-D3SU00412K-s004.zip › IonDescriptors/anions_MEP_processed/bit_ol_RGB.png]

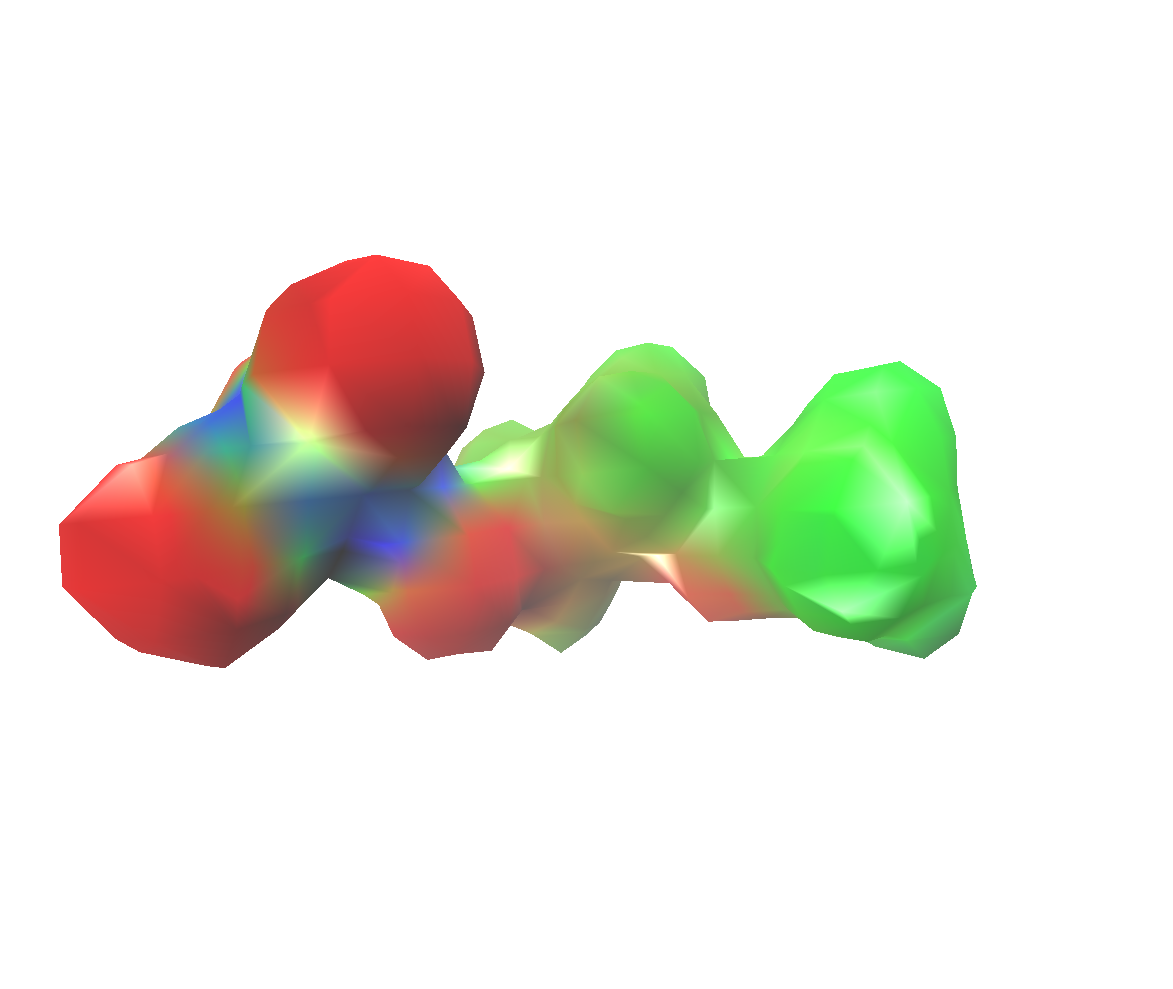

Supplement: SU-002-D3SU00412K-s004 [file SU-002-D3SU00412K-s004.zip › IonDescriptors/anions_MEP_processed/met_RGB.png]
